# Evaluation of the Alkaloid Secretions within the Millipede *Brachycybe* and Their Pheromone Properties

**DOI:** 10.1021/acs.jnatprod.6c00556

**Published:** 2026-07-21

**Authors:** Arden Hatch, Anne C. Jones, Luisa F. Vasquez-Valverde, Paige Banks, Isabel Gooding, Lisa Markoff, Paul E. Marek, Emily Mevers

**Affiliations:** † Department of Chemistry, 1757 Virginia Tech, Blacksburg, Virginia 24061, United States; ‡ Department of Entomology, Virginia Tech, Blacksburg, Virginia 24061, United States

## Abstract

*Brachycybe* is a genus of the millipede subterclass Colobognatha, a group that exhibits rare social characteristics, including species aggregations and male brood care. Although it has been hypothesized that alkaloid-based defensive secretions may also function as pheromones, no studies have examined these traits. Here, we evaluated the chemical secretions of six species, two of which had not been previously studied, and conducted Y-tube pheromone assays. Chemical investigations elucidated the structures of three new metabolites: hydrogosodesmine acid (**6**), homo-hydrogosodesmine acid (**7**), and buzonamine acid (**8**). Y-tube pheromone assays revealed that *Brachycybe producta* is significantly attracted to conspecifics (*p* = 0.0428) and is deterred by a different species of alkaloid-producing millipede (*p* = 0.0161). The former assay was repeated using a mixture of the crude (*p* = 0.0428) and purified (*p* = 0.0433) chemical defense secretion from*B. producta*. This study provides the first evidence that colobognath millipede chemical secretions serve both defensive and pheromone roles.

## Graphical abstract

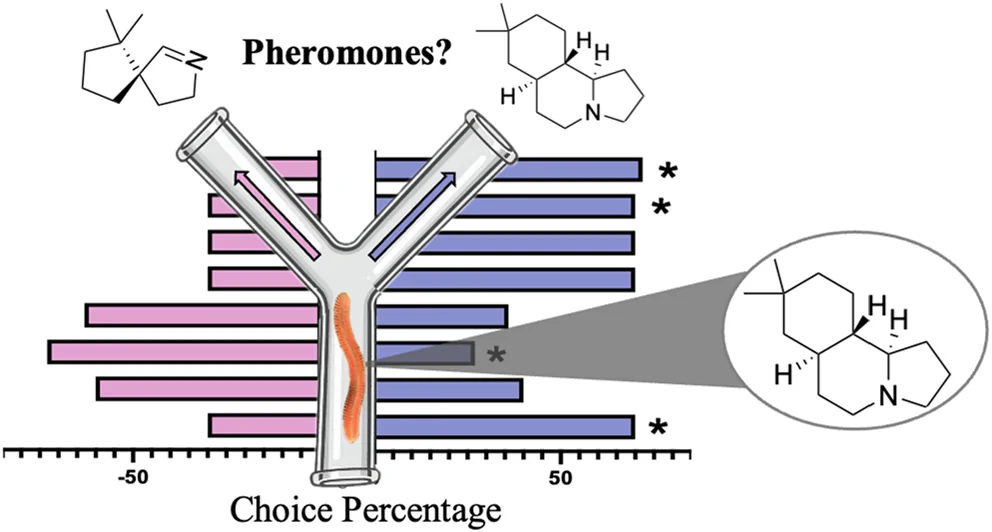

Arthropods are producers of semiochemicals (*e.g.*, allomones and pheromones) that mediate a variety of essential behaviors, including serving as chemical defense agents, promoting the formation of species-specific aggregations, alerting to unfavorable conditions, and mediating sexual behaviors.^1–4^ Chemical defense allomones have been characterized from all major subphyla,^5^ with many reports and review articles summarizing the findings within Hexapoda (*e.g.*, insects and springtails),^6,7^ Myriapoda (*e.g.*, millipedes and centipedes),^8–11^ and Chelicerata (*e.g.*, spiders, scorpions, and horseshoe crabs).^12,13^ Pheromones have been reported from social arthropods, most notably social insects,^14–16^ including bees, ants, and termites, but there are reports of pheromone use across all arthropod subphyla.^17–19^ It has been hypothesized that many of these semiochemicals are dual-functioning, serving both defensive and sexual roles, triggering alarm behavior, or promoting aggregation.^20,21^ However, only a handful of examples have been confirmed. This includes studies into *Aleochara curtula*, a beetle that secretes large quantities of three lipids [(*Z*)-4-tridecene, dodecanal, and (*Z*)-5-tetradecenal] to deter predators, but at lower concentrations, these lipids readily stimulate male copulatory behavior.^22^ The multifunctional roles of semiochemicals likely conserve the resources required to maintain diverse and costly biosynthetic machinery.^21^

Although social behaviors are rare within myriapods, Colobognatha, a subterclass of fungus-feeding millipedes, are known to form large species-specific aggregations and perform male brood care.^8,11,23–26^ Like most Myriapoda, members of Colobognatha produce defensive agents, stored in repugnatorial glands and exuded from ozopores upon physical disturbance.^8,27^ The chemical secretions of 12 genera have been defined, and all are believed to contain simple monoterpenes, while two of three orders (e.g., Polyzoniida, and Platydesmida) also produce more complex heterocyclic terpenoid alkaloids.^8,11^ A subset of the terpenoid alkaloids has been shown to disturb a variety of potential predators, including spiders, ants, and cockroaches, supporting a likely defensive role.^28–31^ However, the defensive secretions have recently been hypothesized to possibly serve as pheromones, promoting social traits.^8,32^ However, to date, no studies have focused on understanding the pheromone properties of Colobognatha defensive secretions.

*Brachycybe* is a genus of the order Platydesmida, comprising eight described species; however, this likely underrepresents the true species diversity.^23,24^ Millipedes within this genus are commonly referred to as “feather millipedes”, and they are relatively smaller and thinner than other Colobognatha species, typically growing to only a few centimeters in length. They are typically found on the undersides of decaying logs in humid regions across North America and Asia, likely originating in Northern California about 50 million years ago.^23^ The defensive secretions of four species have previously been studied, revealing that they produce fused heterocyclic alkaloids (Figure 1). Specifically, sister species, *Brachycybe producta* and *Brachycybe petasata*, produce the bicyclic gosodesmine analogs (**1**–**4**), where another pair of sister species, *Brachycybe lecontii* and *Brachycybe rosea*, produce the tricyclic deoxybuzonamine isomers (**5a** and **5b**). There have been no studies into the chemical secretions of*Brachycybe nodulosa* (Japan),*Brachycybe picta* (California),*Brachycybe disticha* (Taiwan), or*Brachycybe cooki* (China).

**Figure 1:**
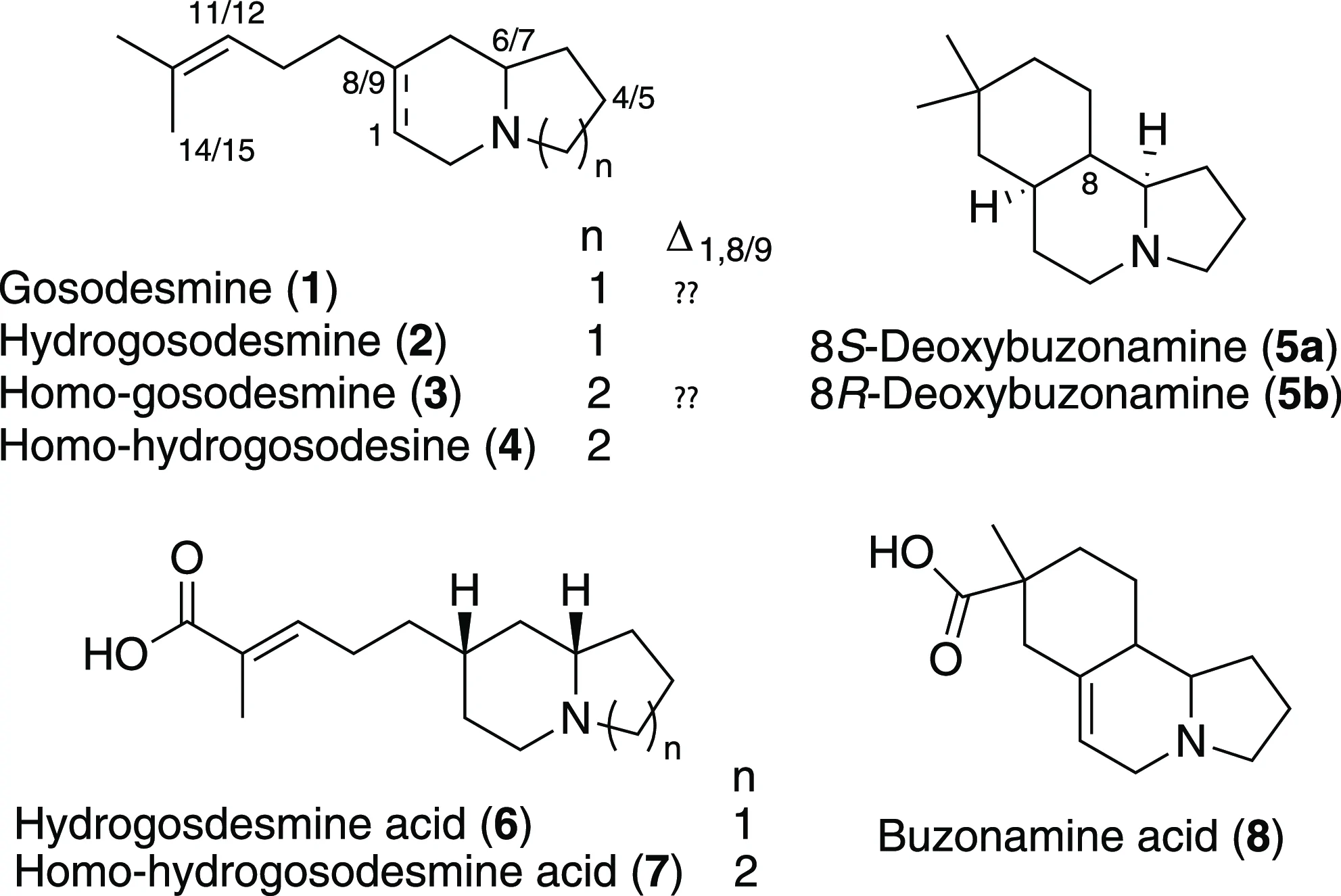
Chemical composition of the defensive secretions from *Brachycybe* species.

*Brachycybe*, like most Colobognatha, exhibits unique social behaviors, including the formation of large aggregations and male brood care. The aggregations are typically species-specific and are commonly associated with fungi on the underside of decaying logs.^24^ However, *Brachycybe* are believed to be generalists, as a detailed study into the dietary preference of *B. lecontii* determined it is associated with a wide variety of fungal orders.^24^ In addition, studies into two species,*B. nodulosa* and*B. lecontii*, found that males of both species display extensive brood care behavior.^26,27^ In both cases, after mating, the male curls his body around the egg clutch. The male remains in this posture throughout egg development (∼3–4 weeks), visibly grooming the eggs with his mouth and feet. Grooming is thought to protect the eggs from fungal pathogens and/or from predators.^27^ When *B. lecontii* brood males are physically separated from the egg clutches, the adult searches for and recollects the egg clutches.^26^ These behaviors are likely mediated by the production of yet-to-be-identified pheromones.

Here, we reanalyzed the chemical composition of the defensive secretions and phylogenetic relationship of six *Brachycybe* species: all five North American species (*B. lecontii*, *B. petasata*, *B. producta*, *B. rosea*, and *B. picta*) and *B. nodulosa* from Japan. This confirmed previous findings and led to the discovery of three new terpenoid alkaloids (**6**–**8**) (Figure 1). The new alkaloids represent the first Colobognatha-derived defensive secretions to incorporate a carboxylic acid moiety. In addition, Y-tube choice assays with *B. producta* confirmed that the defensive secretions have attractant properties, thereby providing the first supportive evidence that these semiochemicals are dual-functioning.

## Results and Discussion

### Chemical Investigations into the Chemical Secretions of Brachycybe Species

Five *Brachycybe* species were collected from various regions across North America, accordance with their seasonal patterns (Table S1), while a specimen of *B. nodulosa* from Japan was provided to us by the Virginia Tech Insect Collection. Defensive secretions were extracted from whole organisms using methanol to generate crude extracts. Analysis of the crude extract by low-resolution liquid chromatography mass spectrometry (LR-LCMS) and gas chromatography mass spectrometry (GCMS) confirmed the presence of gosodesmine analogs (**1**–**4**) in the glands of all millipedes except *B. lecontii*, and deoxybuzonamine isomers (**5a** and **5b**) in the glands of*B. picta*,*B. nodulosa*,*B. rosea*, and*B. lecontii* (Figures 2 and S1–S15 and Table S2). GCMS analyses revealed that only *B. lecontii* produces both stereoisomers of **5**, while *B. nodulosa* produces **5a**, and *B. rosea* and *B. picta* both produce **5b** (Figures S8–S15). It is important to note that our analysis of *B. nodulosa* was based on a single millipede sample; thus, this may not be representative of the species’ true metabolome. In addition to the previously reported alkaloids, most chromatograms of the crude extracts contain minor peaks that appear to represent structurally related alkaloids but that have distinct [M + H]^+^ at 236, 238, and 252 *m*/*z* (Figures S16–S18). Two of the peaks, 238 and 252 *m*/*z*, were present in LCMS chromatograms of crude extracts from all *Brachycybe* species, whereas the 236 *m*/*z* peak was detected only in crude extracts from *B. rosea*, *B. lecontii*, and *B. nodulosa*, three closely related species. These unknown analogs were prioritized for isolation by reverse-phase high-performance liquid chromatography (RP-HPLC) from chemical extracts of *B. rosea*, which yielded the highest quantities of all three targeted compounds. Acquisition of complete 2D NMR data sets (^1^H, dqfHSQC, H2BC, HMBC, and easyROESY) allowed for planar structure elucidation.

**Figure 2:**
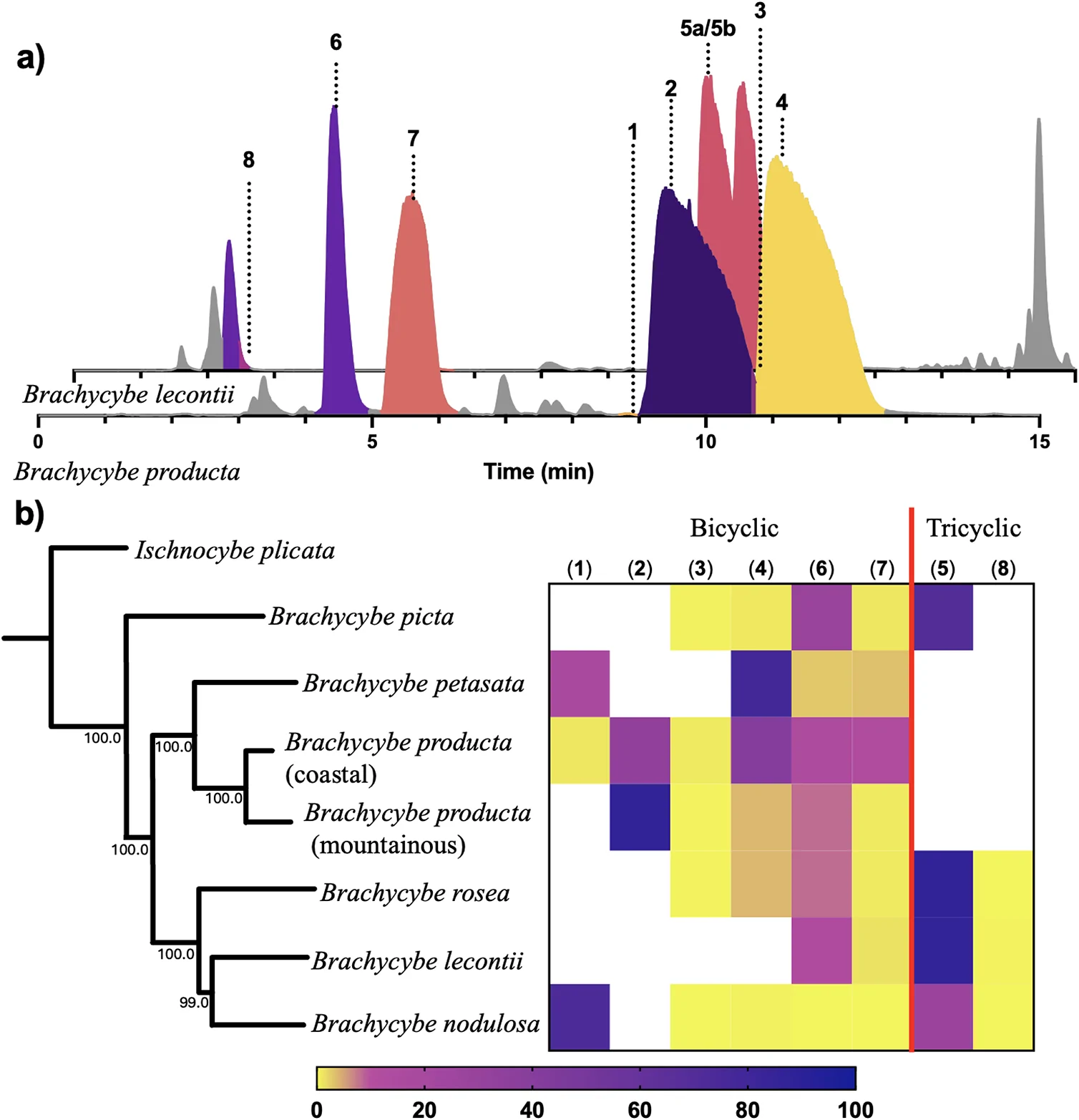
Relationships between the defensive secretions and phylogeny of six *Brachycybe* species. (A) Representative base peak chromatograms for **1**–**8** with hydrogosodesmine acid (**6**) in violet, buzonamine acid (**8**) in magenta, homo-hydrogosodesmine acid (**7**) in coral, deoxybuzonamine isomers (**5a**/**5b**) in pink, gosodesmine (**1**) in orange, hydrogosodesmine (**2**) in dark blue, homo-gosodesmine (**3**) in plum, and homo-hydrogosodesmine (**4**) in yellow. Peaks in gray represent uncharacterized metabolites. (B) Shows the phylogenetic relationship of *Brachycybe* species with relative quantification for each compound displayed in the heat map. The phylogenetic tree was estimated using a maximum-likelihood search, and node values are bootstrap support values for each clade.

### Structure Elucidation of Carboxylic Acid-Containing Terpenoid Alkaloids

High-resolution mass spectrometry (HRMS) of **6** ([M + H]^+^ 238.1801, Δ2.5 ppm) indicated a molecular formula (MF) of C_14_H_23_NO_2_, requiring four degrees of unsaturation (DoU). Analysis of the ^1^H NMR spectra revealed the presence of only a single methyl group at δ_H_ 1.72, representing the most significant difference from previously reported heterocyclic terpenoid alkaloids. Using H2BC and COSY correlations, it was determined that **6** contains the same bicyclic moiety as seen in **2** (Figures 3A and S19–S25 and Table S3); however, the *gem-*dimethyl moiety has been modified. HMBC correlations between the singlet methyl, H_3_-14, and carbons C-11 (δ_C_ 140.6), C-12 (δ_C_ 128.8), and C-13 (δ_C_ 169.7), support the oxidation of one of the *gem-*dimethyls in **2** to the carboxylic acid, forming an α/β-unsaturated carboxylic acid. Compound **7** had a similar fragmentation pattern to **6** and exhibited [M + H]^+^ of 252.1954 *m*/*z*, indicating an MF of C_15_H_25_NO_2_ (Δ1.6 ppm). Comparison of the ^1^H NMR spectra of **7** with that of **6** reveals that they are highly similar; however, **7** incorporates an additional methylene group. Analysis of the 2D NMR data set (Figures S26–S31 and Table S4) confirmed that **7** is the oxidized product of **4**.

**Figure 3:**
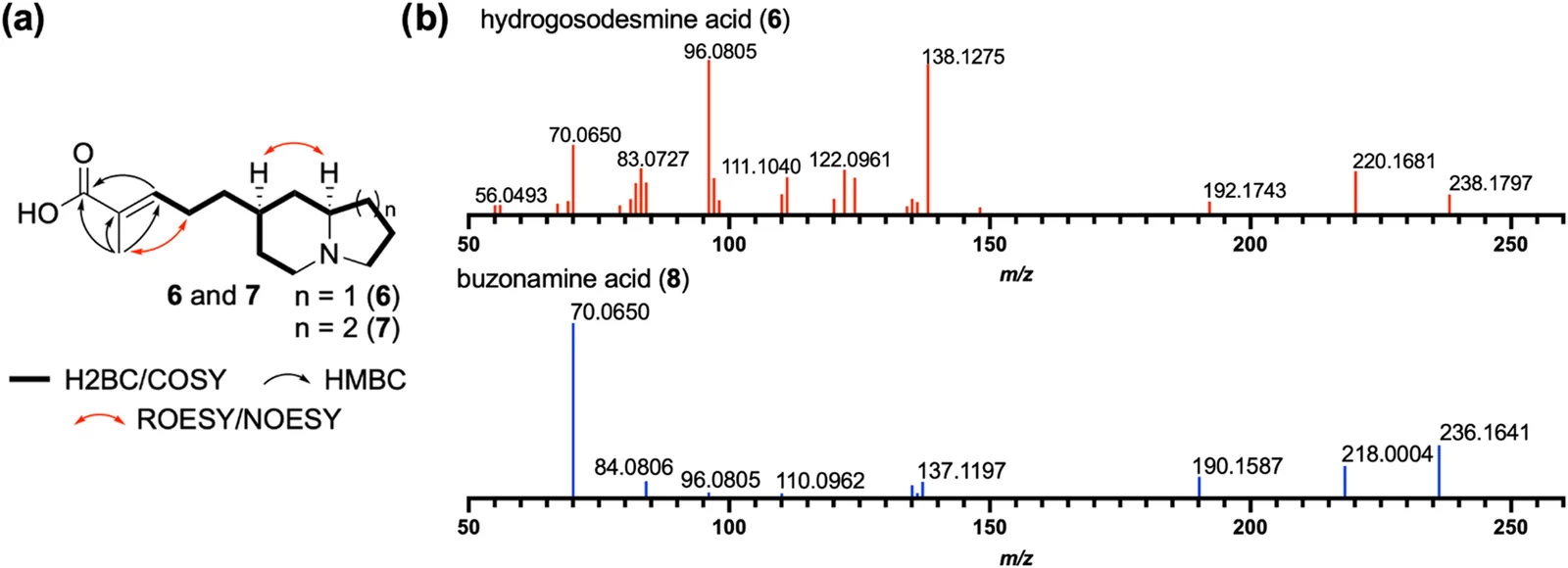
(A) Key 2D NMR correlations for hydrogosodesmine acid (**6**) and homo-hydrogosodesmine acid (**7**). (B) HR-LC-ESI-MSMS comparison between hydrogosodesmine acid (**6**; 80 eV) and buzonomine acid (**8**; 50 eV). Collision energies were selected so that the precursor ion for each compound had an approximate relative intensity of 10–20%.

The relative stereoconfiguration was assigned using 1D NOEs and comparison of ^13^C NMR chemical shifts with those of previously reported alkaloids. First, analysis of 1D NOE correlations for both **6** and **7** showed a strong correlation between the vinyl methyl group [H-14 (δ_H_ 1.72) in **6** and H-15 (δ_H_ 1.72) in **7**], and the allylic methylenes [H_2_-10 (δ_H_ 2.16) in **6** and H_2_-11 (δ_H_ 2.13) in **7**], indicating a *trans* configuration of the olefin (Figure 3A). Finally, the relative configuration of the bicyclic compound was assigned by comparison with synthetic analogs from a previous study,^33^ the only difference being the side-chain functionalization. The ^13^C NMR chemical shifts within the bicyclic moiety of both **6** and **7** strongly matched the *syn-*configuration with an average difference of 0.11 ± 0.14 for **6** (Table S5) and 1.68 ± 1.70 for **7** (Table S6), compared to the *anti-*configuration, which had an average difference of 2.75 ± 2.08 for **6** and 3.52 ± 2.17 for **7**. In support of a *syn*-configuration, both **6** and **7** exhibit positive specific rotations, matching the sign of the *syn*-configuration of the synthetic analogs. Thereby confirming a *syn-*configuration within the indolizidine and quinolizidine core.

Compound **8** exhibited [M + H]^+^ of 236.1644 *m*/*z*, indicating an MF of C_14_H_21_NO_2_ (Δ2.8 ppm), requiring 5 DoUs. Comparison of the fragmentation data of **8** with those of **6** and **7** revealed distinct differences (Figure 3B). Specifically, fragmentation of both **6** and **7** showed extensive side-chain fragmentation, absent in the spectra of **8**, suggesting that **8** lacked the same bicyclic scaffold. Analysis of the fragmentation of **8** confirms the presence of the carboxylic acid, with key fragments at 218.1535 and 190.1587 Da, but lacks any further side-chain alkyl groups, as seen in both **6** and **7**. This data suggests that **8** represents the carboxylic acid analog of deoxybuzonamine. Unfortunately, **8** was not abundant enough to isolate; however, it does represent the minor impurity seen in the spectra of **6**. Analysis of the limited 2D NMR correlations (Figure S32) representing **8** confirmed the presence of an olefin with carbons at δ_C_ 135.6 and 118.9, a singlet methyl group (δ_H_ 0.98; δ_C_ 19.7), and a strongly downfield quaternary carbon (δ_C_ 179.8). These chemical shifts are distinct from **6** and **7**, and indicate that the carboxylic acid is not in resonance with the olefin. Analysis of the HMBC revealed that the olefinic proton (δ_H_ 5.37) correlates with C-1 (δ_C_ 52.7), which is α to the tertiary nitrogen. This functionality is reminiscent of the ischnocybines produced by*Ischnocybe plicata*, a related platydesmidan species.^29^ This supports that **8** represents a new analog of the tricyclic alkaloids, in which one of the methyl groups in the*gem*-dimethyl moiety has similarly been oxidized to a carboxylic acid and may represent a biosynthetic intermediate of the andrognathanols,^28^ the only alkaloids with functionalization on the *gem-*dimethyl moiety.

Clear relationships between defensive secretions and molecular phylogeny were revealed through chemical compositional analysis using LR-LCMS. Overall, extracts from species within the same clade are more similar to each other. For example, both collections of *B. producta* (mountainous and coastal) and *B. petasata* belong to the same monophyletic group, and each only produced the bicyclic alkaloids with no detection of the tricyclic alkaloids (*e.g.*, **5** or **8**). Whereas the extracts of all other *Brachycybe* species (e.g., *B. lecontii*, *B. rosea*, *B. nodulosa*, and *B. picta*) contained large quantities of **5** but also had detectable quantities of many of the bicyclic alkaloids. Finally, molecular phylogeny shows that the production of both bicyclic and tricyclic alkaloids by *B. picta* may be symplesiomorphic (shared ancestral) within *Brachycybe*. Interestingly, extracts from all *Brachycybe* species contained two of the three carboxylic acid-containing alkaloids. This increase in structural diversity was likely previously overlooked for two reasons: (1) previous studies relied primarily on GCMS and the increased polarity of the carboxylic acid moiety interferes with detection by GCMS, and (2) each compound represents a minor analog.

### Y-Tube Choice Assays to Evaluate the Pheromone Properties of Millipede Chemical Secretions

The terpenoid alkaloids are known to be stored in repugnatorial glands and released upon physical disturbance, with a subset causing disorientation in common predators.^28,29,34^ Therefore, it is widely accepted that the alkaloids serve a defensive function for millipedes.^8^ However, colobognath millipedes are also well-known to display social characteristics, including aggregations and male brood care—phenotypes rare across the phylum Arthropoda. It has previously been hypothesized that the terpenoid alkaloids may also serve as pheromones, promoting the observed social characteristics.^8,23^ Some defensive allomones function as both alarm and defensive pheromones, likely in a concentration-dependent manner,^21^ including the production of benzaldehyde in*Streptogonopus phipsoni*, a polydesmidan millipede.^32^ Supporting this idea is the apparent relationship between the molecular phylogeny and the structural diversity of the chemical secretions. To test this function, Y-tube choice assays were employed using two *Brachycybe* species to assess the attractant properties toward conspecifics and repellent properties toward heterospecifics.

First, *B. producta* and *B. rosea* were independently evaluated for their attraction to conspecifics. These species were selected because they are commonly found year-round and can successfully be maintained in the laboratory for months. The assay setup involved trapping a single millipede or a group of millipedes in one arm of the Y-tube, while allowing another millipede to move freely throughout the entire enclosure (Figure S33). Y-tube size (5.5 in. length, 10 mm diameter) was determined based on field observations of aggregations and the ability of the millipede to move freely within the apparatus. The other arm of the Y-tube apparatus was left empty for all experiments. Platydesmidan millipedes live in darkness; therefore, assays were recorded under red light, and millipede movements were quantified from video analysis. Several properties were quantified, including the millipedes’ first choice, the total number of choices, and the total time spent in each arm of the Y-tube apparatus.

Analysis of the millipedes’ first choice (Figure 4), whether they moved toward a conspecific or to the empty arm, revealed that *B. producta* has a significant preference for conspecifics. *Brachycybe producta* chose to go toward a conspecific 70% of the time (*p* = 0.0428, *n* = 30), while *B. rosea* only chose to go toward its own 40% of the time (ns, *n* = 30) (Table S7). Although *B. rosea* did not choose to go toward a conspecific on its first choice most of the time, the millipede did travel the Y-tube arm containing its kin more times overall (*p* = 0.0004, *n* = 30) (Figure S34 and Table S8). This may indicate that other factors are influencing the initial choice, such as triggering an alarm pheromone when the millipede is placed in the Y-tube apparatus. In addition, increasing the group size of *B. producta* trapped in one arm of the Y-tube from 1 to 5 yielded similar results, with the millipedes moving toward conspecifics in 70% of the trials (ns, *n* = 10) (Figure 4 and Table S7). The millipede also spent significantly more time in the Y-tube arm containing the group of millipedes compared to the control experiment (*p* < 0.0001, *n* = 10) (Figure S34 and Table S9) and within the experiment (*p* = 0.0142, *n* = 10) (Figure S35 and Table S10).

**Figure 4:**
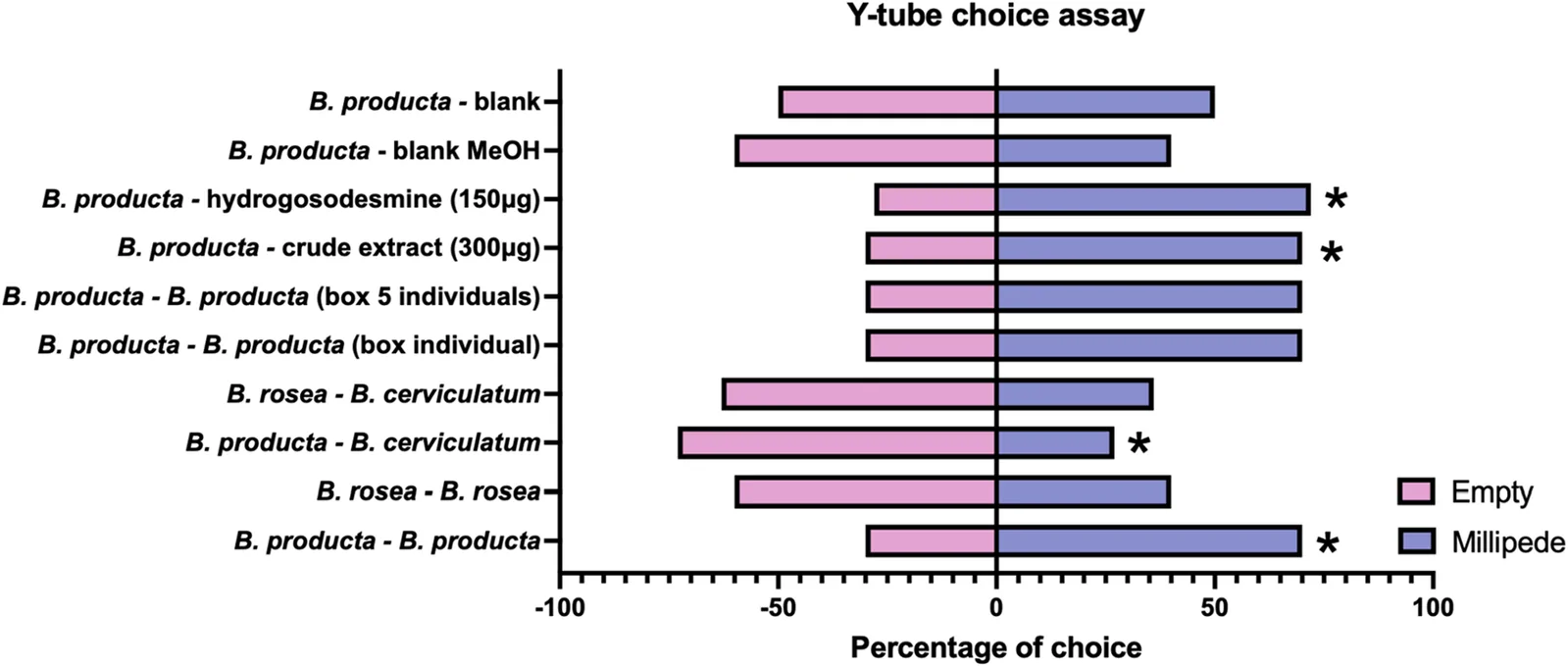
Y-tube choice assay indicating the percentage of choice to the effect and the empty side based on the first choice. Replicate number *n* = 30 for lines 1–4 and 7; *n* = 10 for lines 5, 6, 9, and 10; *n* = 25 for line 8. Two-sided binomial tests were parametrized with *p* (success) = 0.5 and an α threshold of 0.05. Significance for two-sided binomial tests is as follows from bottom to top (0.0428, 0.36, 0.0161, 0.2005, 0.3438, 0.3438, 0.0428, 0.0433, 0.36, and 1).

To confirm that the attractant properties are mediated by the chemical secretions, we evaluated the behavior of *B. producta* when exposed to either the crude extract or purified alkaloids. An aliquot (300 μg) of the crude material was impregnated on a paper disc and then placed into the same apparatus used in the live millipede assay. This quantity was selected because it represents the ecologically relevant amount of secretions within a single millipede.^28,29^ When *B. producta* was exposed to the disc impregnated with the crude extract, the millipede chose to go toward the crude extract on its first choice 70% of the time (*p* = 0.0428, *n* = 30) (Table S7). In addition, *B. producta* spent more time per choice (*p* = 0.0011, *n* = 30) (Figure S36 and Table S10), total time per arm (*p* < 0.0001, *n* = 30) (Figure S37 and Table S11), and had more approaches to the crude extract than the empty arm (*p* = 0.0055, *n* = 30) (Figure S38 and Table S12). Furthermore, repeating this experiment with 150 μg of purified **2**, the primary metabolite in *B. producta* defensive secretions, yielded the same attractive behavior, where the millipede went toward the impregnated disc 72% of the time (*p* = 0.0433, *n* = 30) (Table S7). *Brachycybe producta* also spent more time in the arm containing the impregnated disc per choice (*p* = 0.0174, *n* = 30) (Figure S36 and Table S10) and over the course of the experiment (*p* = 0.0392, *n* = 30) (Figure S37 and Table S11).

Finally, we examined interspecies interactions to determine if *B. producta* or *B. rosea* are attracted to distantly related alkaloid-producing millipedes.*Bdellozonium cerviculatum*, a member of the order Polyzoniida, is sympatric with both*B. producta* and*B. rosea*, but produces distinct alkaloids. *Bdellozonium cerviculatum* produces two previously reported spiro-alkaloids, polyzonimine and analogs of nitropolyzonimine (Figure S39).^32,34^ Repeating the Y-tube choice assay revealed that both *Brachycybe* species appeared to be repelled by *B. cerviculatum* (Figure 4), where *B. producta* chose to go toward the empty Y-tube arm 73% of the time (*p* = 0.0161, *n* = 30) and *B. rosea* went toward the empty Y-tube 63% of the time (ns, *n* = 30) (Table S7). *Brachycybe rosea* spent significantly more time per choice in the empty arm of the Y-tube apparatus (*p* = 0.0227, *n* = 30) (Table S10).

### Quantification of Brachycybe lecontii Secretions in Different Sexes

Members of Colobognatha are well-known for performing brood care, where the male curls his body around the egg clutch while grooming the eggs for the entire egg development period.^23,25,26^ This distinct behavior led to the hypothesis that these compounds could potentially serve as sex pheromones. To quantify secretions in different sexes, individual *B. lecontii* millipedes were collected and preserved in methanol during their reproductive season (late April–early July).^26^ A total of 50 females, 26 males, and 15 brood males (males actively clutching eggs) were collected. It is noteworthy that none of the *B. lecontii* individuals were found in aggregations during this time period. LR-LCMS analysis of the individual extracts revealed that all contained **5**, and a majority produced **6** and **7**. Relative quantification (Figure 2) of all individual millipedes normalized to millipede length revealed that **5** is produced in the highest quantity (∼85%), with moderate levels of **6** (∼12.9%), and trace amounts of both **7** and **8**. Although response factors varied between **1**–**8** (Figure S40), all extracts were processed under the same conditions, allowing for direct comparison between the sexes. Kruskal–Wallis tests for each metabolite did not identify any differences between the sexes or between any two groups when analyzing the quantity of **5**–**8** (Figure 5A). GCMS analysis of the ratio of each stereoisomer of **5** within the individual extracts revealed that the majority (83%) of *B. lecontii* individuals produced only one isomer, while 16% produced both isomers in varying proportions (Table S13). Interestingly, 92% of nonbrood males produced **5b** (Figures 5B and 4C and Table S14), which is significantly more than either females (60%; *p* = 0.0032) or brood males (53%; *p* = 0.0065). Alkaloid composition did not differ between females and brood males.

**Figure 5:**
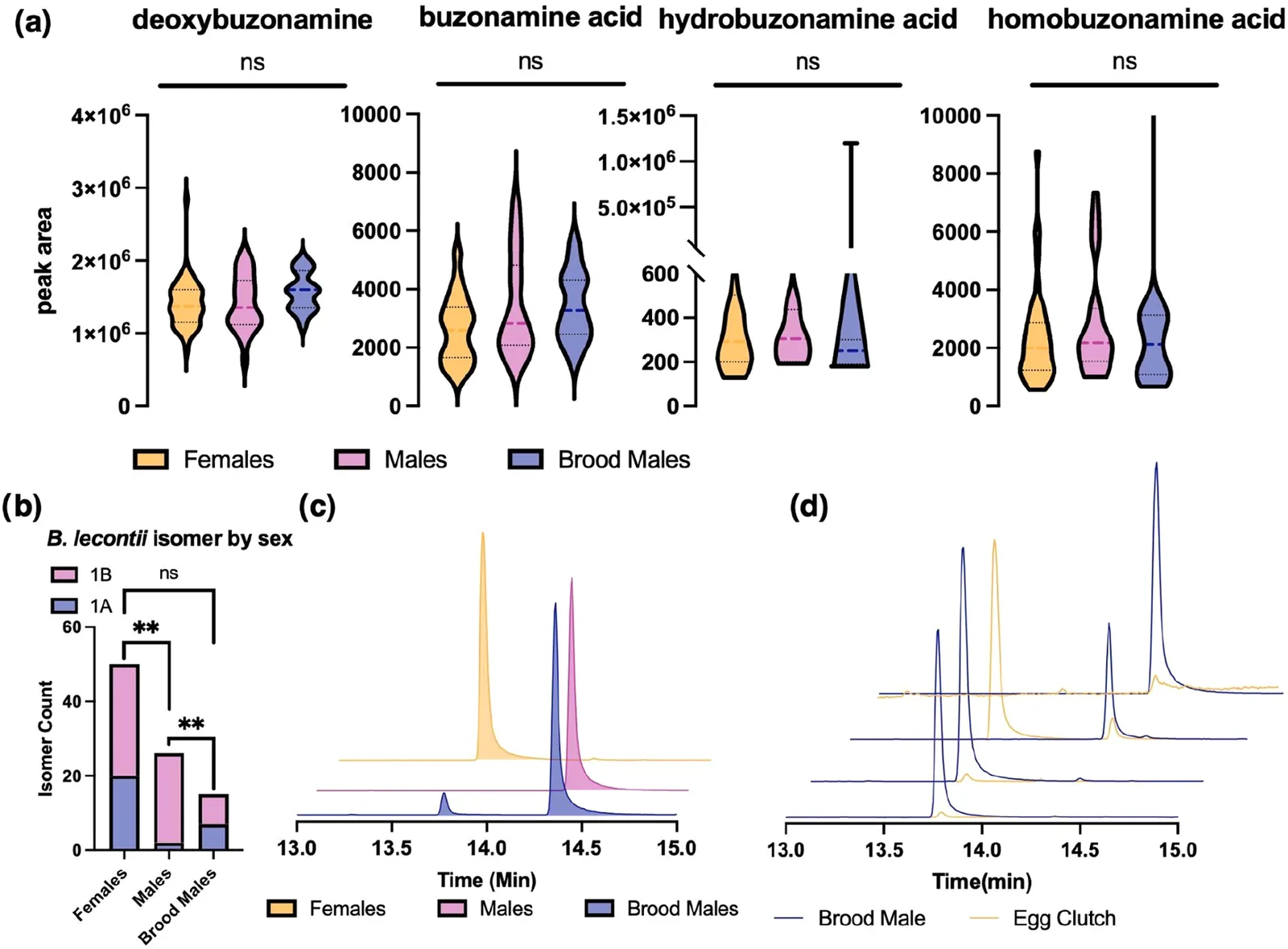
Ecological roles for secretions in*Brachycybe lecontii*. (A) Violin plots showing peak areas for *B. lecontii* secretions. Kruskal–Wallis tests found no significant differences in any of the metabolites (**5a**/**5b**: *p* = 0.1388, **6**: *p* = 0.0741, **7**: *p* = 0.5166, and **8**: *p* = 0.6638), (B) proportion of **5a** and **5b** produced by females (*n* = 50), males (*n* = 26), and brood males (*n* = 15). Males were found to be significantly different from females (*p* = 0.0032) and brood males (*p* = 0.0065), while females were not significantly different from brood males (*p* = 0.7673) using Fisher’s exact test, (C) GCMS chromatogram of *B. lecontii* individuals showing retention time differences between deoxybuzonamine isomers **5a** and **5b**, and (D) GCMS chromatograms of*B. lecontii* brood males and their associated egg clutches.

Previously, it had been reported that chemical secretions are not detected until they reach the stadium II developmental stage of maturity;^26^ therefore, implying that egg clutches may acquire the alkaloids from the brood male. However, analysis of 15 egg clutches and corresponding brood males revealed intriguing discrepancies in alkaloid production. Of the 15 egg clutches analyzed, only seven of the egg clutches had detectable alkaloids, and surprisingly, only one of these matched the isomer detected in the extract of the associated brood male. The other six egg clutches either contained the opposite isomer or both isomers (Figure 5D and Table S15). This raises questions about whether the brood male supplies alkaloids to the eggs or whether the eggs can produce their own alkaloids. It is conceivable that the eggs can biosynthesize the alkaloids, the female may apply the alkaloids during oviposition, and/or the brood male might be able to selectively apply a single isomer to the egg clutches. Regardless, further studies are needed to elucidate the ecological role of compounds associated with the eggs in this system.

In summary, this study sheds light on a potential dual role of terpenoid alkaloids as both defensive agents and pheromones. Evaluation of the chemical secretions of six *Brachycybe* species using LCMS and GCMS has provided a deeper understanding of the true chemical diversity of alkaloids within this species-rich genus and led to the discovery of three new terpenoid alkaloids. Each of the new alkaloids incorporates a carboxylic acid moiety, a functionality not observed in any previous colobognath alkaloids. In addition, Y-tube choice assays have provided the first confirmation that the alkaloids are dual-functioning semiochemicals, serving both defensive and pheromonal roles. The attractant behavior was replicated using both the crude extract and the purified defensive agent. This study establishes a foundation for further studies into the pheromone properties of defensive secretions across the entire phylogeny of Colobognatha.

## Experimental Section

### General Experimental Procedures

Optical rotation was measured using a JASCO P-2000 polarimeter. NMR spectra were recorded with deuterated DMSO with the residual solvent peak as an internal standard (δ_C_ 39.5, δ_H_ 2.50) on a Bruker Avance III 600 MHz instrument equipped with a triple resonance inverse (CP-TCI) Prodigy N2 cooled CryoProbe. GCMS analyses were carried out using an Agilent 5975C VL MSD GCMS that was equipped with an electron ionization (EI) ion source. LR-LCMS data were obtained using an Agilent 1200 series HPLC system equipped with a photodiode array detector and a Thermo LTQ mass spectrometer. HR-ESI-MS data were obtained using a Vanquish UHPLC system coupled to an Exploris 120 Orbitrap mass spectrometer (Thermo Fisher Scientific). HPLC purifications were performed using Agilent 1200 Series or 1260 Infinity II HPLC systems equipped with a photodiode-array detector. All solvents were of HPLC quality.

### Millipede Collections

All millipedes were hand-collected from the undersides of woody debris and logs using soft forceps (Table S1). *Brachycybe lecontii* and *B. petasata* are commonly found in the Appalachian Mountains between April and October.^24^ *Brachycybe rosea* is located in the Sierra Nevada Mountain Range and is most commonly encountered between October and April. *Brachycybe picta* is observed year-round in the redwood forests around San Francisco Bay. *Brachycybe producta* is sympatric with both *B. rosea* and *B. picta*, occurring in both the Sierra Nevada Mountains and Coast Range, but there are no observations in the Sacramento Valley, which lies between these two sites. With two distinct geographic ranges, collections from each region were kept separate in this study and named by their collection site (*e.g.*, “mountainous” and “coastal”). All *Brachycybe* species were found on the undersides of various decaying hardwoods; however, *B. picta* was found exclusively on the undersides of decaying redwood logs. A single sample of *B. nodulosa*, native to Japan, is from the Virginia Tech Insect Collection.

### Millipede Extraction

The alkaloids within entire colonies, individual adults, and eggs were extracted using methanol (MeOH). Specimens were decanted and resuspended in MeOH for preservation. Male, female, and brood male individuals were collected into individual 1-dram vials containing 0.5 mL of MeOH. Males and females were determined to be of sexual maturity by body ring count (35 and 39 rings, respectively).^26^ Voucher Specimens have been deposited as natural history specimens in the Virginia Tech Insect Collection (https://collection.ento.vt.edu/).

### General Analytical Analysis of Defensive Gland Secretions

#### LR-LC-LTQ-MS Method

A Kinetex 5 μm EVO C18 (100 mm × 4.6 mm) column was used with the following conditions: holding 20% MeCN + 0.1% formic acid (FA)/80% H_2_O + 0.1% FA for three min, followed by a linear gradient to 50% MeCN + 0.1% FA/50% H_2_O + 0.1% FA over 20 min with a flow rate of 0.3 mL/min. Data was processed in Freestyle software (v1.6) unless otherwise specified.

#### HR-LC-Orbitrap-MS Method

A Kinetex 2.6 μm Polar C18 (2.1 mm × 100 mm) column was used with the following conditions: hold 5% MeCN + 0.1% formic acid (FA)/95% H_2_O + 0.1% FA for one min, followed by a quick linear gradient to 25% MeCN + 0.1% FA/75% H_2_O + 0.1% FA over 0.1 min then a slow linear gradient to 100% MeCN + 0.1% FA over 6.9 min, which was held for 2.5 min, with a flow rate of 0.5 mL/min. MS analysis was performed using heated electrospray ionization (HESI) in positive ionization mode. The parameters were set as follows: Sheath gas to 50, auxiliary gas at 10, sweep gas at 2, spray voltage to 3.5 kV, ion transfer tube to 350 °C, and vaporizer to 150 °C. An MS scan range of *m*/*z* 100–1500 was used, with an expected peak width of 6s and a resolution of 60,000, with 1 microscan. The automatic gain control (AGC) target was set to 1E6 with a maximum injection time of 100 ms. A targeted mass inclusion list was provided. MS/MS spectra were acquired at a resolution of 15,000, with 1 microscan, a maximum injection time of 100 ms, and an AGC target of 1E5. The isolation window was set to 2 *m*/*z*, and the isolation offset was turned off. Collision energies between 45 and 80 eV were selected so that the precursor ion for each compound had an approximate relative intensity of 10–20%, ensuring complete fragmentation without losing information about the precursor ion. Data were processed in Freestyle software (v1.8) unless otherwise specified.

#### GCMS Method

A Cyclosil B chiral column (Agilent Technologies J&W Scientific, 30 m × 0.25 mm) was used, and the initial oven temperature of 60 °C was maintained for 1 min, followed by a ramp of 10 °C/min to 250 °C and then held for 4 min at 250 °C. Data was processed in MSD ChemStation E. software (v02.02.1431).

### Quantitative Analysis of Alkaloid Secretions by LCMS

Millipedes (>20 millipedes of varied sizes) belonging to each of the *Brachycybe* species, except *B. nodulosa* where a single millipede specimen was used, were extracted with 10 mL of MeOH for 8 h at rt (×3). After each extraction, the MeOH was decanted, transferred to a new 40 mL vial, and dried under vacuum. The combined extract was resuspended in 3 mL of MeOH, and an aliquot (5 μL) of each crude extract was analyzed as described above under “LR-LC-LTQ-MS Method”. Raw LCMS files were converted to mzML using MSConvert (v3.0.20337) and analyzed in MZmine 3 (v4.4.3). Peak detection was performed according to the protocol outlined in Banks 2025,^28^ with the percentage of each metabolite calculated based on the total peak area.

### Quantitative Analysis of Deoxybuzonamine Isomers by GCMS

The combined extract was resuspended in 3 mL of MeOH, and an aliquot (5 μL) of each crude extract was analyzed as described above under “GCMS Method”. Pure standards of isomers **5a** and **5b**, generously provided to us by Tappey Jones,^35^ were used to compare retention times and EIMS fragmentation patterns with peaks detected in *B. rosea*, *B. picta*, and *B. nodulosa* extracts.

### Extraction and Purification of New Alkaloids from *Brachycybe rosea*

The alkaloid secretions from ∼2.2 g of *B. rosea* millipedes were extracted with 10 mL of MeOH for 8 h at rt (×3). After each extraction, the MeOH was decanted, transferred to a new 40 mL vial, and dried under vacuum. The crude extract was resuspended in 3 mL of 1:1 MeOH/H_2_O, and individual compounds were purified by RP-HPLC. The HPLC was equipped with a Phenomenex 4 μm Hydro (250 mm × 10 mm) column and run under the following conditions: holding 50% MeCN + 0.1% FA/50% H_2_O + 0.1% FA for 5 min, followed by a linear gradient to 100% MeCN + 0.1% FA over 30 min at a flow rate of 3 mL/min. This yielded pure **6** (∼5 mg) and **7** (∼3 mg).

#### Hydrogosodesmine Acid (**6**)

pale yellow amorphous solid; [α]_D_ +16.5 (21.8 °C, 0.007 g/100 mL, MeOH); ^1^H NMR (DMSO-*d*_6_, 600 MHz): δ_H_ 6.60 (t, *J =* 6.8, H-11), 3.01 (m, H-2a), 2.96 (m, H-3a), 2.16 (m, H_2_-10), 2.04 (m, H-3b), 1.97 (m, H-2b), 1.85 (m, H-6), 1.81 (m, H-7a), 1.79 (m, H-5a), 1.72 (s, H_3_-14), 1.64 (m, H-1a), 1.63 (m, H-4), 1.33 (m, H_2_-9), 1.28 (m, H-8), 1.27 (m, H-5b), 1.14 (m, H-1b), 0.83 (m, H-7b); ^13^C NMR (DMSO-*d*_6_; through HSQC and HMBC): δ_C_ 169.7 (C-13), 140.6 (C-11), 128.8 (C-12), 63.4 (C-6), 52.7 (C-3), 51.2 (C-2), 36.3 (C-7), 35.1 (C-8), 34.8 (C-9), 31.1 (C-1), 29.6 (C-5), 25.2 (C-10), 20.4 (C-4), 12.0 (C-14). ESI MS/MS (orbitrap; CID 80 eV) *m*/*z* 238.1797 (13.2), 220.1691 (28.2), 192.1743 (8.7), 148.1118 (4.9), 138.1275 (97.16), 136.1118 (8.4), 135.0140 (10.5), 134.0962 (5.7), 124.1118 (24.0), 122.0961 (29.1), 120.0805 (10.4) 111.1040 (24.5), 110.0962 (13.4), 98.0962 (9.6), 97.0883 (23.9) 96.0805 (100), 84.0806 (20.9), 83.0727 (30.1), 82.0649 (20.7), 81.0697 (10.3), 79.0541 (6.3), 70.0650 (45.3196), 69.0572 (9.0), 67.0541 (7.4), 56.0493 (6.6), 55.0541 (6.5). HREIMS [M + H]^+^ *m*/*z* 238.1801 (calcd C_14_H_23_NO_2_, [M + H]^+^ 238.1807 Δ2.5 ppm)

#### Homo-hydrogosodesmine Acid (**7**)

amorphous solid; [α]_D_^22^ +2.8 (*c* 0.010, MeOH); ^1^H NMR (DMSO-*d*_6_, 600 MHz): δ_H_ 6.60 (t, *J* = 6.7, H-12), 2.75 (m, H-3a), 2.73 (m, H-2a), 2.13 (m, H_2_-11), 1.95 (m, H-2b), 1.93 (m, H-3b), 1.73 (m, H-7), 1.72 (s, H_3_-15), 1.63 (m, H-5a), 1.61 (m, H-1a), 1.56 (m, H-8a), 1.55 (m, H-4a), 1.50 (m, H-6a), 1.43 (m, H-4b), 1.28 (m, H_2_-10), 1.26 (m, H-9), 1.16 (m, H-5b) 1.13 (m, H-6b), 1.13 (m, H-1b), 0.85 (m, H-8b); ^13^C NMR (DMSO-*d*_6_; through HSQC and HMBC) δ_C_ 168.4 (C-13), 140.8 (C-12), 127.9 (C-13), 61.3 (C-7), 55.3 (C-2), 55.1 (C-3), 38.4 (C-8), 34.8 (C-9), 34.6 (C-10), 32.4 (C-6), 31.2 (C-1), 25.1 (C-4), 25.0 (C-11), 23.6 (C-5), 12.0 (C-15). ESI MS/MS (orbitrap; CID 80 eV) *m*/*z* 252.1954 (21.4), 234.1848 (43.3), 206.1900 (14.8), 162.1275 (4.9), 152.1431 (100) 150.1275 (10.4) 149.1197 (10.1), 148.1119 (5.2), 138.1275 (46.4), 136.1119 (34.4), 134.0962 (9.5), 125.1197 (4.2), 124.1119 (30.3), 123.1041 (5.0), 122.0963 (9.4), 112.1119 (8.3), 111.1040 (56.6), 110.0962 (87.0), 107.0854 (5.0), 105.0698 (5.2), 98.0962 (16.4), 97.0884 (94.8), 96.0806 (57.7), 95.0853 (4.8), 93.0697 (8.4), 91.0541 (5.0), 84.0806 (47.2), 83.0728 (25.2), 82.0650 (23.6), 81.0697 (14.4), 79.0541 (10.7), 70.0650 (14.7), 69.0572 (22.5), 67.0541 (12.2), 56.0494 (10.4), 55.0541 (8.3), 55.0416 (5.2). HREIMS [M + H]^+^ *m*/*z* 252.1954 (calcd C_15_H_25_NO_2_, [M + H]^+^ 252.1958 Δ1.6 ppm)

#### Buzonamine Acid (**8**)

ESI MS/MS (orbitrap; CID 50 eV) *m*/*z* 236.1641 (30.4), 218.1535 (18.7), 190.1587 (12.5), 137.1196 (9.4), 136.1118 (2.9), 135.1040 (7.5), 110.0962 (2.9), 96.0805 (3.4), 84.0806 (9.9), 70.0650 (100). HREIMS [M + H]^+^ *m*/*z* 236.1644 (calcd C_14_H_21_NO_2_, [M + H]^+^ 236.1645 Δ2.8 ppm).

### Y-Tube Choice Assays with Millipedes and Chemical Secretions

Choice experiments were conducted using a closed Y-tube system with a maintained airflow (Figure S33). All experiments were performed under red light to replicate aphotic conditions and were videographed throughout the assay (45 min). The size of the Y-tube (5.5 in. length, 10 mm diameter) was determined based on two factors. First, field observations indicated that *Brachycybe* aggregations are relatively small, typically measuring 1–3 in. in diameter; therefore, the apparatus’s 5.5 in. length is sufficient to replicate ecological conditions. Second, a 10 mm diameter was chosen to allow the individual millipede to move freely through the tube. The Y-tube was attached to an air pump with a flowmeter set to 0.5 L/min, and air was pumped into the T-junction to split the flow equally between the Y-tube’s arms. Wild-caught *B. rosea* and *B. producta* millipedes were kept in conspecific groups in the laboratory. Millipedes were kept in an enclosure (8 × 6 × 6 in. plastic bin) containing leaf litter and decaying wood. Only adult millipedes were selected for the Y-tube assay, and each was used no more than once per day. Following an experiment, millipedes were returned to their enclosures.

#### Assay Setup

One adult millipede was placed in the main arm of the Y-tube. A second millipede was enclosed in a wire mesh cup in one of the arms of the Y-tube to prevent movement (Figure S33). Assays with chemical secretions were performed by dissolving the crude extract or purified material in MeOH at 15 mg/mL, then loading 20 μL onto a filter paper disc. The impregnated disc was allowed to dry for 5 min, then placed in the same wire mesh cup used in the millipede assay. To avoid bias between Y-tube arms, the millipede or compound disk arm was alternated for each subsequent experiment. Following each replicate, the mesh and the Y-tube were washed with acetone and dried in an oven for at least 1 h.

#### Data Analysis

Four parameters were analyzed for each assay: (1) the first choice made by the millipede, (2) the time spent at each cup, (3) the total time spent per arm per replicate, and (4) the number of choices for each arm per replicate. We chose to record a choice only when a millipede reached the top of the arm, touched the cup, and we measured the time from when it touched the cup to when it turned entirely away from the cup. These parameters were chosen because of the variability in walking speed among individuals.

### Analysis of Differences in Chemical Secretions between Male and Female Millipedes

Individual millipedes were collected and extracted as described above. An aliquot (5 μL) of each extract was analyzed using the general LR-LCMS and GCMS methods. For relative quantification of **5**–**8**, raw LCMS files were converted to mzML using MSConvert (v. 3.0.20337) and analyzed in Mzmine 4 (v4.4.3). Peak area detection was performed using a protocol as defined in Banks 2025.^28^ The percentage was calculated based on the total peak area for each species.

### Statistical Analyses

For all experiments, the significance threshold was set at α = 0.05. All analyses were performed using GraphPad Prism (v10.6.0).

#### Males vs Females

Differences in deoxybuzonamine isomers (**5a** and **5b**) among males, females, and brood males were evaluated using Fisher’s exact test for each pair. Metabolite amounts were assessed for differences in peak area between sexes using the Kruskal–Wallis test. A Fisher’s exact test was used to determine differences between isomer proportions in males, females, and brood males.

#### Y-Tube Choice Assay

First-choice data were analyzed using a 2-sided and a right-tailed binomial test, comparing the experimental data to the null hypothesis of 0.5. One-way ANOVA, followed by Dunnett’s multiple comparisons, was used to evaluate differences between blanks and all effect experiments. To analyze each experiment between the effect arm and the empty arm, a Welch’s *t* test was done for time spent per choice, and a paired *t* test was used for total time spent and number of approaches.

### Molecular Phylogeny of Brachycybe Millipedes

DNA was extracted using the Qiagen Genomic Tips DNA extraction kit from millipedes (three to five body rings). The manufacturer’s protocol was followed, with the addition of 50 μL of Proteinase K halfway through the incubation. The concentration of the obtained DNA was quantified using a Nanodrop UV/vis spectrophotometer and sequenced on an Illumina NextSeq Raw sequencing reads were demultiplexed, adapters were removed, and reads were trimmed with Trimmomatic.^36^ Targeted gene assembly was accomplished with aTRAM 2.0 ^37^ using a set of 312 millipede gene orthologs according to Marek et al.^38^ Assembled genes were translated into amino acid sequences and aligned using Geneious and MAFFT,^39^ and a phylogenetic tree was estimated with IQTREE 2.^40^

## Supplementary Material



## Data Availability

The data resulting from this study are available in the published article and its Supporting Information. The original NMR data (FIDs) are available at NP-MRD under accession numbers NP0353865, NP0353866, and NP0353867 (https://np-mrd.org/). MassIVE Repository under accession number MSV000102232 (DOI: 10.25345/C5W951297). Raw reads for the newly sequenced whole genomes are deposited in NCBI under Bioproject PRJNA1445324, with accession numbers ranging from SAMN56786260 to SAMN56786264.

## Notes

Biological and ecological assays, mass spectrometry data, NMR spectra, and NMR data tables (PDF)
