# Supplementary material for: Evaluation of the Alkaloid Secretions within the Millipede Brachycybe and Their Pheromone Properties
Source: J Nat Prod. 2026 Jul 21;89(8):2322–31. doi: 10.1021/acs.jnatprod.6c00556 (PMC13532187; doi:10.1021/acs.jnatprod.6c00556)
Supplement: Supplementary file 1 [file np6c00556_si_001.pdf]

Supplemental Material

Evaluation of the Alkaloid Secretions within the Millipede *Brachycybe* and Their Pheromone Properties

Arden Hatch,[1] Anne C. Jones,[2,#] Luisa F. Vasquez-Valverde,[2,#] Paige Banks,[1,#] Isabel Gooding,[1] Lisa Markoff,[1] Paul E. Marek,[2] Emily Mevers[1,*]

[1]Department of Chemistry, Virginia Tech, Blacksburg, VA 24061, [2]Department of Entomology, Virginia Tech, Blacksburg, VA 24061

[#]Authors contributed equally

[1]Department of Chemistry, Virginia Tech, Blacksburg, VA 24061

[2]Department of Entomology, Virginia Tech, Blacksburg, VA 24061

*Address correspondence to emevers@vt.edu

Table of Contents

Supplemental Tables

Table S1: Information on Colobognatha millipede collection……………………………………………………..…4
Table S2. Percentages of compound prevalence. The most abundant defense secretion is bolded in each species……….7
Table S3. NMR Spectroscopy Data (^{1}H 600 MHz, d_6-DMSO, 50 ºC) for hydrogosodesmine acid (**6**)..............................7
Table S4. NMR Spectroscopy Data (^{1}H 600 MHz, d_6-DMSO, 50ºC) for homo-hydrogosodesmine acid (**7**).................... 8
Table S5. ^{13}C NMR chemical shift comparison of the bicyclic moiety in **6** with synthetic analogs………………………9
Table S6. ^{13}C NMR chemical shift comparison of the bicyclic moiety in **7** with synthetic analogs…………………….. 10
Table S7: Two-sided binomial test for first choice statistics for each Y-tube experiment………………………………..10
Table S8. Dunnett's multiple comparisons test for a number of approaches…………………………………………… 11
Table S9. Dunnett's multiple-comparison test for time spent per choice relative to the blank………………………….. 11
Table S10. Welch's t-test for time spent per choice between experiment arms…………………………………………..12
Table S11. Paired t-test for the total time spent between the effect and the empty arm…………………………………12
Table S12. Paired t-test for the number of approaches between the effect and the empty arms within experiments……12
Table S13. Count of individuals from males, females, and brood males that produce **5a** or **5b**, or **5a** and **5b**…………..13
Table S14. Count of individuals from males, females, and brood males that produce **5a** and **5b**………………………..13
Table S15: Brood male and associated egg clutch **5a** and **5b** production……………………………………………….. 13

Supplementary Figures

Figure S1. LR-LCMS chromatogram of *B. rosea* crude extract...14
Figure S2. LR-LCMS chromatogram of *B. lecontii* crude extract 14
Figure S3. LR-LCMS chromatogram of *B. petasata* crude extract...15
Figure S4. LR-LCMS chromatogram of *B. producta* (coastal) crude extract.. 15
Figure S5. LR-LCMS chromatogram of *B. producta* (mountainous) crude extract..16
Figure S6. LR-LCMS chromatogram of *B. picta* crude extract.. 16
Figure S7. LR-LCMS chromatogram of *B. nodulosa* crude extract..17
Figure S8. GCMS chromatogram of *B. picta* crude extract……………………………………………………………... 17
Figure S9. GCMS chromatogram of *B. nodulosa* crude extract………………………………………………………….18
Figure S10. GCMS chromatogram of *B. rosea* crude extract……………………………………………………………18
Figure S11. GCMS chromatogram of *B. lecontii* crude extract………………………………………………………… 19
Figure S12. GCMS chromatogram of *B. petesata* crude extract…………………………………………………………19
Figure S13. GCMS chromatogram of *B. producta* crude extract……………………………………………………….. 20
Figure S14. GCMS comparison of deoxybuzonamine standards with a representative extract of *B. producta*………… 20
Figure S15. Representative GCMS spectra for compounds **1**–**5**……………………………………………………………21
Figure S16. MSMS spectra of **6** acquired on a Thermo Exploris Orbitrap…………………………………………………22
Figure S17. MSMS spectra of **7** acquired on a Thermo Exploris Orbitrap…………………………………………………22
Figure S18. MSMS spectra of **8** acquired on a Thermo Exploris Orbitrap…………………………………………………23
Figure S19. ^{1}H NMR spectrum of hydrogosodesmine acid (**6**) in d_6-DMSO (^{1}H 600 MHz)... 23
Figure S20. gHSQC NMR spectrum of hydrogosodesmine acid (**6**) in d_6-DMSO (^{1}H 600 MHz; ^{13}C 150 MHz).............24
Figure S21. dqfCOSY NMR spectrum of hydrogosodesmine acid (**6**) in d_6-DMSO (^{1}H 600 MHz)...............................24
Figure S22. H2BC NMR spectrum of hydrogosodesmine acid (**6**) in d_6-DMSO (^{1}H 600 MHz; ^{13}C 150 MHz)...............25
Figure S23. HMBC NMR spectrum of hydrogosodesmine acid (**6**) in d_6-DMSO (^{1}H 600 MHz; ^{13}C 150 MHz)............ 25
Figure S24. easyROESY NMR spectrum of hydrogosodesmine acid (**6**) in d_6-DMSO (^{1}H 600 MHz)............................26
Figure S25. 1D NOE NMR spectra of hydrogosodesmine acid (**6**) in d_6-DMSO (^{1}H 600 MHz)....................................26
Figure S26. ^{1}H NMR spectrum of homo-hydrogosodesmine acid (**7**) in d_6-DMSO (^{1}H 600 MHz)................................27
Figure S27. gHSQC NMR spectrum of homo-hydrogosodesmine acid (**7**) in d_6-DMSO (^{1}H 600 MHz; ^{13}C 150 MHz)..27
Figure S28. dqfCOSY NMR spectrum of homo-hydrogosodesmine acid (**7**) in d_6-DMSO (^{1}H 600 MHz)......................28
Figure S29. H2BC NMR spectrum of homo-hydrogosodesmine acid (**7**) in d_6-DMSO (^{1}H 600 MHz; ^{13}C 150 MHz).....28
Figure S30. HMBC NMR spectrum of homo-hydrogosodesmine acid (**7**) in d_6-DMSO (^{1}H 600 MHz; ^{13}C 150 MHz)...29
Figure S31. 1D NOE NMR spectra of homo-hydrogosodesmine acid (**7**) in d_6-DMSO (^{1}H 600 MHz).......................... 29
Figure S32. ^{1}H NMR and ^{13}C NMR chemical shifts for **8**, with associated 2D NMR correlations …………………….. 30

Figure S33. Y-tube choice assay setup………………………………………….……………………………….. 30
Figure S34. Violin plot for the number of approaches per arm when compared to the blank……………………………31
Figure S35. Violin plot for total time spent between effect and empty arms within experiments. ………………………32
Figure S36. Violin plot for differences between effect and empty arms within experiments. ………………………….. 32
Figure S37. Violin plot for total time spent between the effect and the empty arm within experiments………………... 33
Figure S38. Violin plot for the number of approaches between the effect and the empty arm within experiments…….. 34
Figure S39. LR-LCMS chromatogram of *B. cerviculatum* crude extract (x-axis is in minutes)..................................... 34
Figure S40. LR-LCMS concentration curve of **2** and **6**……………………………………… 35

Table S1: Information on Colobognatha millipede collection

Species	Date	Location	County	Description	state	latitude	longitude	elevation
B. producta	5/27/25	Sly Park	Eldorado County	Mormon immigrant trail	CA	38.711258	-120.55891	1095 m
B. producta	2/9/25	Sly Park	Eldorado County	Mormon immigrant trail	CA	38.711258	-120.55891	1095 m
B. producta	12/13/24	Sly Park	Eldorado County	Mormon immigrant trail	CA	38.711258	-120.55891	1095 m
B. producta	12/3/24	Sly Park	Eldorado County	Mormon immigrant trail	CA	38.711258	-120.55891	1095 m
B. producta	11/5/24	Sly Park	Eldorado County	Mormon immigrant trail	CA	38.711258	-120.55891	1095 m
B. producta	10/13/24	Sly Park	Eldorado County	Mormon immigrant trail	CA	38.711258	-120.55891	1095 m
B. producta	8/27/24	Sly Park	Eldorado County	Mormon immigrant trail	CA	38.711258	-120.55891	1095 m
B. producta	7/22/24	Sly Park	Eldorado County	Mormon immigrant trail	CA	38.711258	-120.55891	1095 m
B. producta	5/10/24	Sly Park	Eldorado County	Mormon immigrant trail	CA	38.711258	-120.55891	1095 m
B. producta	4/7/24	Sly Park	Eldorado County	Mormon immigrant trail	CA	38.711258	-120.55891	1095 m
B. producta	11/25/23	Sly Park	Eldorado County	Mormon immigrant trail	CA	38.711258	-120.55891	1095 m
B. producta	4/26/25	Samuel P. Taylor State Park	Marin County	Marin Trail	CA	38.01881	-122.72972	80 m
B. producta	4/26/25	Samuel P. Taylor State Park	Marin County	Marin Trail	CA	38.01881	-122.72972	80 m
B. producta	4/23/25	Samuel P. Taylor State Park	Marin County	Marin Trail	CA	38.01881	-122.72972	80 m
B. producta	4/26/25	Samuel P. Taylor State Park	Marin County	Marin Trail	CA	38.01881	-122.72972	80 m
B. producta	4/26/25	Samuel P. Taylor State Park	Marin County	Marin Trail	CA	38.01881	-122.72972	80 m

Table S1: Information on Colobognatha millipede collection

B. rosea	5/27/25	Sly Park	Eldorado County	Mormon immigrant trail	CA	38.711258	-120.55891	1095 m
B. rosea	2/9/25	Sly Park	Eldorado County	Mormon immigrant trail	CA	38.711258	-120.55891	1095 m
B. rosea	12/13/24	Sly Park	Eldorado County	Mormon immigrant trail	CA	38.711258	-120.55891	1095 m
B. rosea	12/3/24	Sly Park	Eldorado County	Mormon immigrant trail	CA	38.711258	-120.55891	1095 m
B. rosea	11/5/24	Sly Park	Eldorado County	Mormon immigrant trail	CA	38.711258	-120.55891	1095 m
B. rosea	10/13/24	Sly Park	Eldorado County	Mormon immigrant trail	CA	38.711258	-120.55891	1095 m
B. rosea	8/27/24	Sly Park	Eldorado County	Mormon immigrant trail	CA	38.711258	-120.55891	1095 m
B. rosea	7/22/24	Sly Park	Eldorado County	Mormon immigrant trail	CA	38.711258	-120.55891	1095 m
B. rosea	5/10/24	Sly Park	Eldorado County	Mormon immigrant trail	CA	38.711258	-120.55891	1095 m
B. rosea	4/7/24	Sly Park	Eldorado County	Mormon immigrant trail	CA	38.711258	-120.55891	1095 m
B. rosea	11/25/23	Sly Park	Eldorado County	Mormon immigrant trail	CA	38.711258	-120.55891	1095 m
B. picta	8/3/24	Tamalpais State Park	Marin County	Bootjack trail	CA	37.90756	-122.59767	314 m
B. picta	8/2/24	Samuel P. Taylor State Park	Marin County	Pioneer Tree Trail	CA	38.016182	-122.73204	130 m
B. picta	8/5/24	Samuel P. Taylor State Park	Marin County	Pioneer Tree Trail	CA	38.016182	-122.73204	130 m
B. picta	8/4/24	Fort Ross State Historic Park	Sonoma County	Stanley S. Spyra Memorial Grove	CA	38.52408	-123.24156	139 m
B. picta	8/4/24	Salt Point State Park	Sonoma County	Salt Point Trail	CA	38.5839815	-123.32405	163 m
B. picta	3/23/25	Sonoma Coast State Park	Sonoma County	Pomo Canyon Trail	CA	38.424507	-123.09786	149 m

Table S1: Information on Colobognatha millipede collection

B. picta	4/24/25	Fort Ross State Historic Park	Marin County	Stanley S. Spyra Memorial Grove	CA	38.52408	-123.24156	139 m
B. picta	4/22/25	Samuel P Taylor State Park	Marin County	Pioneer Tree Trail	CA	38.016182	-122.73204	130 m
B. picta	4/23/25	Tamalpais State Park	Marin County	Bootjack trail	CA	37.90756	-122.59767	314 m
B. picta	4/23/25	Tamalpais State Park	Marin County	Steep Ravine Trail	CA	37.89699	-122.6125	325 m
B. picta	4/26/25	Samuel P Taylor State Park	Marin County	Cross Marin Trail	CA	38.02533	-122.73834	83 m
B. picta	4/26/25	Samuel P Taylor State Park	Marin County	Pioneer tree trail	CA	38.016182	-122.73204	130 m
B. lecontii	6/28/24	Moore Cove	Transylvania County	Moore Cove	NC	35.305466	-82.774664	740 m
B. lecontii	6/29/24	Harmon Den	Hayward County	Harmon Den	NC	35.75208	-83.00308	745 m
B. lecontii	6/15/25	Reliance	Polk County	Clemmer Trailhead	TN	35.115006	-84.578759	292 m
B. lecontii	7/3/25	Harmon Den	Hayward County	Harmon Den	NC	35.75208	-83.00308	745 m
B. lecontii	7/4/25	Moore Cove	Transylvania County	Moore Cove	NC	35.305466	-82.774664	740 m
B. lecontii	7/5/25	Harmon Den	Hayward County	Harmon Den	NC	35.75208	-83.00308	745 m
B. lecontii	7/5/25	Mill Creek Rd	Cooke County	Mill Creek Rd	TN	35.83301	-83.14033	490 m
B. lecontii	7/5/25	Draper Outlook	Pulaski County	Mystic Biscuit Trail	VA	37.02221	-80.78045	773 m
B. lecontii	7/19/24	Draper Outlook	Pulaski County	Mystic Biscuit Trail	VA	37.02221	-80.78045	773 m
B. petasata	6/29/24	Harmon Den	Hayward County	Harmon Den	NC	35.75208	-83.00308	747 m
B. petasata	6/15/25	Reliance	Polk County	Clemmer Trailhead	TN	35.115006	-84.578759	283 m
B. nodulosa	5/30/19	Japan, Nagasaki, Unzen, Mount Unzen, south slope near road (#57), Obamacho Minamikisashi				32.71194	130.216557	225 m
B. cerviculatum	5/27/25	Sly Park	Eldorado County	Mormon Immigrant Trail	CA	38.711258	-120.55891	1095 m
B. cerviculatum	2/9/25	Sly Park	Eldorado County	Mormon Immigrant Trail	CA	38.711258	-120.55891	1095 m

Table S2. Percentages of compound prevalence. The most abundant defense secretion is highlighted in bold for each species.

	**(1)**	**(2)**	**(3)**	**(4)**	**(5)**	**(6)**	**(7)**	**(8)**
B. picta	ND	ND	< 1	< 1	**69**	29	1	ND
B. petasata	16	ND	ND	**78**	ND	3	3	ND
B. producta (coastal)	1	35	1	**40**	ND	15	13	ND
B. producta (mountainous)	ND	**87**	< 1	4	ND	8	< 1	ND
B. rosea	ND	ND	< 1	4	**87**	8	< 1	< 1
B. lecontii	ND	ND	ND	ND	**85**	13	1.29	< 1
B. nodulosa	**72**	ND	< 1	< 1	27	< 1	< 1	< 1

Note: Response factors in **Figure S34** were not taken into considerations

Table S3. NMR Spectroscopy Data (^{1}H 600 MHz, d_6-DMSO, 50 ºC) for hydrogosodesmine acid (**6**)

Position	δ_C, type[a]	δ_H (J in Hz)	H2BC	HMBC	COSY	ROESY
1a	31.1, CH_2	1.64, m	2		1b	
1b		1.14, m	2, 8	2, 8	1a, 2a, 2b	
2a	51.2, CH_2	3.01, m	1	5, 6	1b, 2b	
2b		1.97, m	1	3	1b, 2a	
3a	52.7, CH_2	2.96, m	4	8	3b, 4, 5a	
3b		2.04, m	4	2, 4	3a	
4	20.4, CH_2	1.63, m	3, 5		3a	5b
5a	29.6, CH_2	1.79, m	4, 6		3a, 5b	
5b		1.27, m	4, 6	4, 6	5a	4, 7a
6	63.4, CH	1.85, m	5, 7	5	7b	
7a	36.3, CH_2	1.81, m	6, 8	1	7b	5b
7b		0.83, m	6, 8	6, 8	6, 7a, 8	
8	35.1, CH	1.28, m	7	7	7b	
9	34.8, CH_2	1.33, m	10	8, 10, 11	10	10, 11
10	25.2, CH_2	2.16, m	9, 11	9, 11, 12	9, 11, 14	9, 11, 14
11	140.6, CH	6.60, t (6.8)	10	9, 10, 12, 13, 14	10, 14	9, 10
12	128.8, C					
13	169.7, C					
14	12.0, CH_3	1.72, s		11, 12, 13	10, 11	10

[a]Interpreted from gHSQC and HMBC

Table S4. NMR Spectroscopy Data (^{1}H 600 MHz, DMSO, 50ºC) for homo-hydrogosodesmine acid (**7**)

Position	δ_C, type[a]	δ_H (J in Hz)	H2BC	HMBC	COSY
1a	31.2, CH_2	1.61, m	2, 9	9	
1b		1.13, m	2, 9	9	
2a	55.3, CH_2	2.73, m	2	1, 9	
2b		1.95, m	2	1, 7	
3a	55.1, CH_2	2.75, m	4	7, 4	
3b		1.92, m	4	7	
4a	25.1, CH_2	1.55, m	5	6	
4b		1.43, m	3, 5		5b
5a	23.6, CH_2	1.63, m	6		6b
5b		1.16, m	4	4	4b
6a	32.4, CH_2	1.50, m	5, 7	5, 7, 8	
6b		1.13, m	7	5, 7	5a
7	61.3, CH	1.73, m	6, 8	6	
8a	38.4, CH_2	1.56, m	7, 9	7	
8b		0.85, m	7, 9	7, 9	9
9	34.8, CH	1.26, m	1, 8	8, 10	8b
10	34.6, CH_2	1.28, m	9, 11	9, 11, 12	11
11	25.0, CH_2	2.13, m	10, 12	10, 12, 13	10, 12
12	140.8, CH	6.60, t (6.7)	11	10, 11, 13, 14, 15	11
13	127.9, C				
14	168.4, C				
15	12.0, CH_3	1.72, s		12, 13, 14	

[a]Interpreted from gHSQC and HMBC

Table S5. ^{13}C NMR chemical shift comparison of the bicyclic moiety in **6** with synthetic analogs

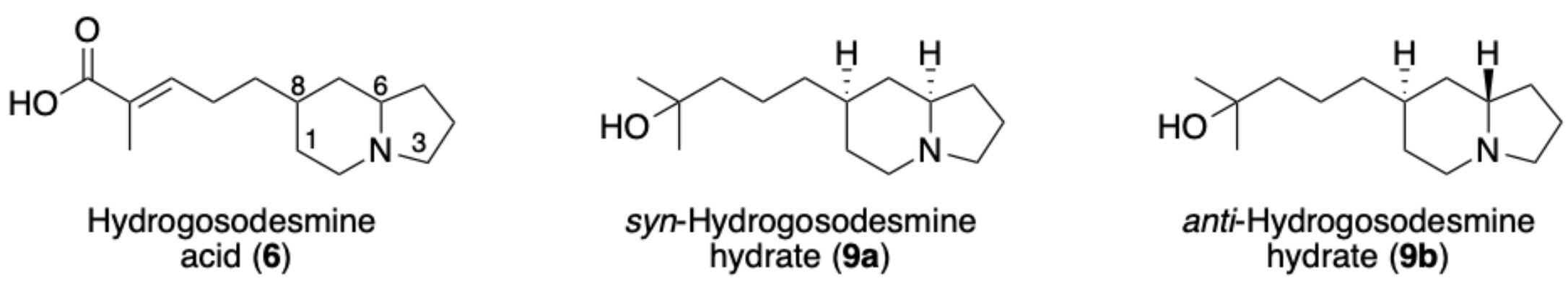

Position	6	*syn*-9a	*anti*-9b	$\Delta_{6/9a}$	$\Delta_{6/9b}$
1	31.1	31.4	28.6	0.3	2.5
2	51.2	51.3	46.8	0.1	4.4
3	52.7	52.7	52.8	0	0.1
4	20.4	20.4	19.9	0	0.5
5	29.6	29.6	28.6	0	1
6	63.4	63.4	58	0	5.4
7	36.3	36.5	33.2	0.2	3.1
8	35.1	35.4	30.1	0.3	5
9	31.1	31.4	28.6	0.3	2.5
**Average**				**0.11**	**2.75**
**Stdev**				**0.14**	**2.08**

Table S6. ^{13}C NMR chemical shift comparison of the bicyclic moiety in **7** with synthetic analogs

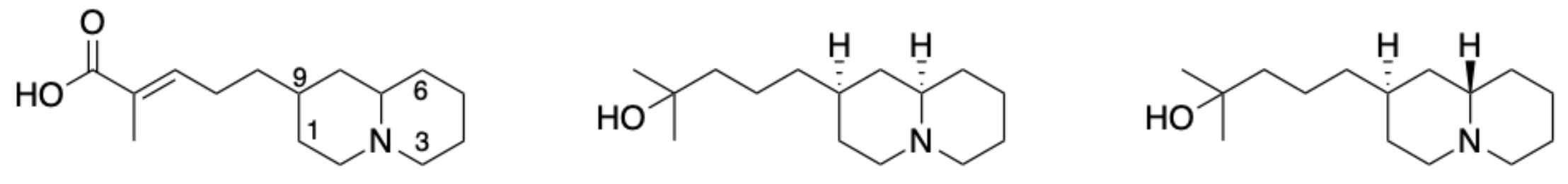

Homo-hydrogosodesmine acid (**7**) — *syn*-Homo-hydrogosodesmine hydrate (**10a**) — *anti*-Homo-hydrogosodesmine hydrate (**10b**)

**Position**	**7**	*syn*-**10a**	*anti*-**10b**	$\Delta_{7/10a}$	$\Delta_{7/10b}$
1	31.2	31.4	26.9	0.2	4.3
2	55.3	51.3	48.3	4.0	7.0
3	55.1	52.7	53.5	2.4	1.6
4	25.1	20.4	22.4	4.7	2.7
5	23.6	23.5	23.4	0.1	0.2
6	32.4	30.7	30.8	1.7	1.6
7	61.3	61.6	56.5	0.3	4.8
8	38.4	37.4	34.4	1.0	4.0
9	34.8	34.1	29.3	0.7	5.5
**Average**				**1.68**	**3.52**
**Stdev**				**1.70**	**2.17**

Table S7: Two-sided binomial test for first choice statistics for each Y-tube experiment

**Two-sided binomial test**	**p-value**	**Statistic**	**95% conidence interval**	**n**
B. producta	0.0428	0.7	[0.506,0.8527]	30
B. rosea	0.36	0.4	[0.2266, 0.594]	30
B. producta – B. cerviculatum	0.0161	0.7333	[0.5411,0.8772]	30
B. rosea – B. cerviculatum	0.2005	0.6333	[0.4386,0.8007]	30
Box – *B. producta* individual	0.3438	0.7	[0.3475,0.9333]	10
Box – *B. producta* 5 individuals	0.3438	0.7	[0.3475,0.9333]	10
B. producta – 300 μg of crude extract	0.0428	0.7	[0.506,0.8527]	30
B. producta – 150 μg of **2**	0.0433	0.72	[0.5061,0.8793]	25
Blank – CH_3OH	0.7539	0.4	[0.1216,0.7376]	10
Blank	1	0.5	[0.1871, 0.8129]	10

Table S8. Dunnett's multiple comparisons test for a number of approaches.

Experiment	Mean diff.	95.00% CI of diff.	Below threshold?	Summary	Adjusted p-value
Blank vs. *B. producta* – *B. producta*	-1.933	-6.002 to 2.135	No	ns	0.5967
Blank vs. *B. producta* – *B. cerviculatum*	-2.033	-6.102 to 2.035	No	ns	0.5521
Blank vs. *B. rosea* – *B. rosea*	-6.533	-10.60 to -2.465	Yes	***	0.0004
Blank vs. *B. rosea* – *B. cerviculatum*	-7.000	-11.07 to -2.931	Yes	***	0.0001
Blank vs. Box – *B. producta* individual	-1.000	-5.983 to 3.983	No	ns	0.9809
Blank vs. Box – *B. producta* 5 individuals	2.300	-2.683 to 7.283	No	ns	0.6217
Blank vs. *B. producta* – 300 µg of crude extract	1.467	-0.4658 to 3.399	No	ns	0.1526
Blank vs. *B. producta* – 150 µg of **2**	2.04	0.05983 to 4.020	Yes	*	0.0428

ns = not significant, * = < 0.05, ** = < 0.01, *** = < 0.001

Table S9. Dunnett's multiple-comparison test for time spent per choice relative to the blank.

Experiment	Mean diff.	95.00% CI of diff.	Below threshold?	Summary	Adjusted p-value
Blank vs. *B. producta-B. producta*	-8.581	-22.82 to 5.654	No	ns	0.4679
Blank vs. *B. producta- B. cerviculatum* (empty)	1.873	-12.28 to 16.03	No	ns	> 0.9999
Blank vs. *B. rosea- B. rosea*	14.09	0.3287 to 27.85	Yes	*	0.042
Blank vs. *B. rosea- B. cerviculatum* (empty)	14.9	1.150 to 28.64	Yes	*	0.0266
Blank vs. Box *B. producta* individual	-4.636	-22.03 to 12.75	No	ns	0.9926
Blank vs. Box *B. producta* 5 individuals	-32.4	-51.39 to -13.41	Yes	****	< 0.0001
Blank CH_3OH vs. *B. producta* 300 µg of crude extract	-3	-15.84 to 9.837	No	ns	0.9197
Blank CH_3OH vs. *B. producta* - 150 µg of **2**	-7.309	-20.65 to 6.035	No	ns	0.4237

ns = not significant, * = < 0.05, ** = < 0.01, *** = < 0.001, **** = < 0.0001

Table S10. Welch's t-test for time spent per choice between experiment arms

Experiment	Difference between means	Summary	p-value
B. producta – B. producta	3.759 ± 5.196	ns	0.2349
B. producta – B. cerviculatum	-5.661 ± 3.501	ns	0.0532
B. rosea – B. rosea	-0.9587 ± 1.820	ns	0.2992
B. rosea – B. cerviculatum	3.123 ± 1.559	*	0.0227
Box – *B. producta* individual	-1.772 ± 5.349	ns	0.3704
Box – *B. producta* 5 individuals	-28.27 ± 12.73	*	0.0142
B. producta – 300 µg of crude extract	-12.70 ± 4.130	**	0.0011
B. producta – 150 µg of **2**	-10.33 ± 4.879	*	0.0174

ns = not significant, * = < 0.05, ** = < 0.01, *** = < 0.001, **** = < 0.0001

Table S11. Paired t-test for the total time spent between the effect and the empty arm

Experiment	Mean of the differences	Summary	P-value
B. producta – B. producta	28.9	ns	0.3125
B. producta – B. cerviculatum	-46.97	ns	0.1683
B. rosea – B. rosea	-19.47	ns	0.3147
B. rosea – B. cerviculatum	44.23	ns	0.1148
Box – *B. producta* individual	-17.9	ns	0.384
Box – *B. producta* 5 individuals	-182.4	ns	0.1132
B. producta – 300 µg of crude extract	-169.3	****	< 0.0001
B. producta – 150 µg of **2**	-97.56	*	0.0392

ns = not significant, * = < 0.05, ** = < 0.01, *** = < 0.001

Table S12. Paired t-test for the number of approaches between the effect and the empty arms within experiments.

Experiment	Mean of the differences	Summary	P-value
B. producta – B. producta	-0.37	ns	0.1544
B. producta – B. cerviculatum	0.2	ns	0.3527
B. rosea – B. rosea	-0.1	ns	0.4194
B. rosea – B. cerviculatum	-0.17	ns	0.3475
Box – *B. producta* individual	0	ns	0.5
Box – *B. producta* 5 individuals	0.5	ns	0.2645
B. producta – 300 µg of crude extract	-1.1	**	0.0055
B. producta – 150 µg of **2**	-0.2	ns	0.3641

ns = not significant, * = < 0.05, ** = < 0.01, *** = < 0.001

Table S13. Count of individuals from males, females, and brood males that produce **5a** or **5b**, or **5a** and **5b**.

**Sex**	**5a** or **5b**	**5a** and **5b**
Females	42	3
Males	23	4
Brood Males	11	8

Table S14. Count of individuals from males, females, and brood males that produce **5a** and **5b**.

**Sex**	**5a**	**5b**
Females	20	30
Males	2	24
Brood Males	7	8

Table S15: Brood male and associated egg clutch **5a** and **5b** production

**Brood Male**	**Isomer 5 of Brood Male**	**Isomer 5 of Egg Clutch**
1	**5b**	**5a**
2	**5b**	ND
3	**5b**	ND
4	**5b**	ND
5	**5b**	ND
6	**5b**	ND
7	**5b**	ND
8	**5b**	**5b**
9	**5a** + **5b**	ND
10	**5a** + **5b**	**5a**
11	**5a**	**5a** + **5b**
12	**5a** + **5b**	**5a**
13	**5a** + **5b**	**5b**
14	**5a**	**5a** + **5b**
15	**5a**	ND

ND = not detected

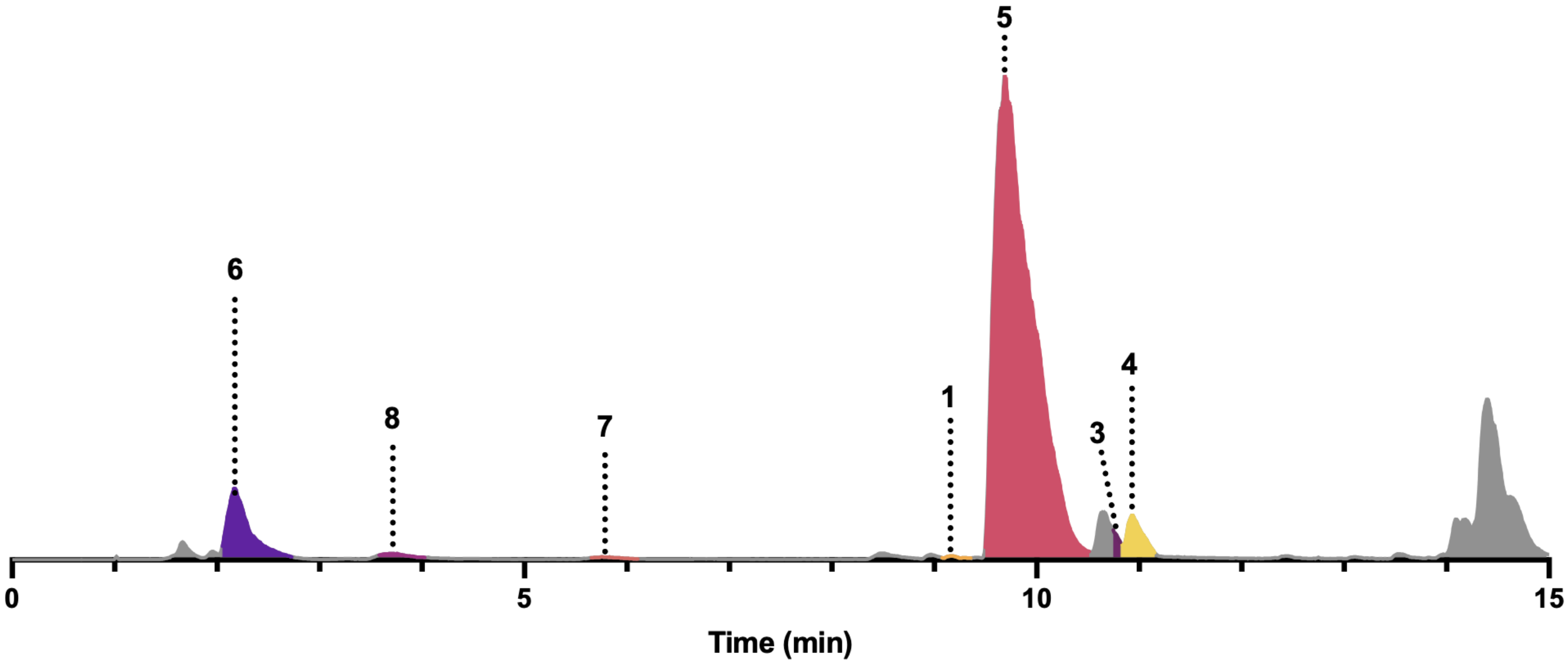

Figure S1. LR-LCMS base-peak chromatogram of *B. rosea* crude extract (x-axis in minutes). Peaks representing characterized alkaloids are labeled in the chromatogram with the peak representing hydrogosodesmine acid (**6**) in violet, buzonamine acid (**8**) in magenta, homo-hydrogosodesmine acid (**7**) in coral, gosodesmine (**1**) in orange, deoxybuzonamine isomers (**5a/5b**) in pink, homogosodesmine (**3**) in plum, and homo-hydrogosodesmine (**4**) in yellow. Gray peaks are currently uncharacterized.

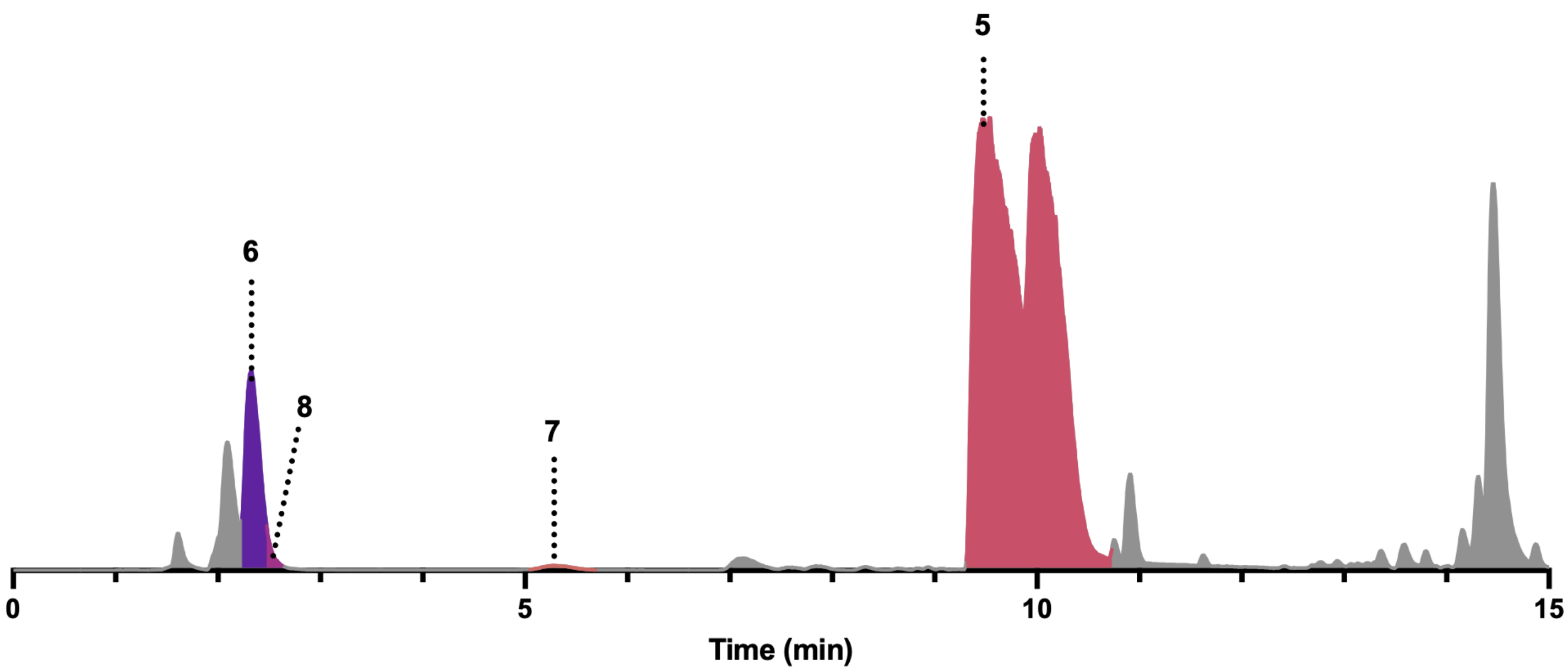

Figure S2. LR-LCMS base-peak chromatogram of *B. lecontii* crude extract (x-axis in minutes). Peaks representing characterized alkaloids are labeled in the chromatogram with the peak representing hydrogosodesmine acid (**6**) in violet, buzonamine acid (**8**) in magenta, homo-hydrogosodesmine acid (**7**) in coral, and deoxybuzonamine isomers (**5a/5b**) in pink. Gray peaks are currently uncharacterized.

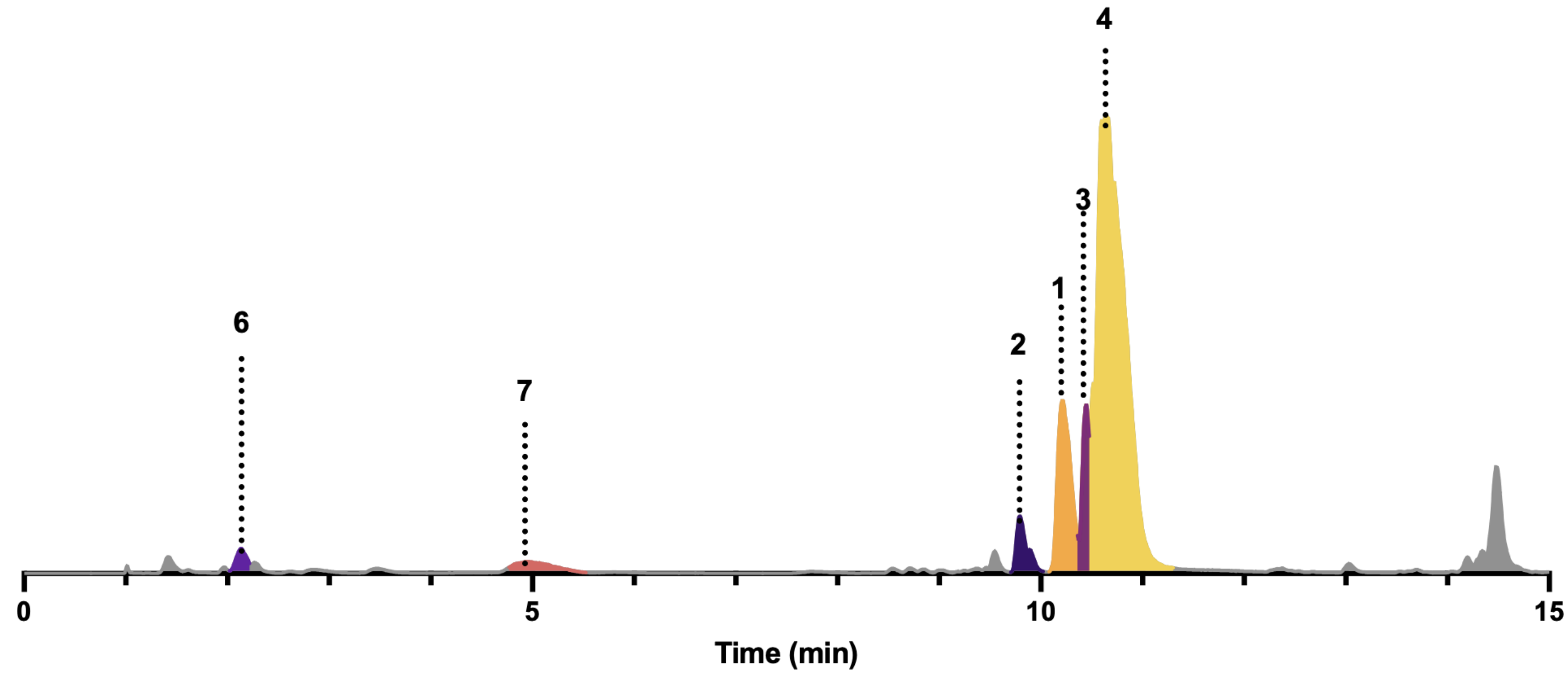

Figure S3. LR-LCMS base-peak chromatogram of *B. petasata* crude extract (x-axis in minutes). Peaks representing characterized alkaloids are labeled in the chromatogram with the peak representing hydrogosodesmine acid (**6**) in purple, homo-hydrogosodesmine acid (**7**) in coral, hydrogosodesmine (**2**) in dark blue, gosodesmine (**1**) in orange, homo-gosodesmine (**3**) in plum, and homo-hydrogosodesmine (**4**) in yellow. Gray peaks are currently uncharacterized.

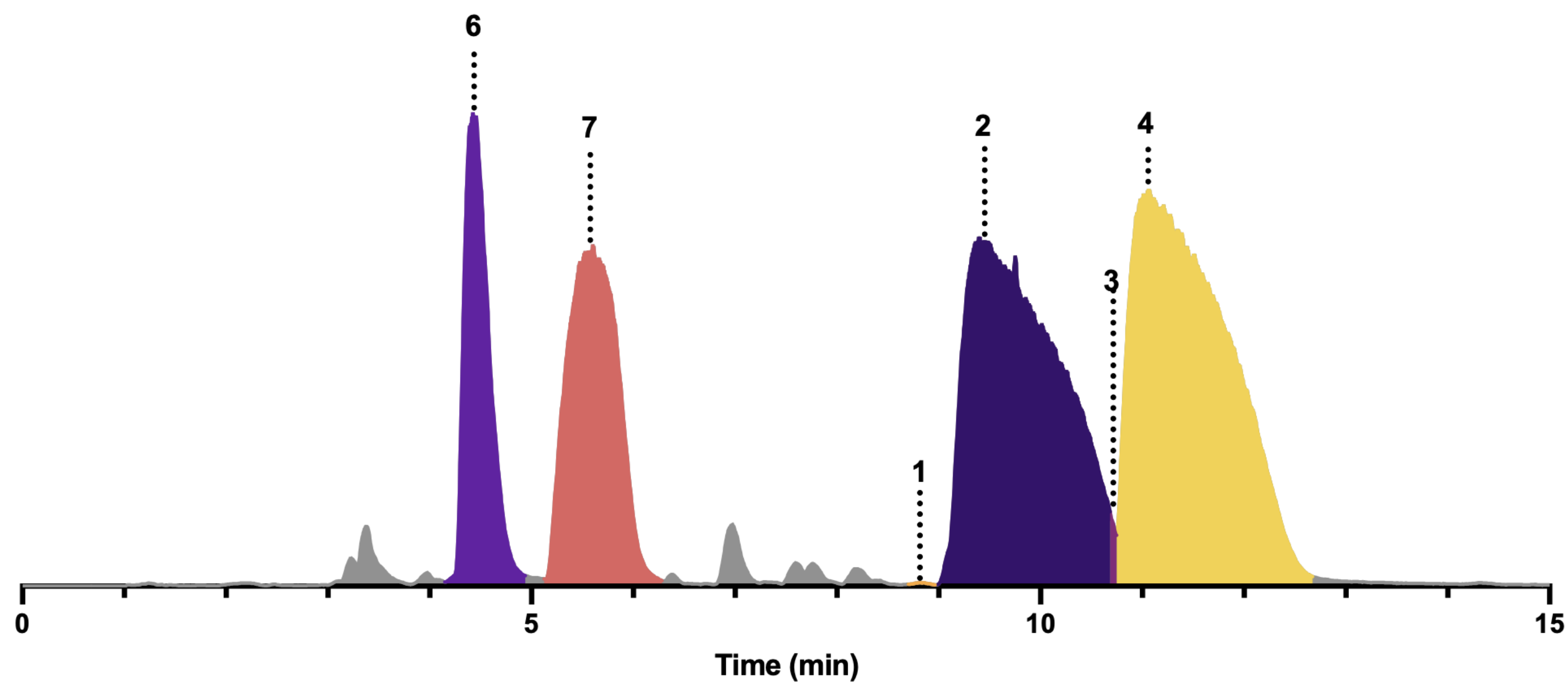

Figure S4. LR-LCMS base-peak chromatogram of *B. producta* (coastal) crude extract (x-axis in minutes). Peaks representing characterized alkaloids are labeled in the chromatogram with the peak representing hydrogosodesmine acid (**6**) in violet, homo-hydrogosodesmine acid (**7**) in magenta, gosodesmine (**1**) in orange, hydrogosodesmine (**2**) in dark blue, and homo-hydrogosodesmine (**4**) in yellow. Gray peaks are currently uncharacterized, and homo-gosdesmine (**3**) is beneath both **2** and **4**.

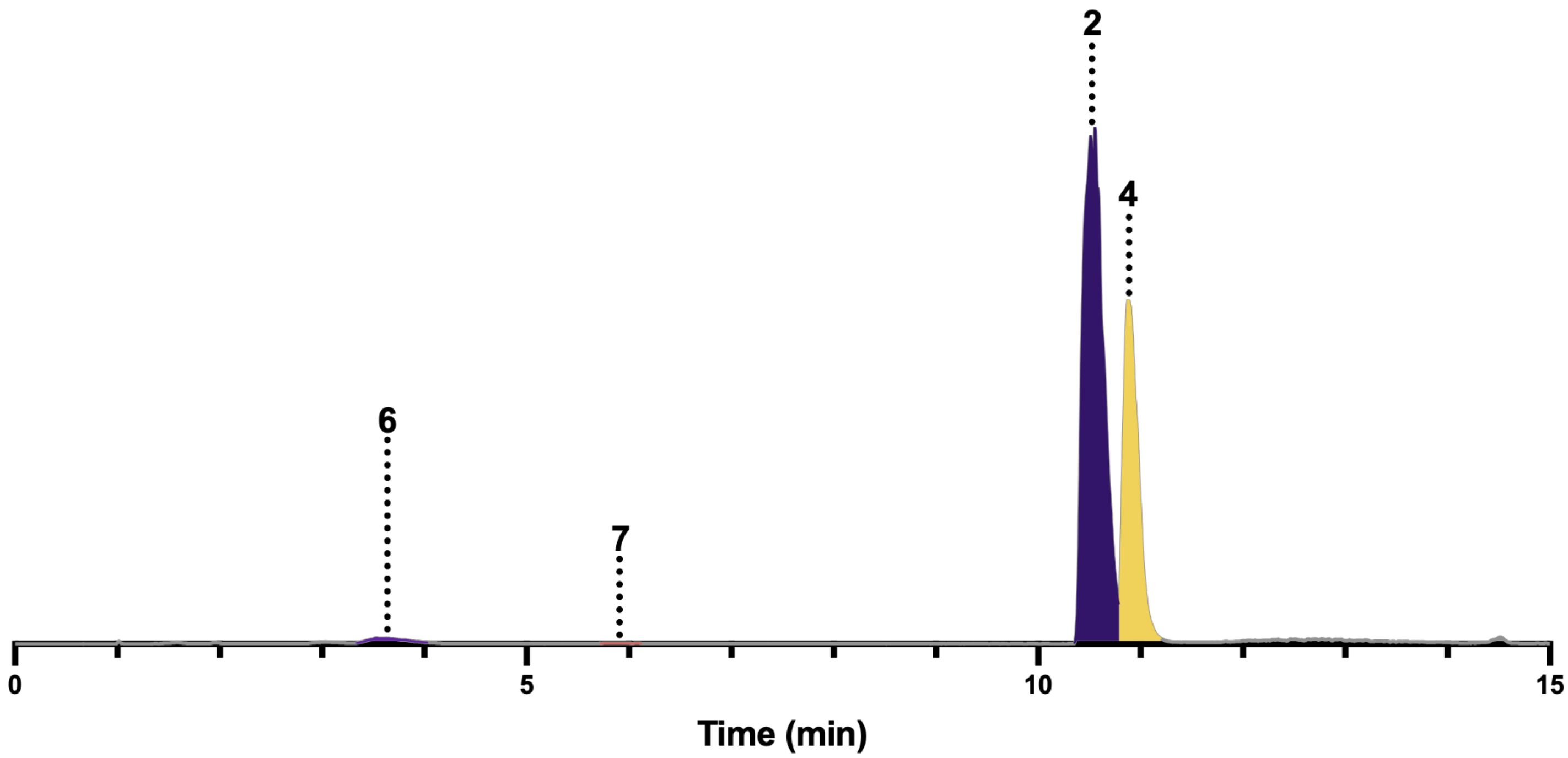

Figure S5. LR-LCMS base-peak chromatogram of *B. producta* (mountainous) crude extract (x-axis in minutes). Peaks representing characterized alkaloids are labeled in the chromatogram with the peak representing hydrogosodesmine acid (**6**) in violet, homo-hydrogosodesmine acid (**7**) in coral, hydrogosodesmine (**2**) in dark blue, and homo-hydrogosodesmine (**4**) in yellow.

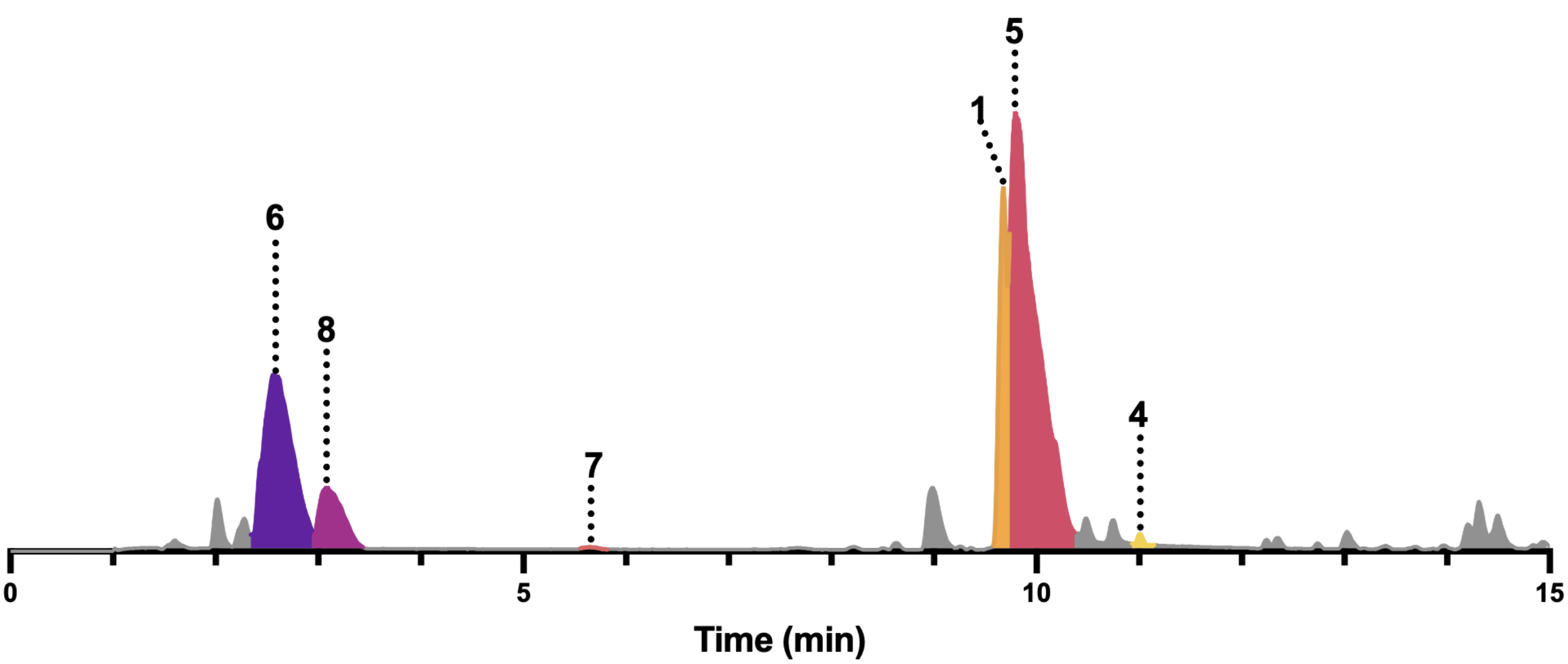

Figure S6. LR-LCMS base-peak chromatogram of *B. picta* crude extract (x-axis in minutes). Peaks representing characterized alkaloids are labeled in the chromatogram with the peak representing hydrogosodesmine acid (**6**) in violet, buzonamine acid (**8**) in magenta, homo-hydrogosodesmine acid (**7**) in coral, gosodesmine (**1**) in orange, deoxybuzonamine isomers (**5a/5b**) in pink, and homo-hydrogosodesmine (**4**) in yellow. Gray peaks are currently uncharacterized.

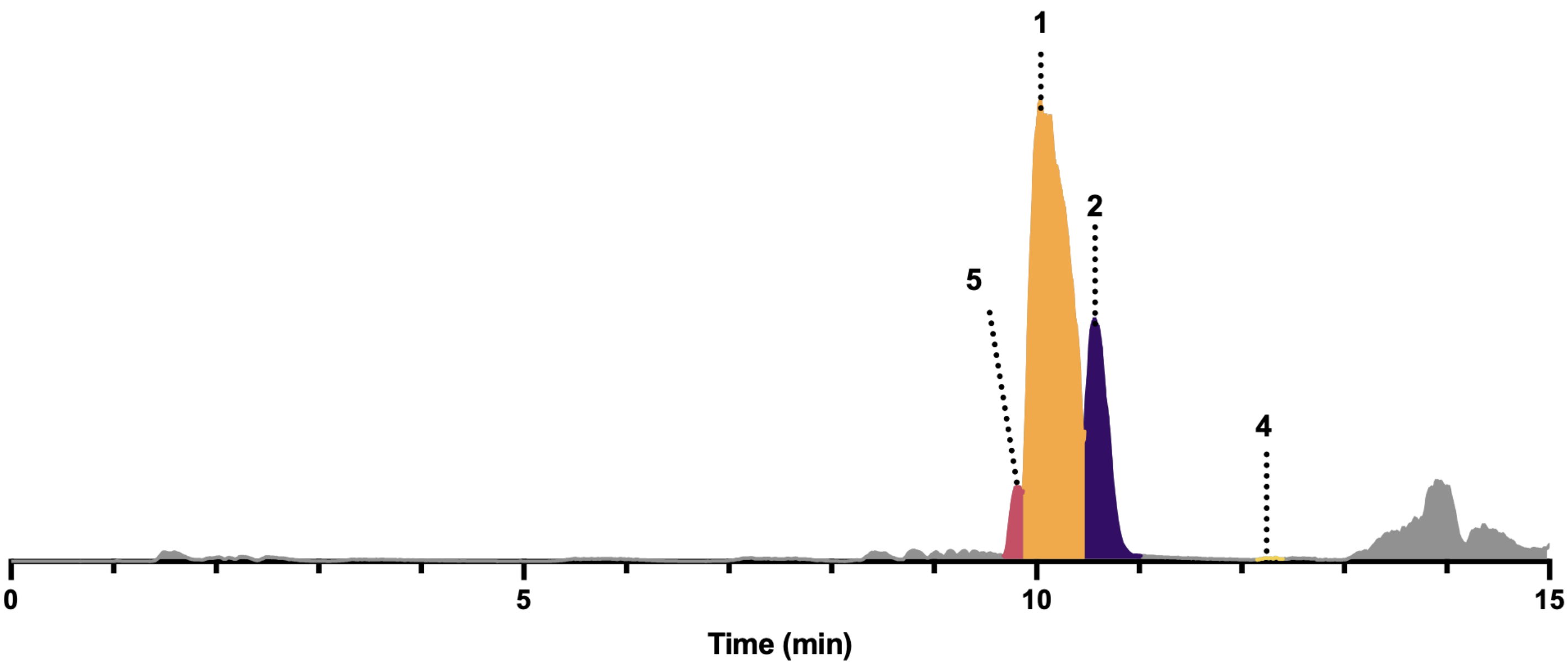

Figure S7. LR-LCMS base-peak chromatogram of *B. nodulosa* crude extract (x-axis in minutes). Peaks representing characterized alkaloids are labeled in the chromatogram with the peak representing deoxybuzonamine isomer (**5**) in pink, **g**osodesmine (**1**) in orange, hydrogosodesmine (**2**) in dark blue, and homo-hydrogosodesmine (**4**) in yellow. Gray peaks are currently uncharacterized.

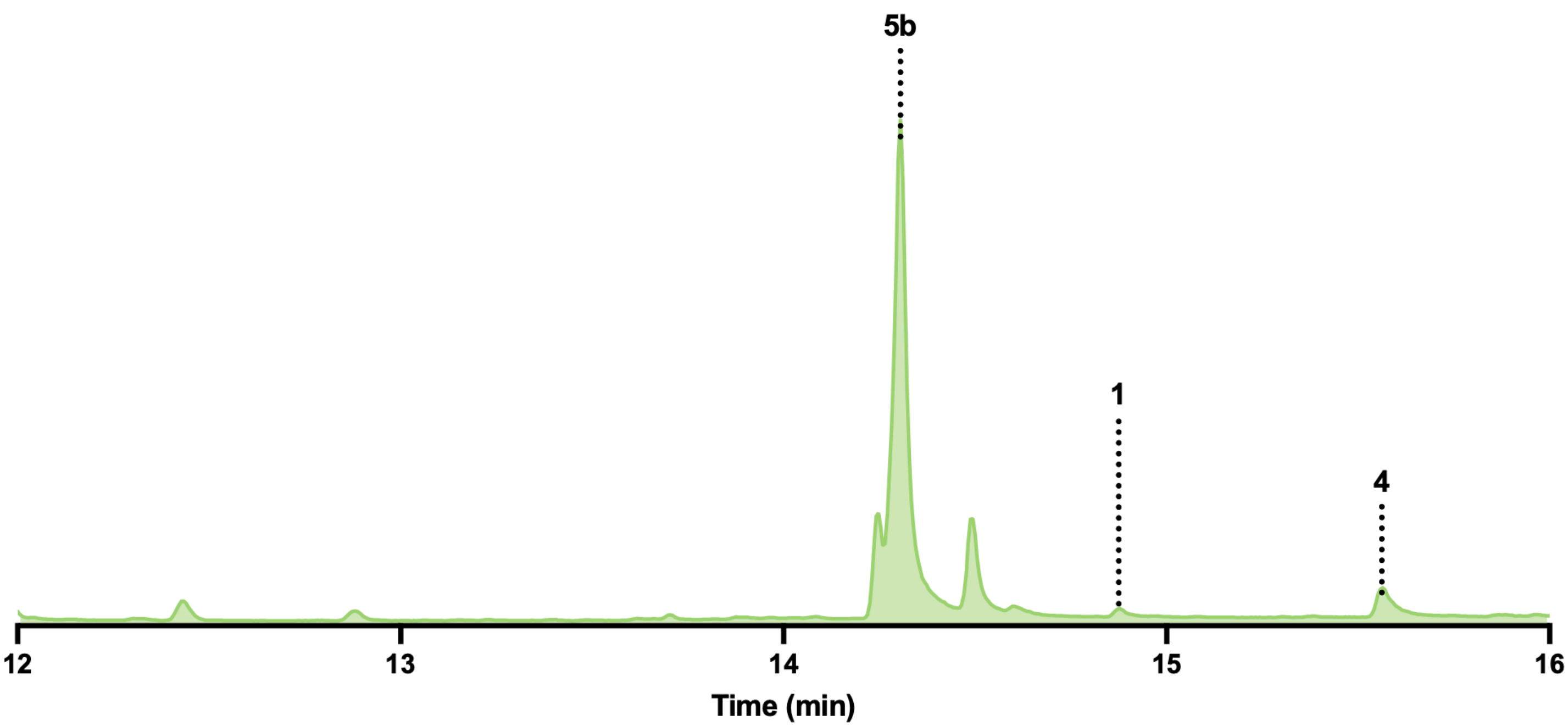

Figure S8. GCMS base-peak chromatogram of *B. picta* crude extract. Peaks corresponding to particular alkaloids are denoted with the compound number.

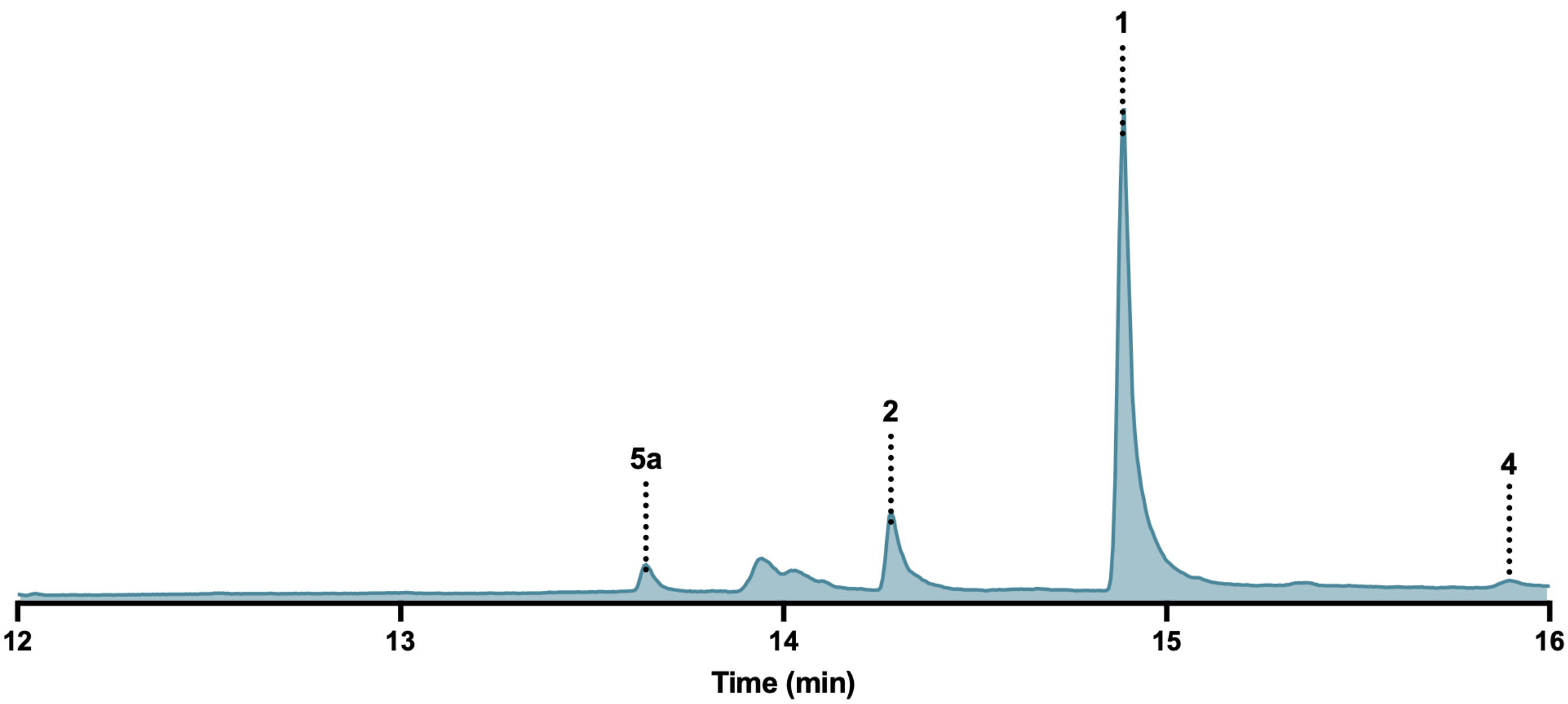

Figure S9. GCMS base-peak chromatogram of *B. nodulosa* crude extract. Peaks corresponding to particular alkaloids are denoted with the compound number.

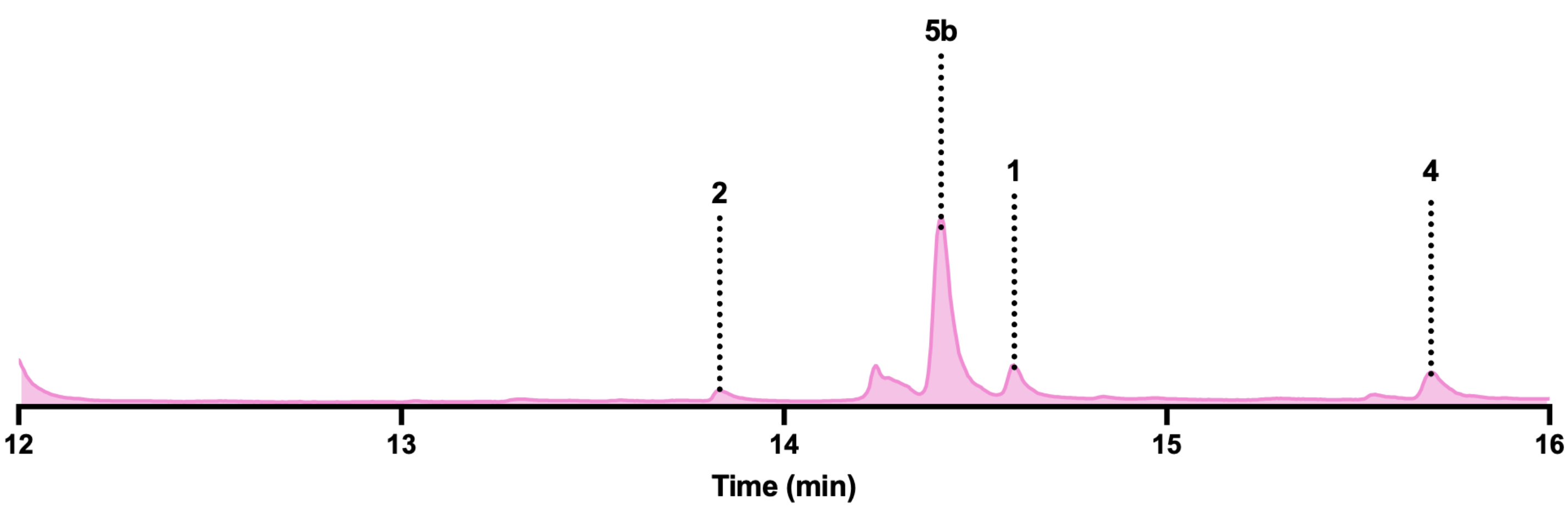

Figure S10. GCMS base-peak chromatogram of *B. rosea* crude extract. Peaks corresponding to particular alkaloids are denoted with the compound number.

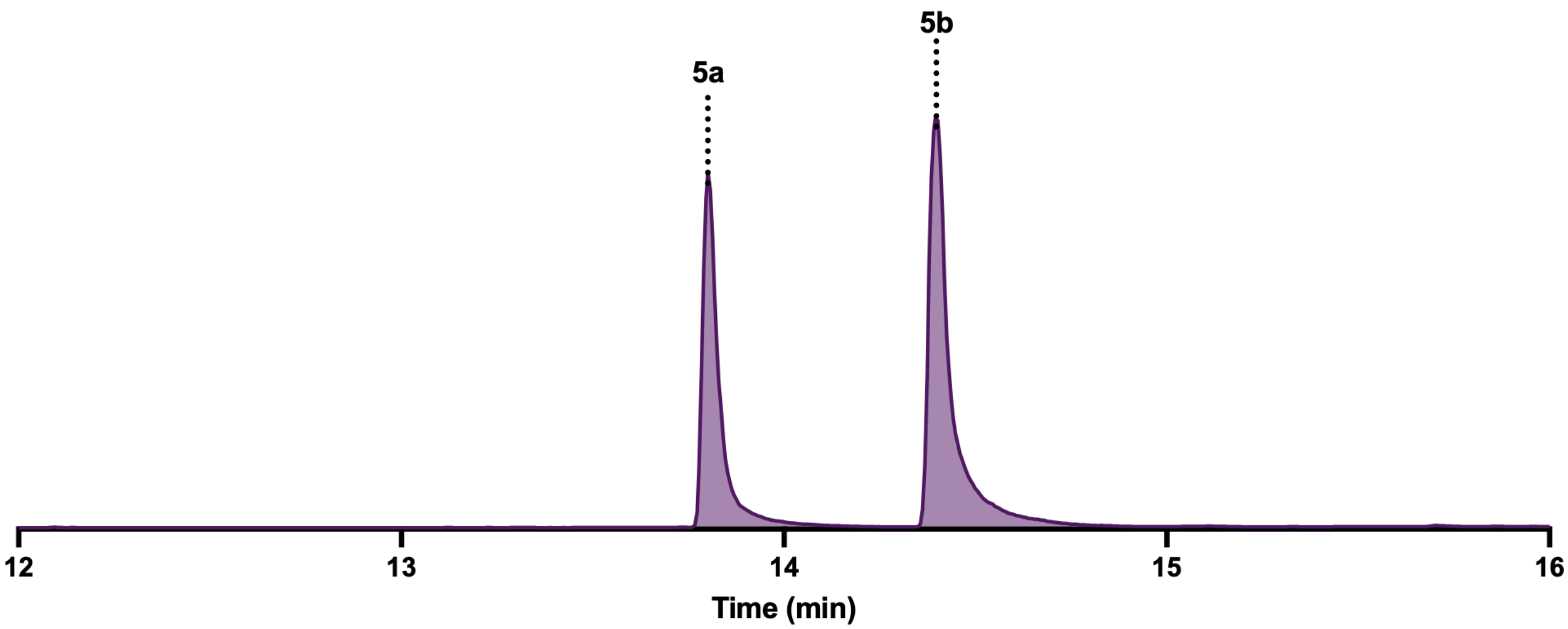

Figure S11. GCMS base-peak chromatogram of *B. lecontii* crude extract. Peaks corresponding to particular alkaloids are denoted with the compound number.

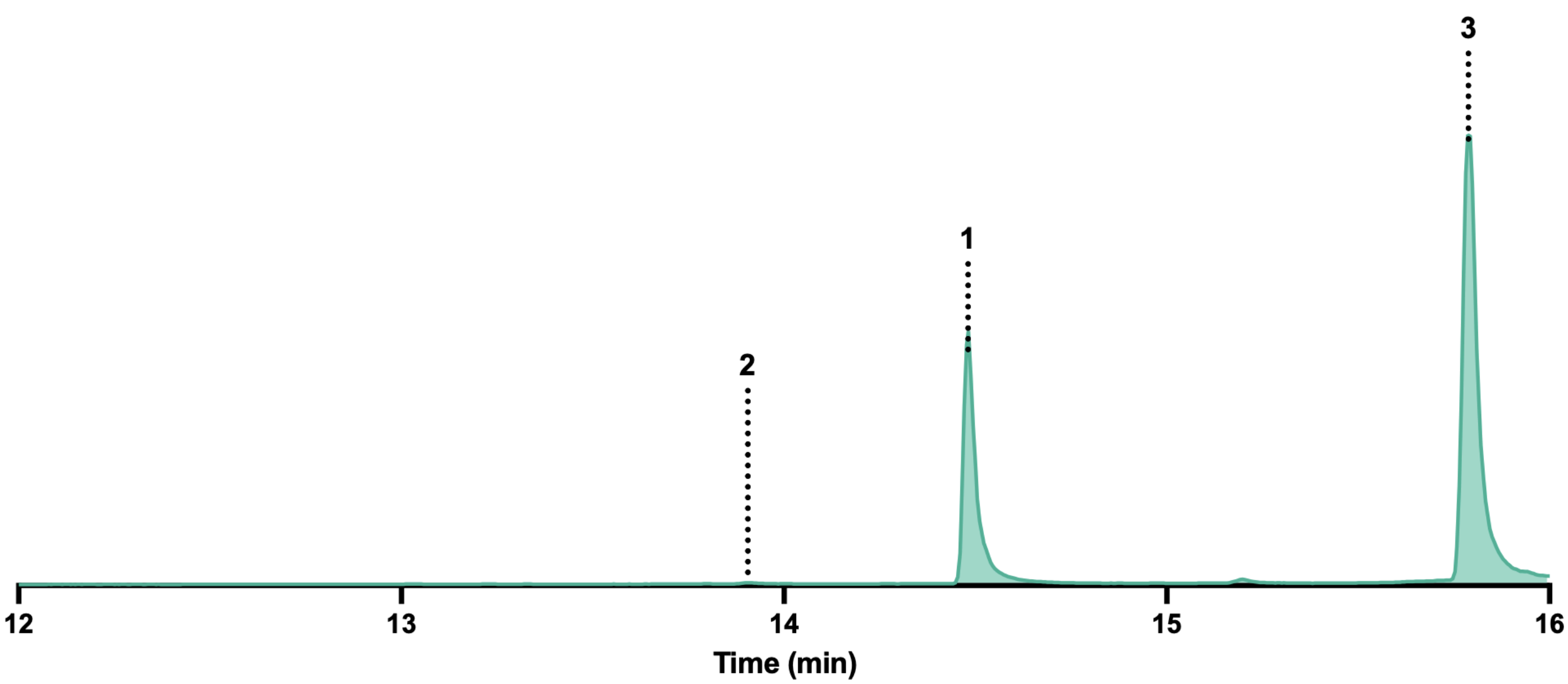

Figure S12. GCMS base–peak chromatogram of *B. petesata* crude extract. Peaks corresponding to particular alkaloids are denoted with the compound number.

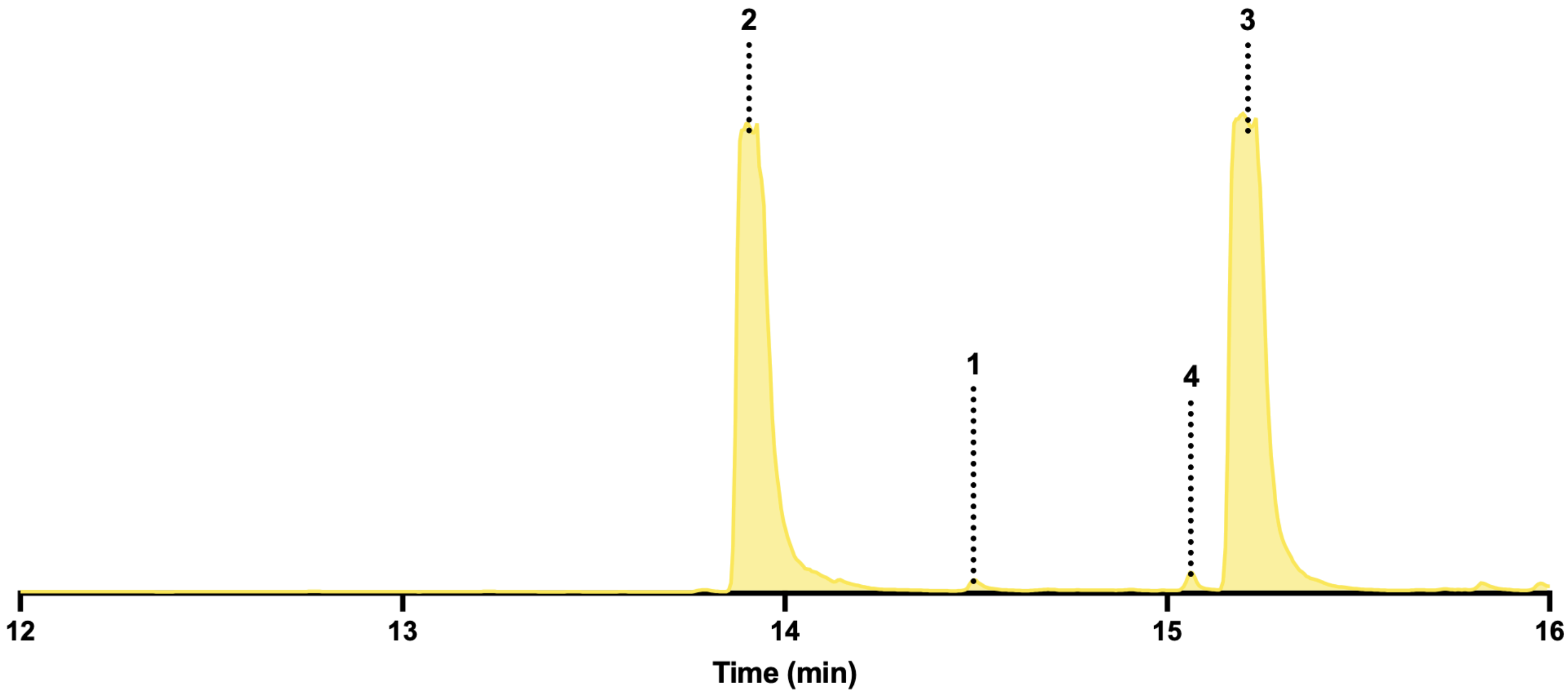

Figure S13. GCMS base-peak chromatogram of *B. producta* crude extract. Peaks corresponding to particular alkaloids are denoted with the compound number.

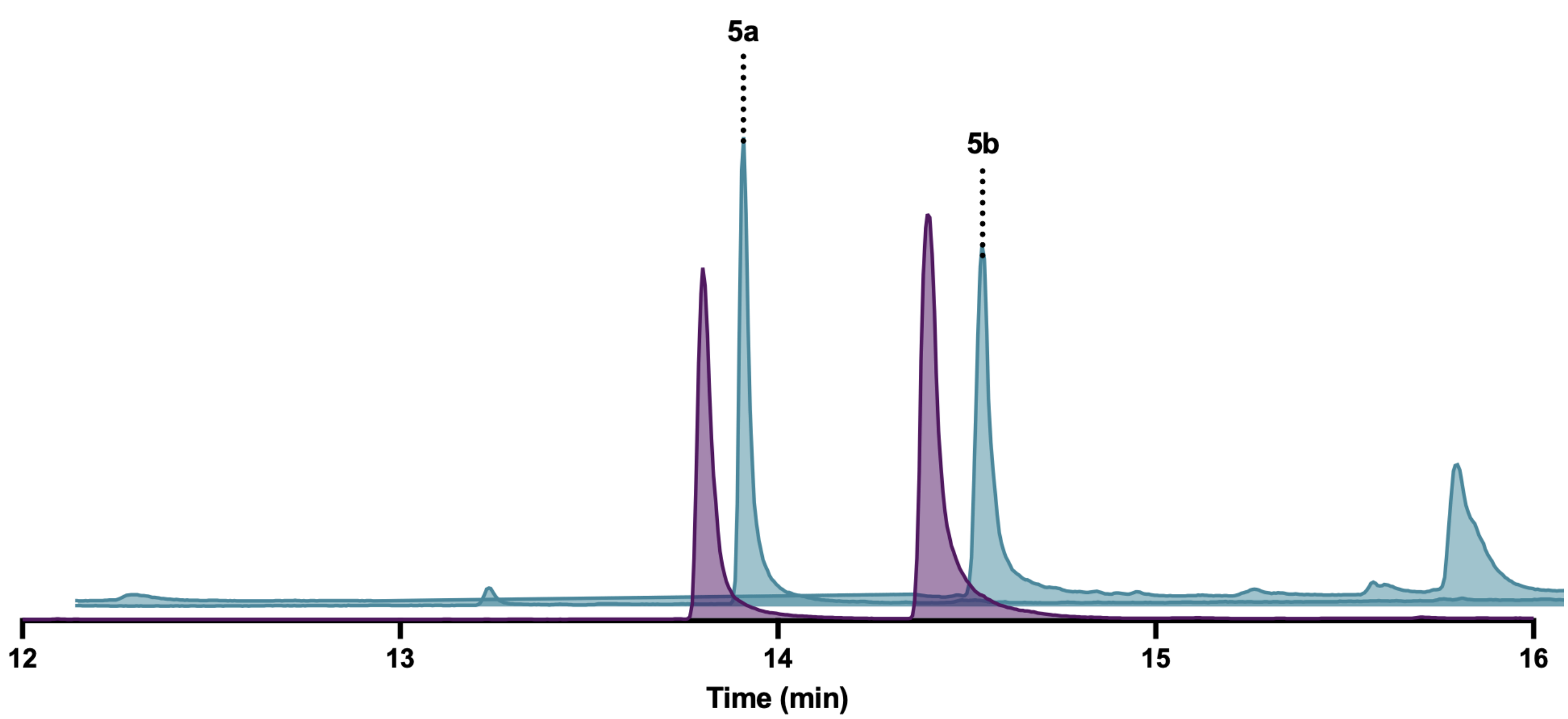

Figure S14. GCMS comparison of the base-peak chromatogram representing the deoxybuzonamine standards (bottom; purple trace) with a representative extract of *B. lecontii* (top; teal trace). The teal trace is offset by 0.15 min compared to the purple trace.

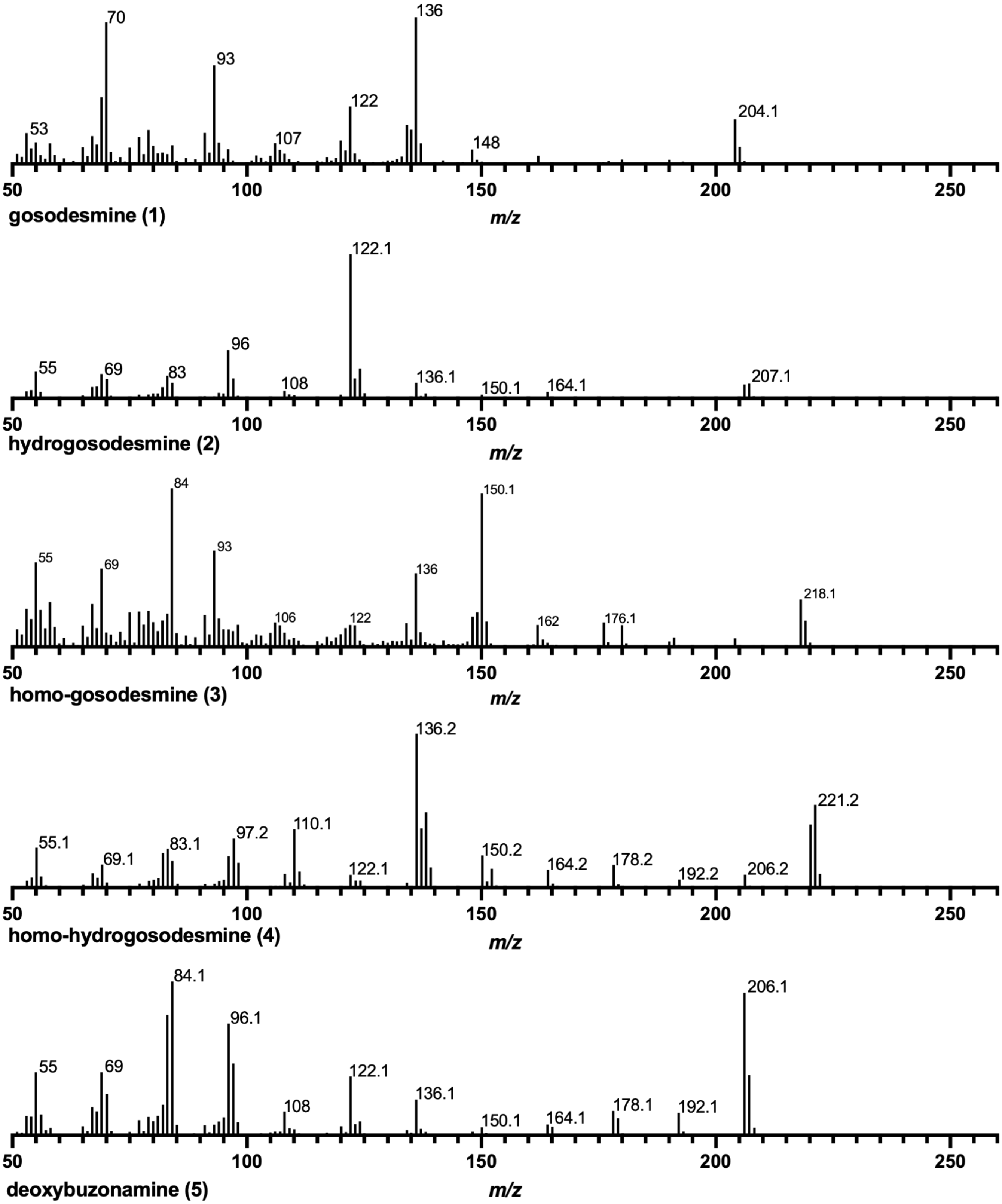

Figure S15. Representative GCMS spectra for compounds **1**–**5**.

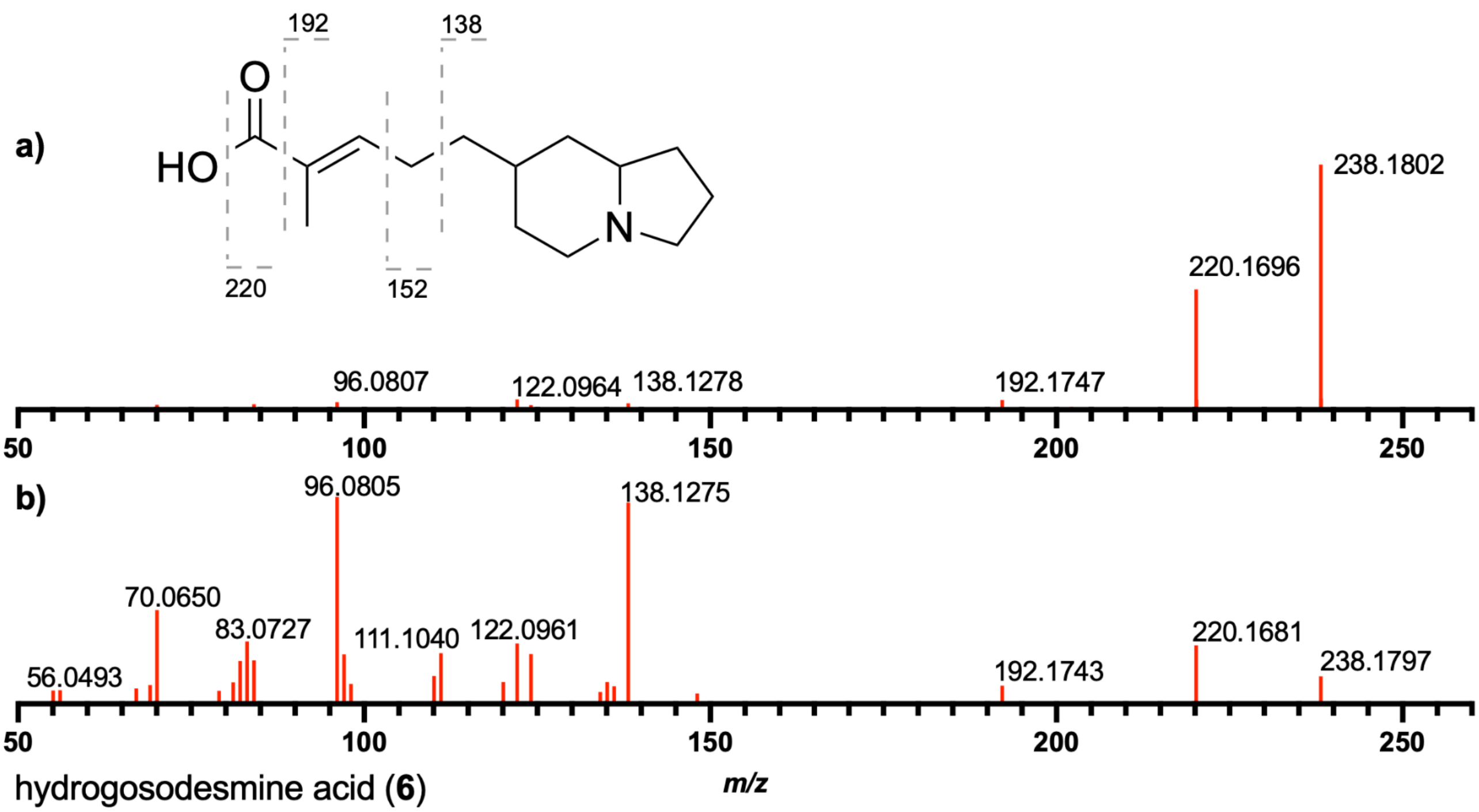

Figure S16. HR-LC-ESI-MSMS spectra of hydrogosodesmine acid (**6**) acquired on a Thermo Exploris 120 Orbitrap. **(A)** Spectrum obtained with a CID of 45 eV, and **(B)** spectrum obtained with a CID of 80 eV.

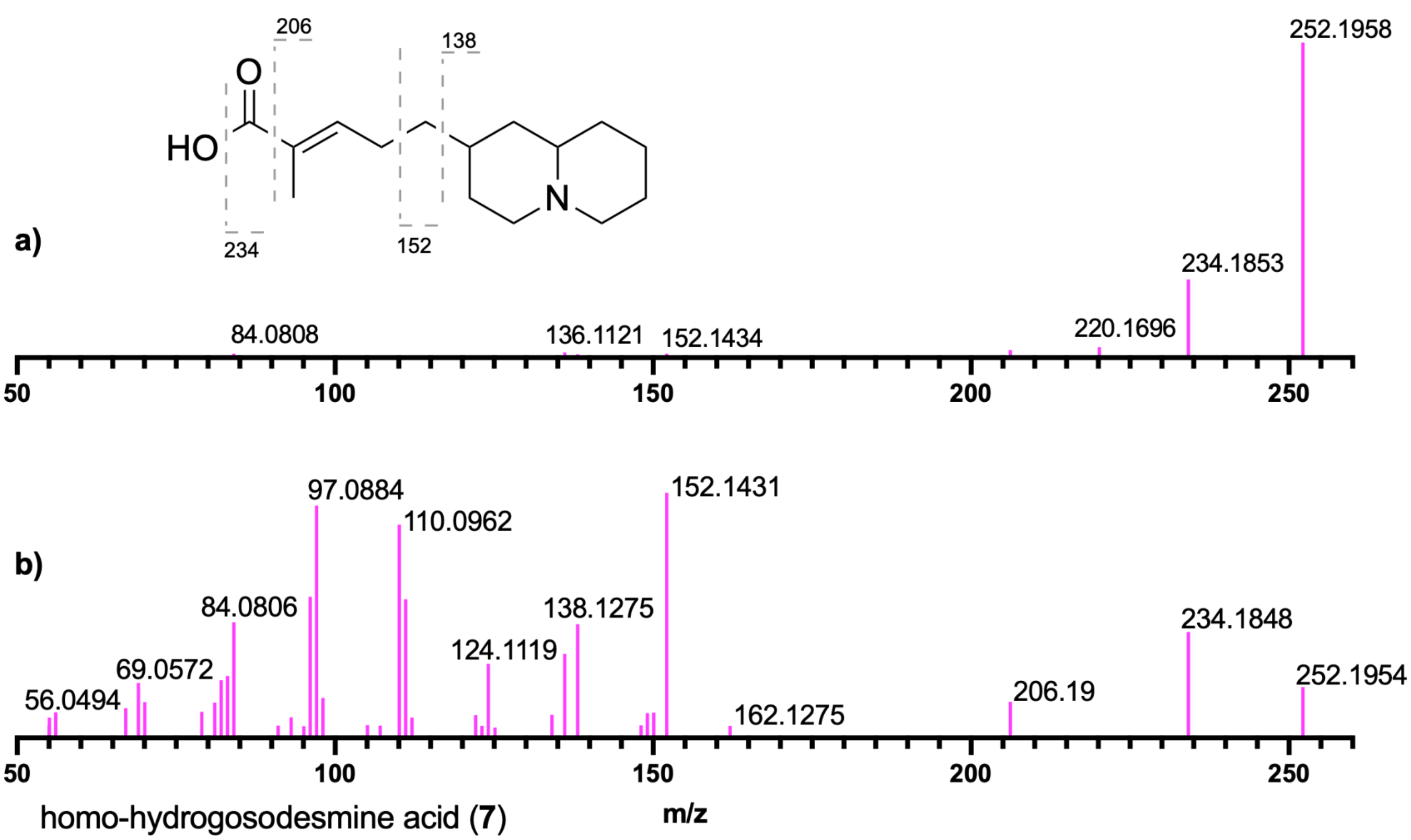

Figure S17. HR-LC-ESI-MSMS spectra of homo-hydrogosodesmine acid (**7**) acquired on a Thermo Exploris 120 Orbitrap. **(A)** Spectra obtained with a CID of 45 eV, and **(B)** spectrum obtained with a CID of 80 eV.

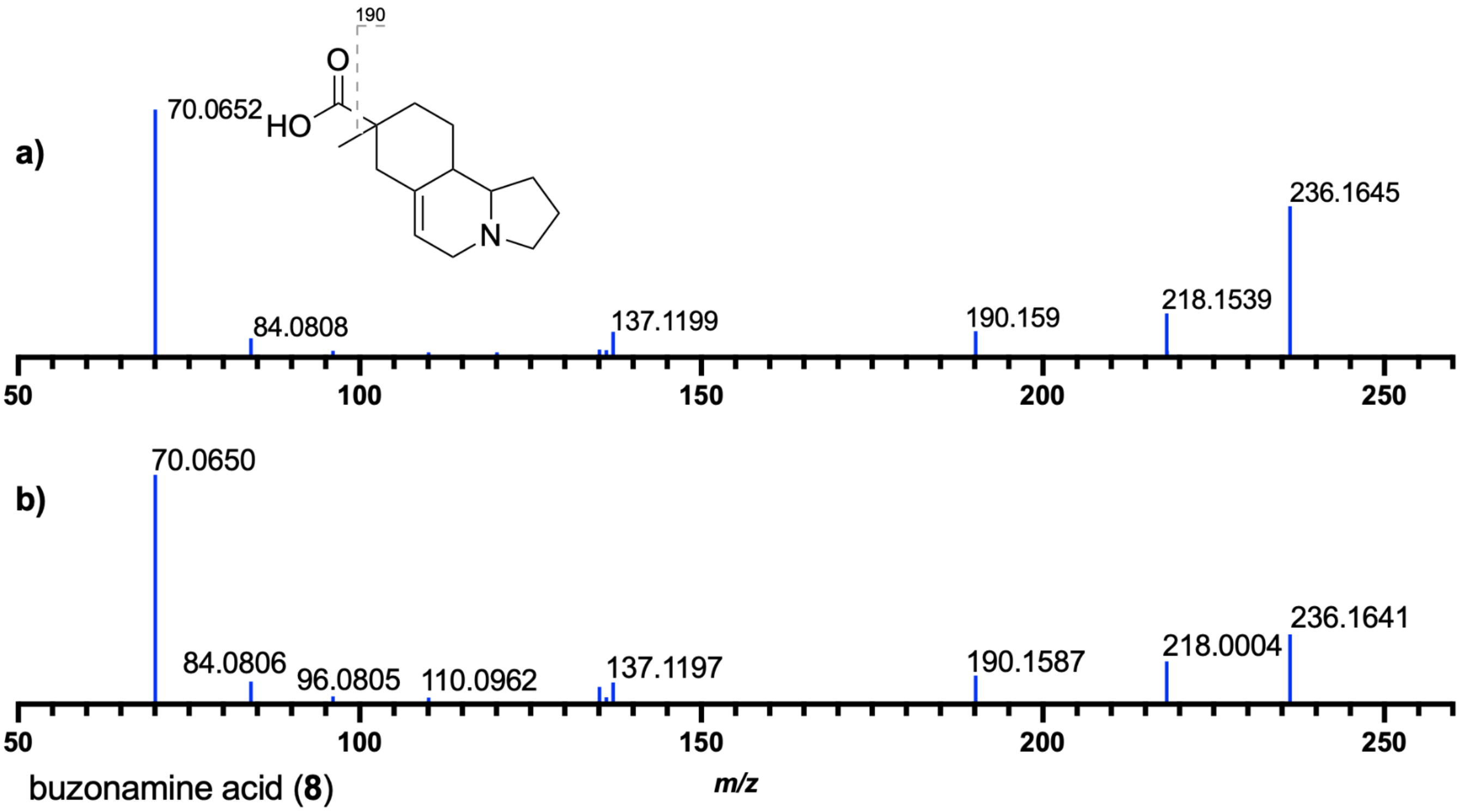

Figure S18. HR-LC-ESI-MSMS spectra of buzonamine acid (**8**) acquired on a Thermo Exploris 120 Orbitrap. **(A)** Spectrum obtained with a CID of 45 eV, and **(B)** spectrum obtained with a CID of 50 eV.

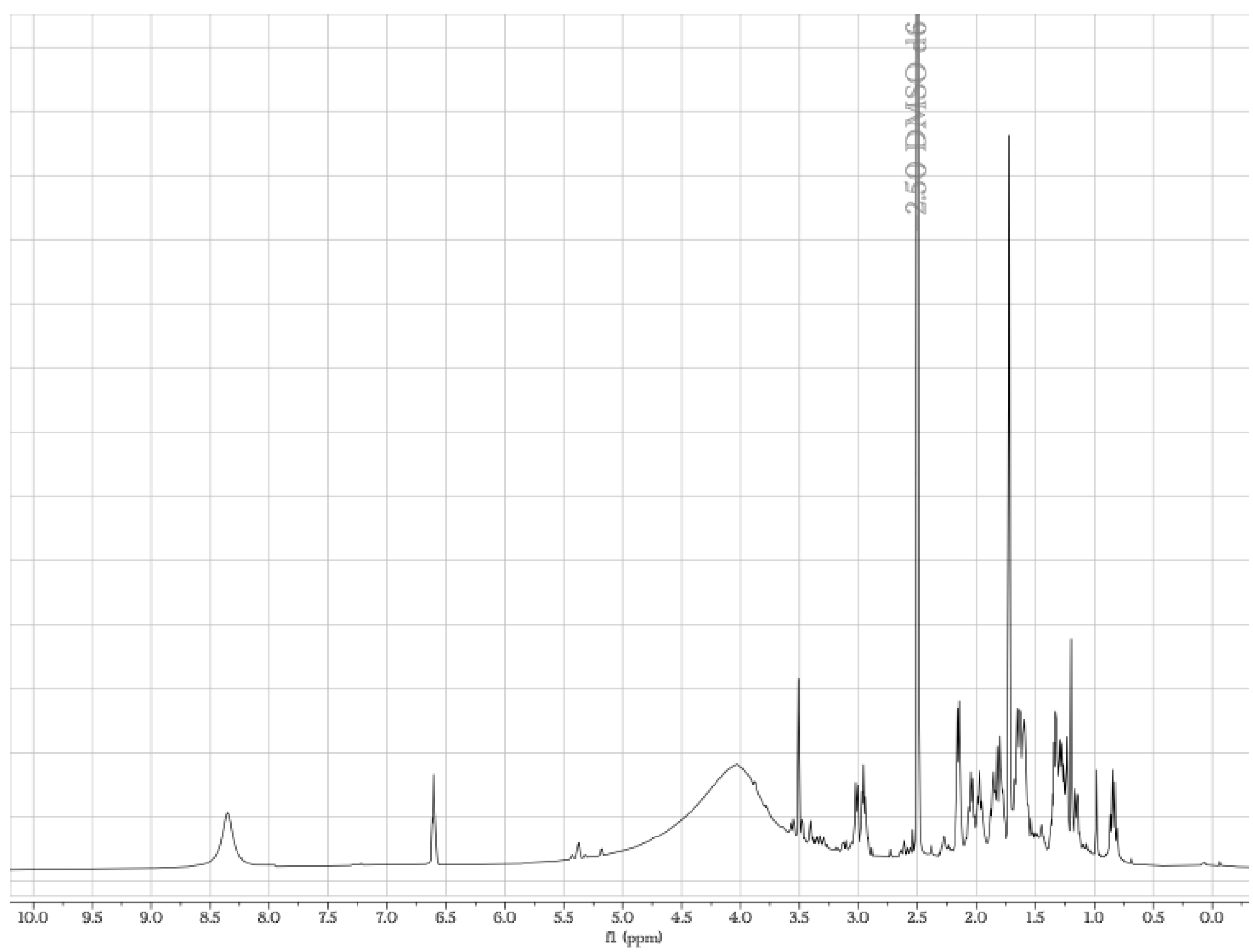

Figure S19. ^{1}H NMR spectrum of hydrogosodesmine acid (**6**) in d_6-DMSO (^{1}H 600 MHz). ns = 64

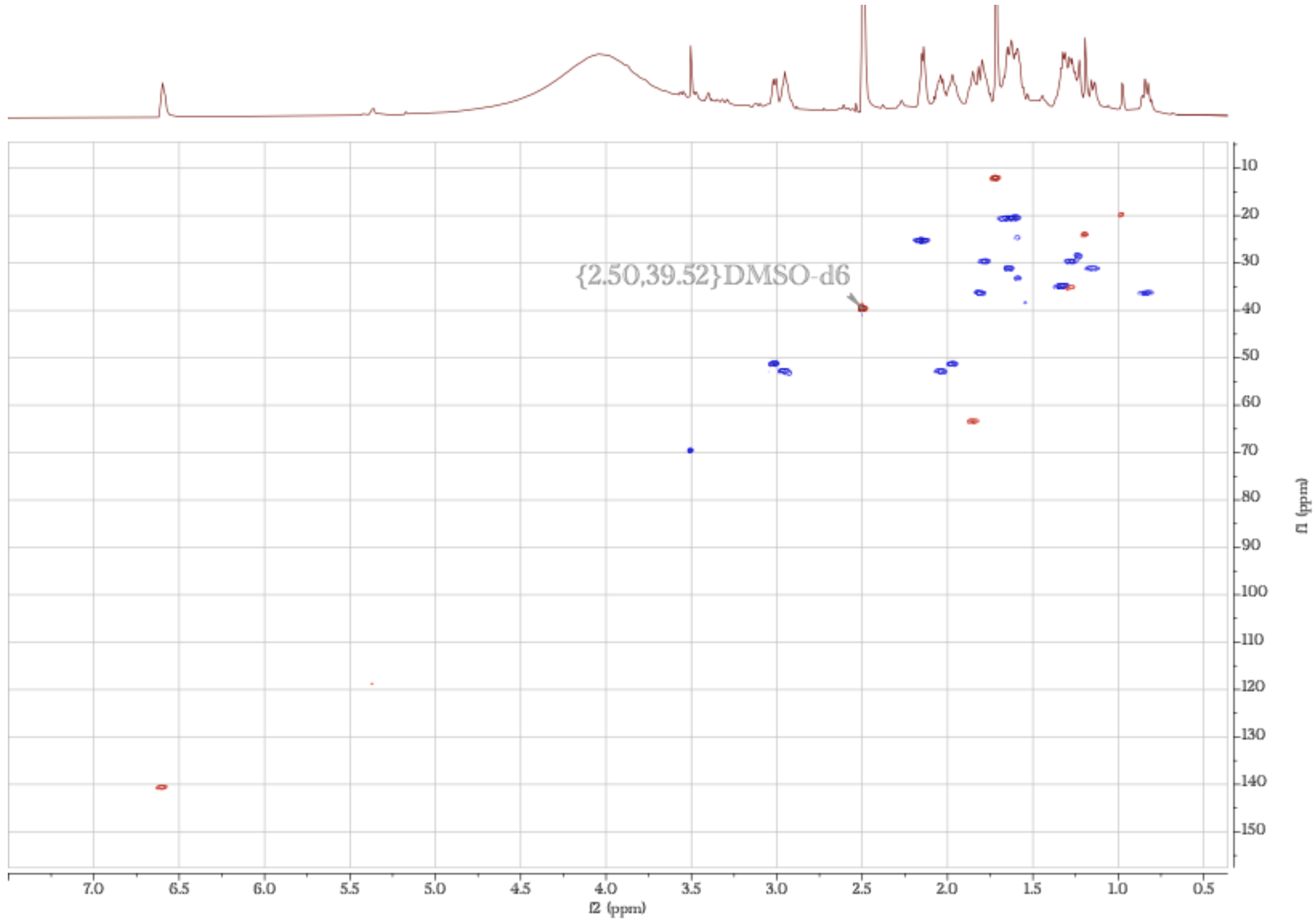

Figure S20. gHSQC NMR spectrum of hydrogosodesmine acid (**6**) in d_6-DMSO (^{1}H 600 MHz; ^{13}C 150 MHz). ns = 16

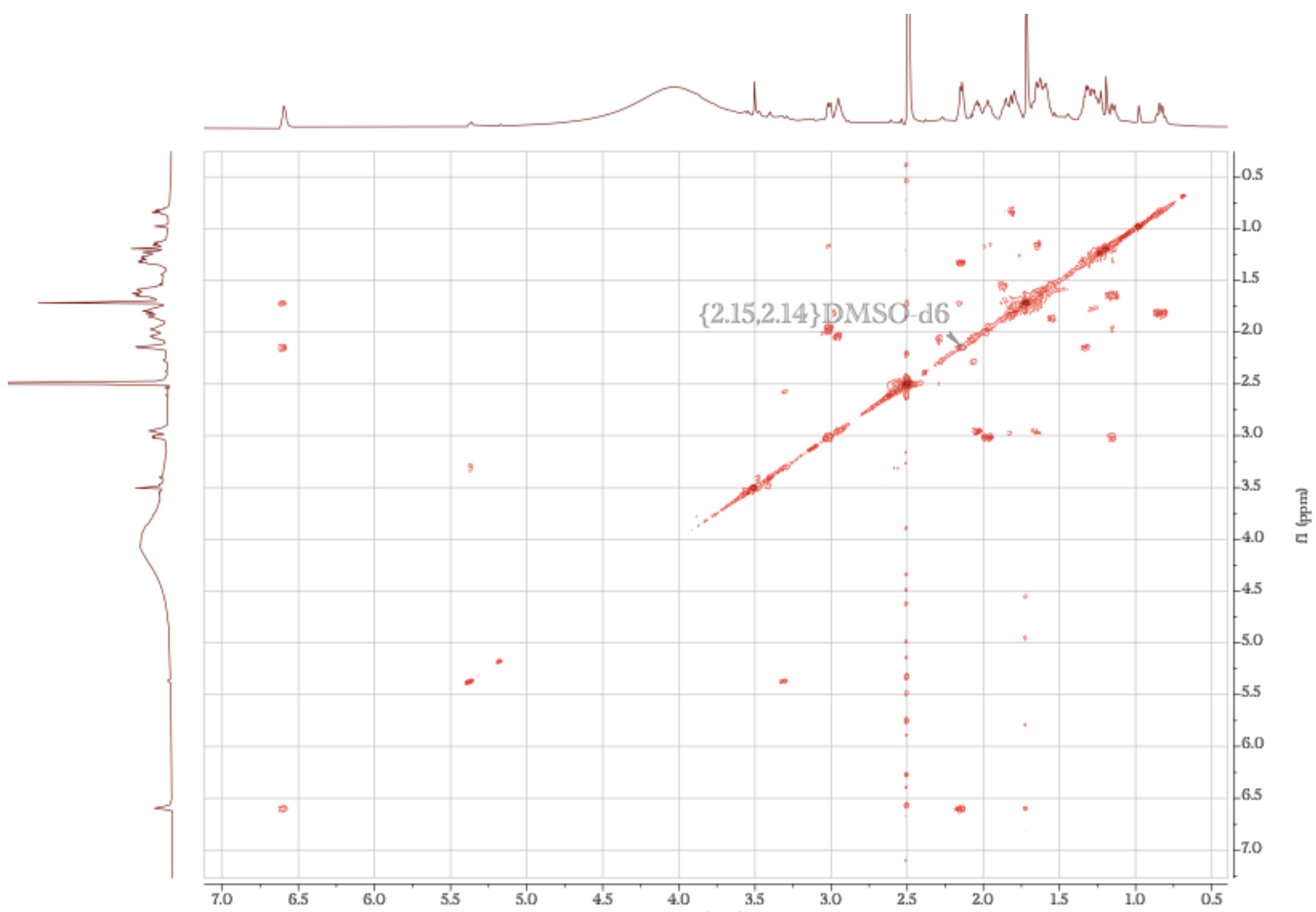

Figure S21. dqfCOSY NMR spectrum of hydrogosodesmine acid (**6**) in d_6-DMSO (^{1}H 600 MHz). ns = 8

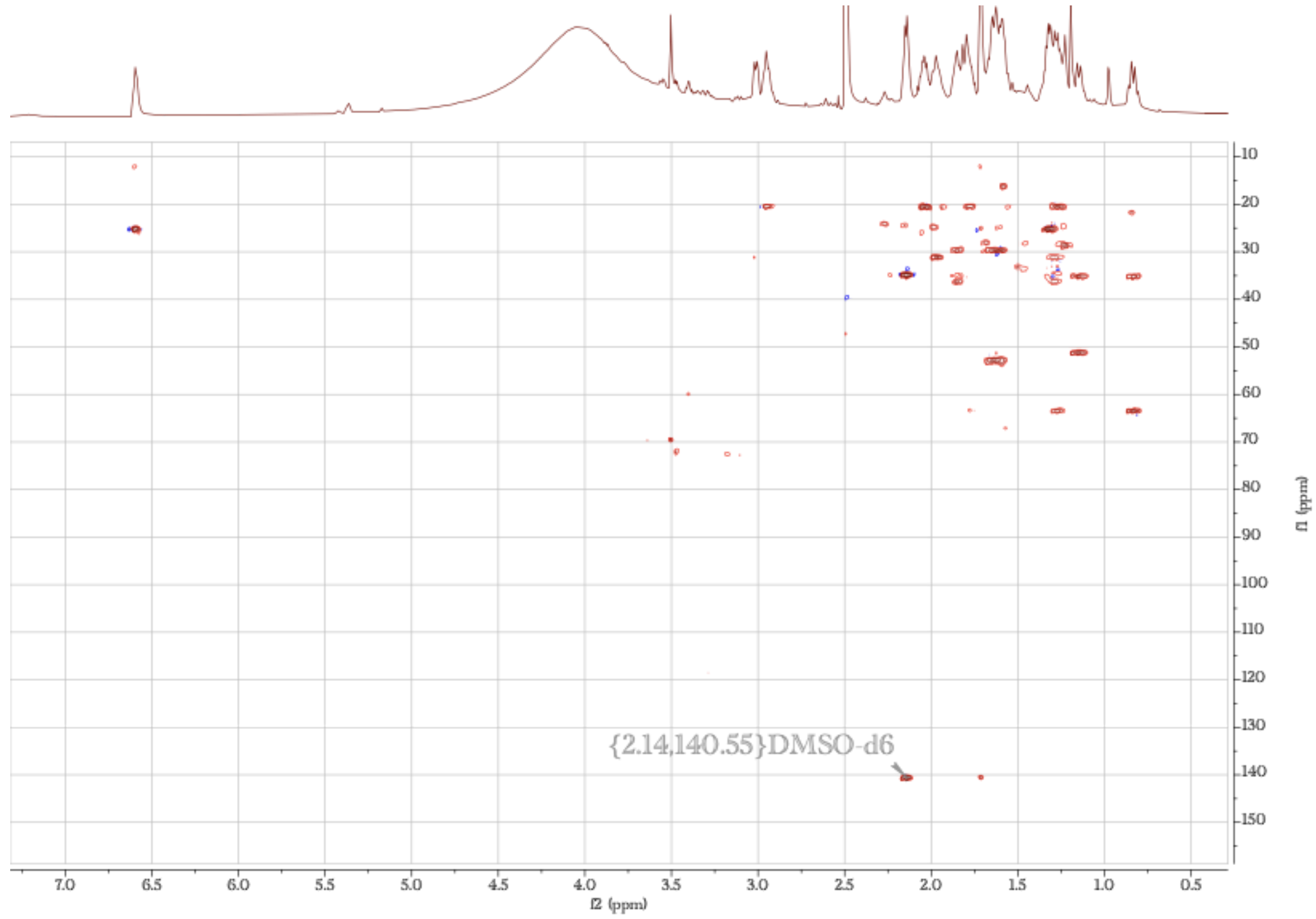

Figure S22. H2BC NMR spectrum of hydrogosodesmine acid (**6**) in d_6-DMSO (^{1}H 600 MHz; ^{13}C 150 MHz). ns = 128

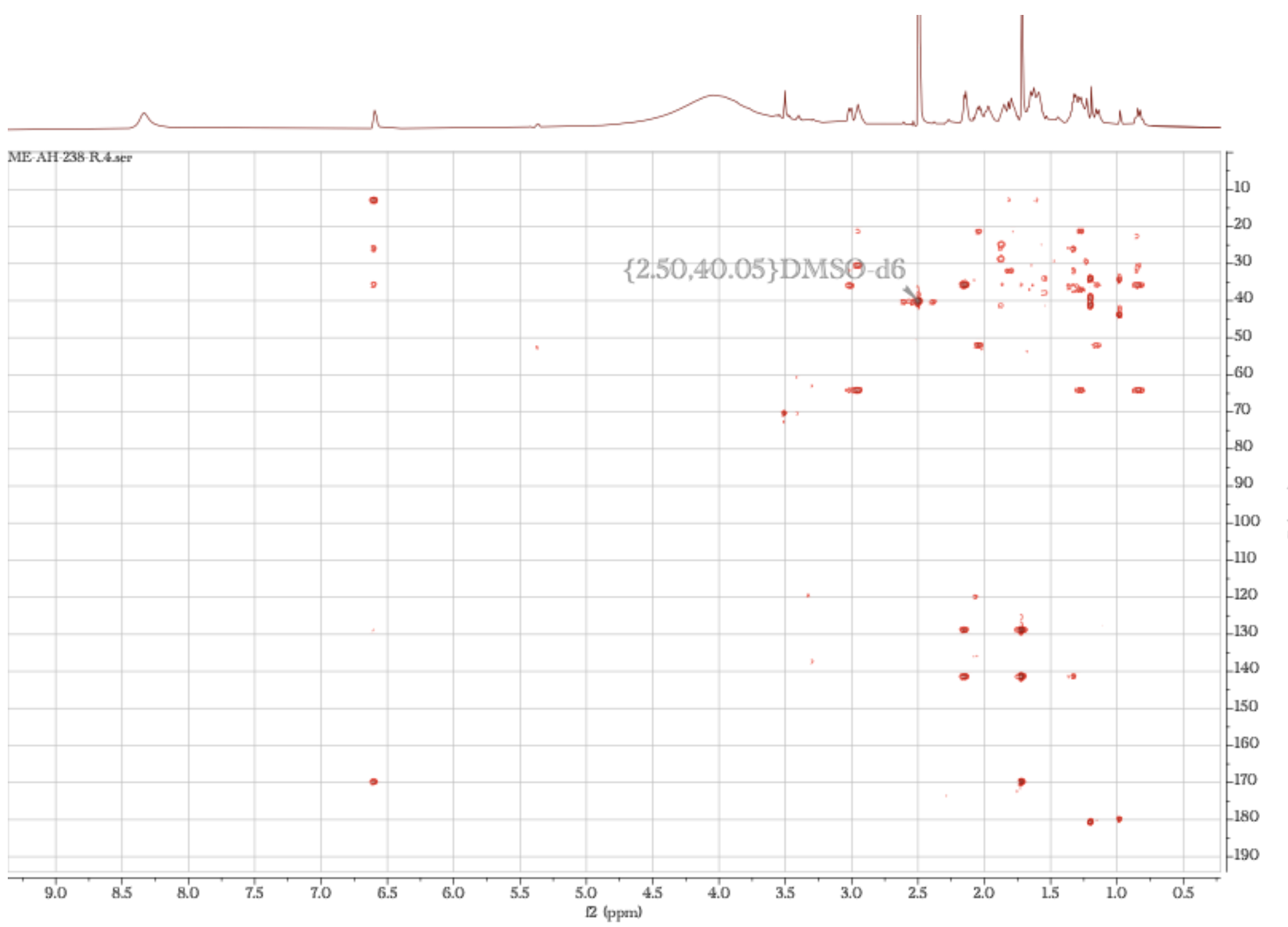

Figure S23. HMBC NMR spectrum of hydrogosodesmine acid (**6**) in d_6-DMSO (^{1}H 600 MHz; ^{13}C 150 MHz). ns = 128

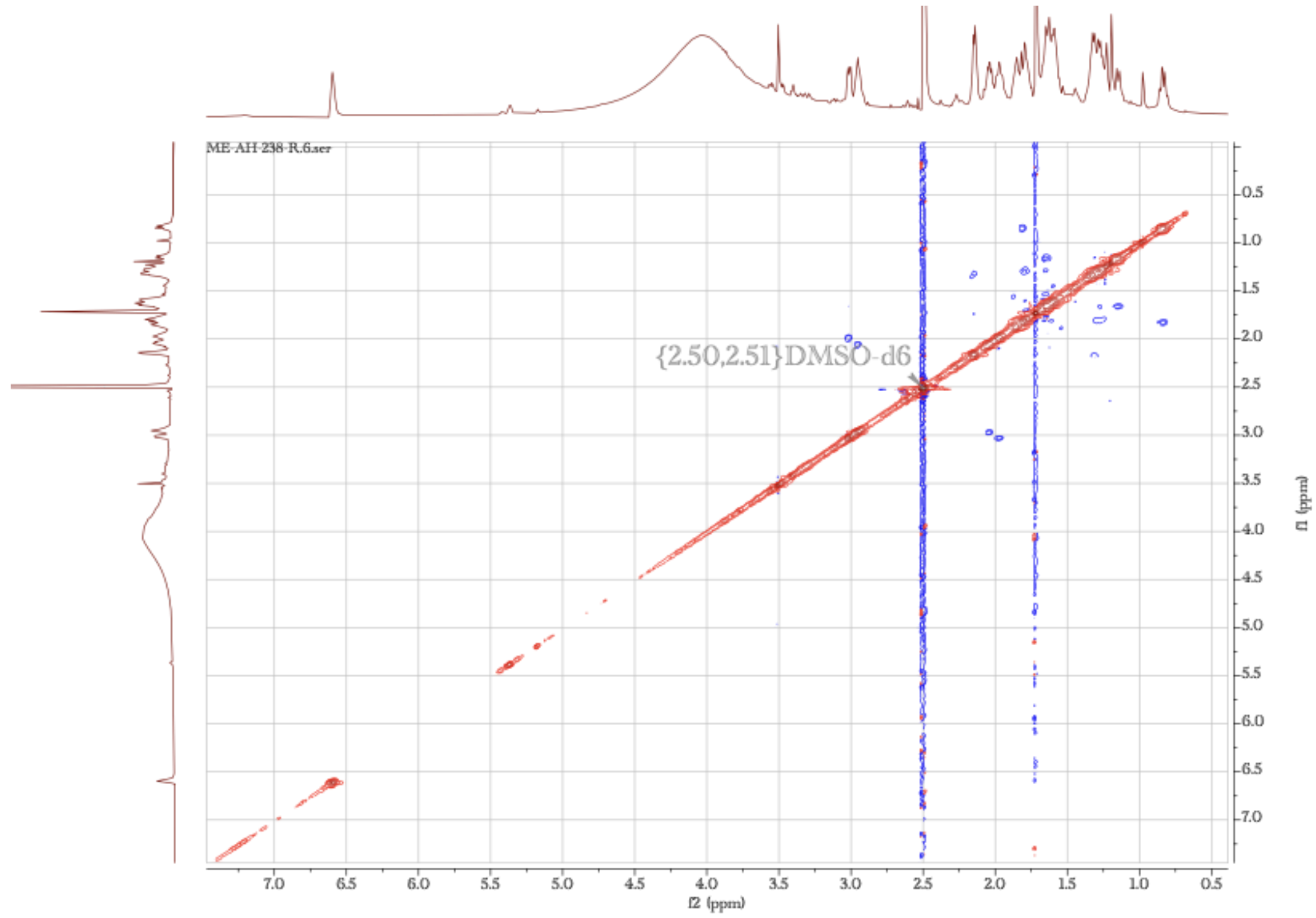

Figure S24. easyROESY NMR spectrum of hydrogosodesmine acid (**6**) in d_6-DMSO (^{1}H 600 MHz). ns = 96

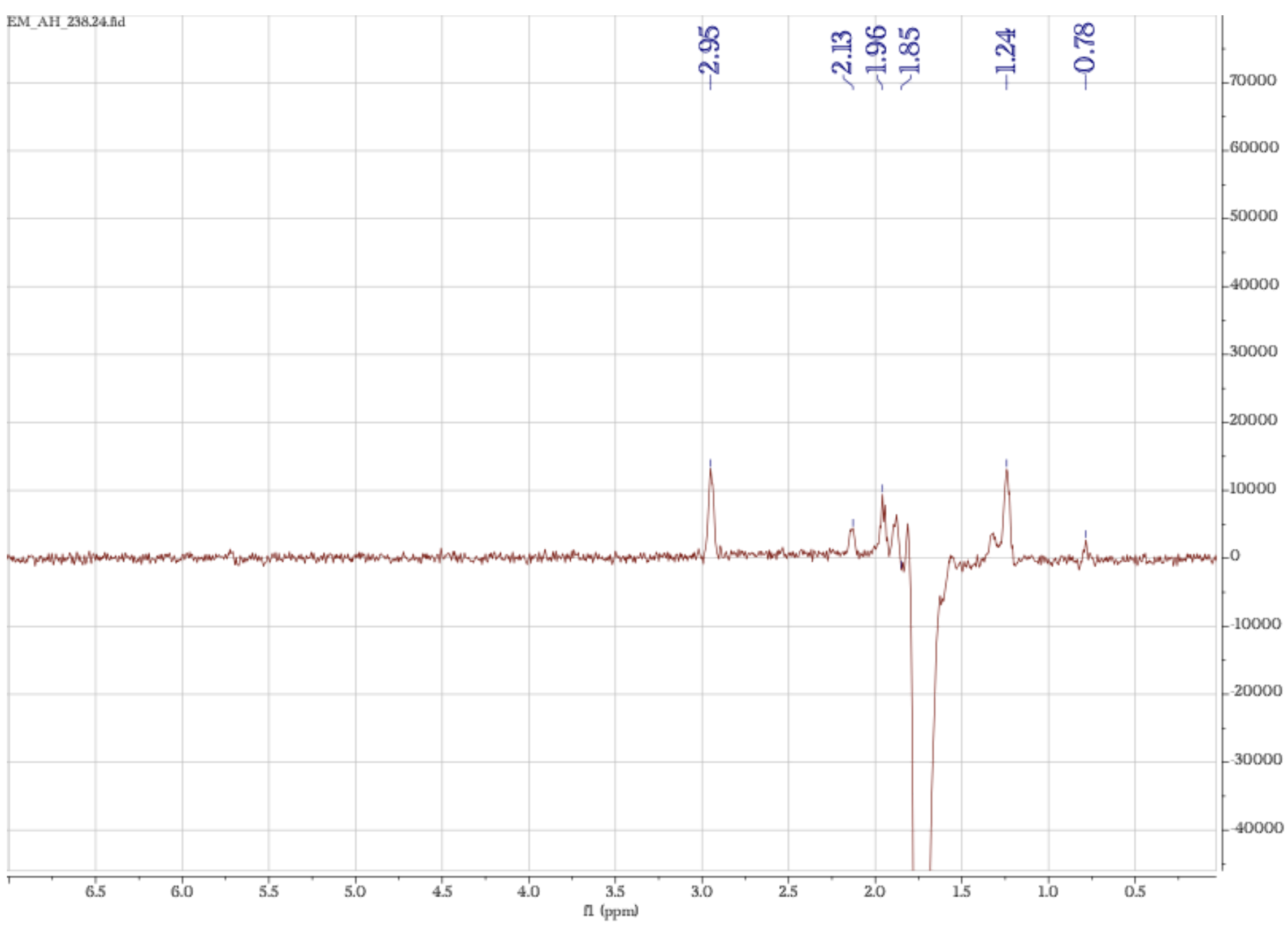

Figure S25. 1D NOE NMR (irradiating H_3-14, 1.72 ppm) spectra of hydrogosodesmine acid (**6**) in d_6-DMSO (^{1}H 600 MHz) ns = 2048, mixing time 0.8 sec.

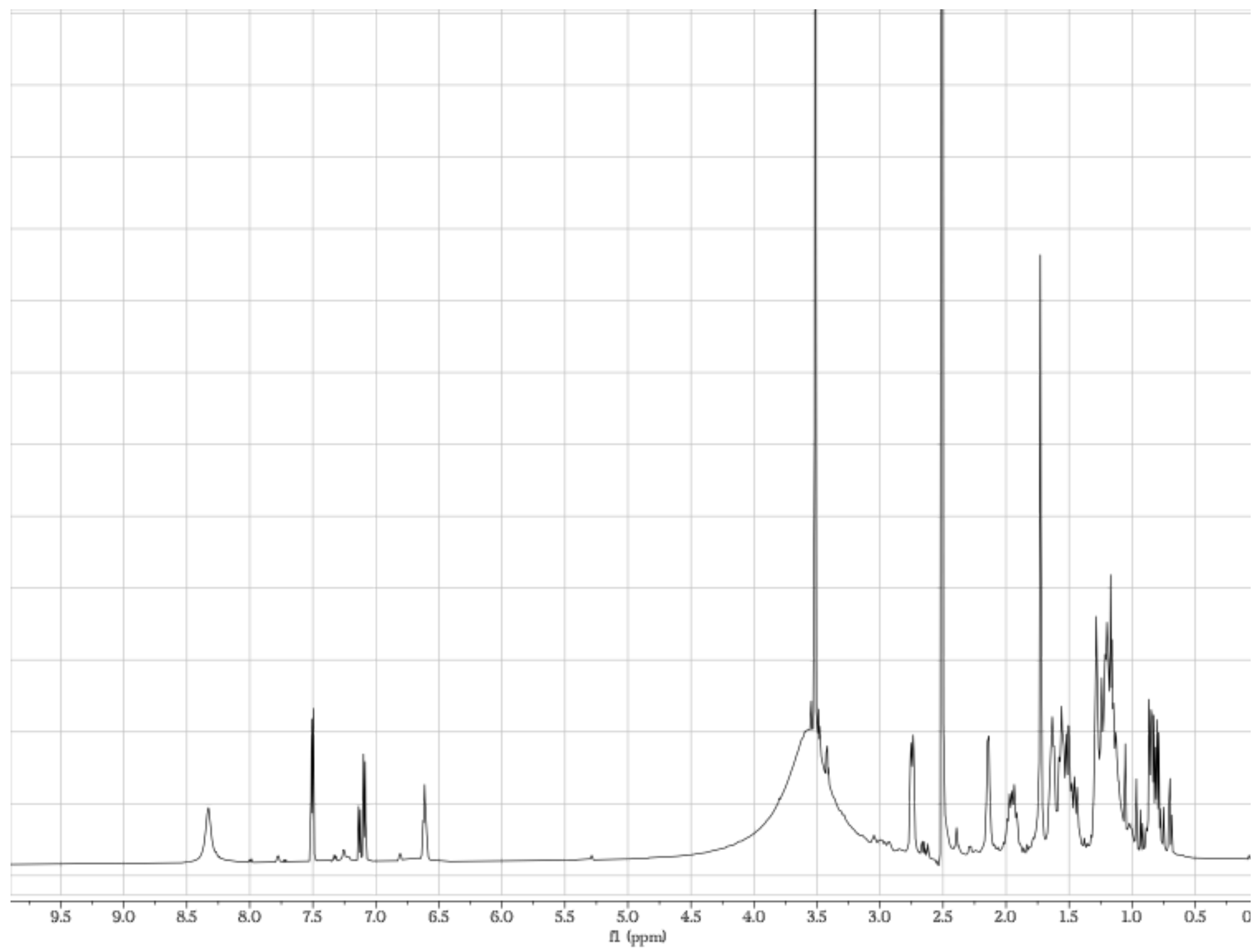

Figure S26. ^{1}H NMR spectrum of homo-hydrogosodesmine acid (**7**) in d_6-DMSO (^{1}H 600 MHz). ns = 64

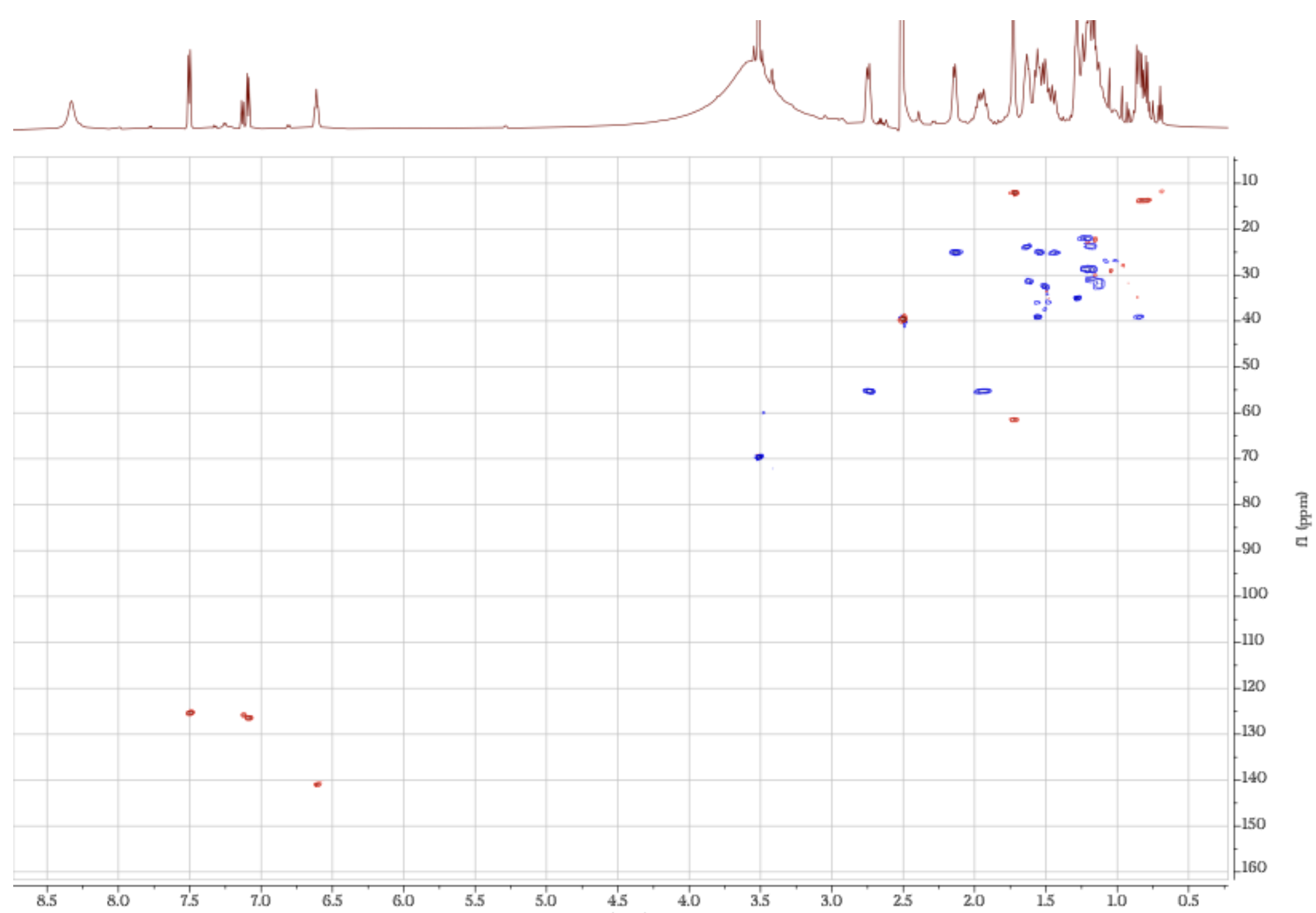

Figure S27. gHSQC NMR spectrum of homo-hydrogosodesmine acid (**7**) in d_6-DMSO (^{1}H 600 MHz; ^{13}C 150 MHz). ns = 16

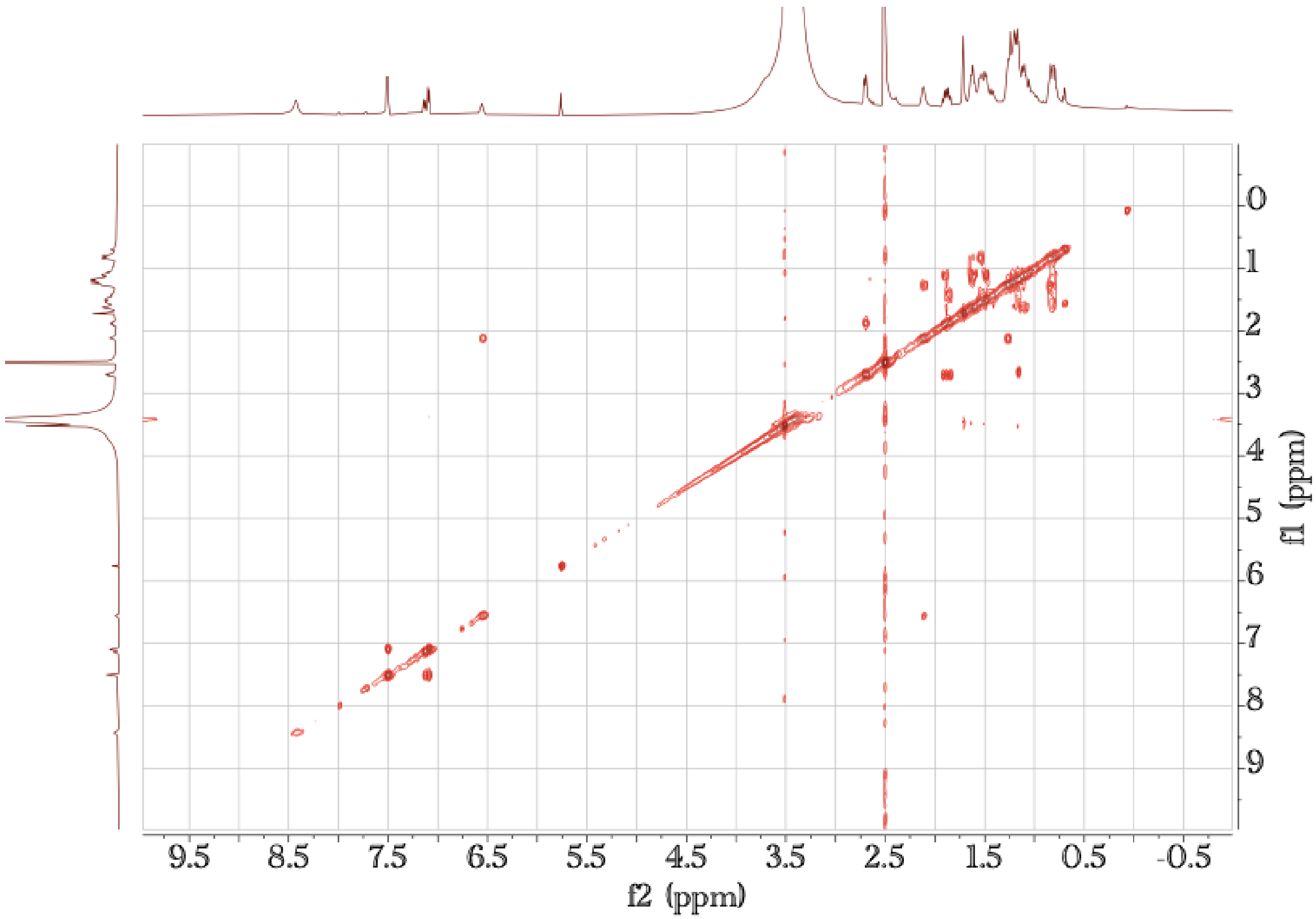

Figure S28. dqfCOSY NMR spectrum of homo-hydrogosodesmine acid (**7**) in d_6-DMSO (^{1}H 600 MHz). ns = 16

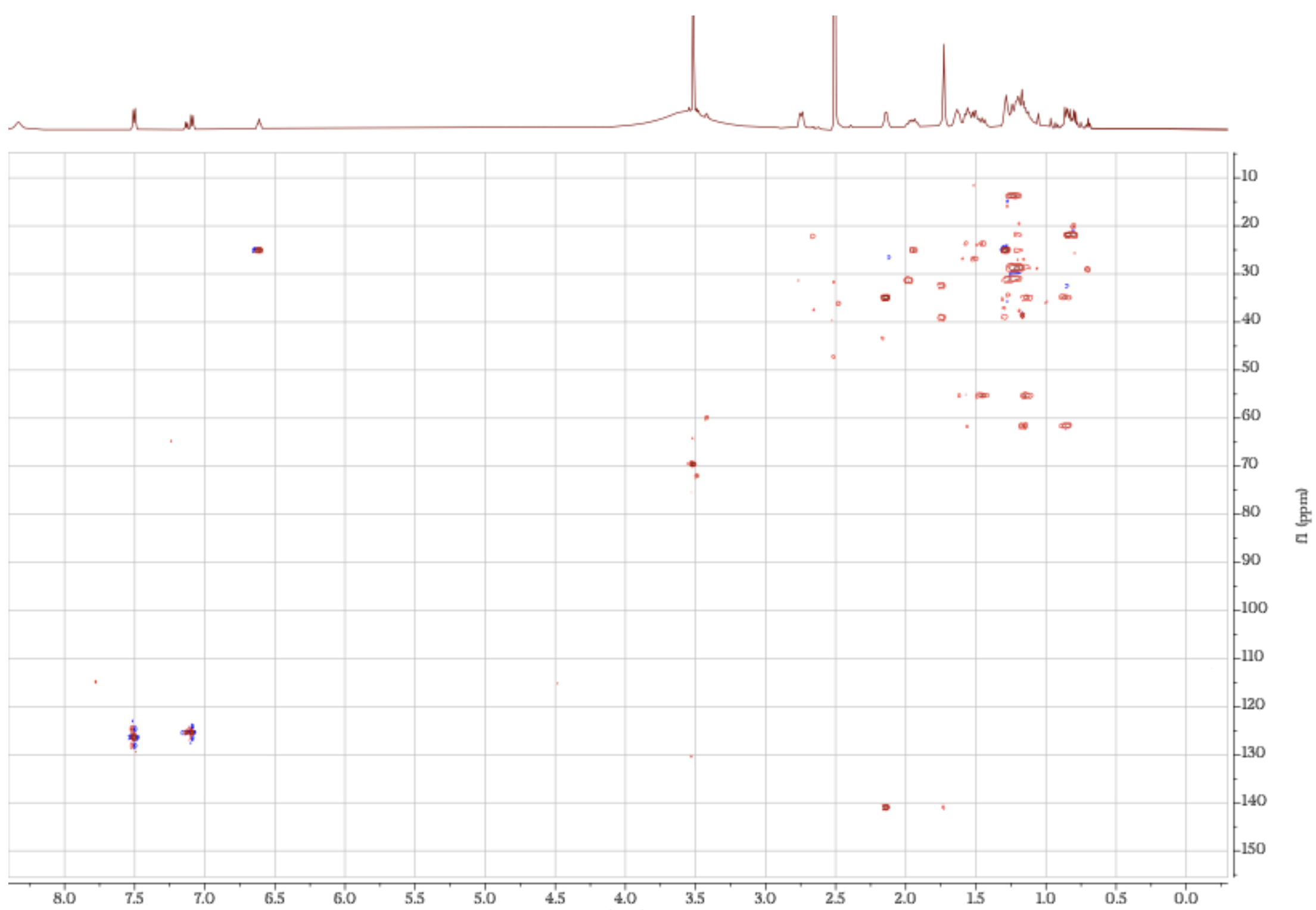

Figure S29. H2BC NMR spectrum of homo-hydrogosodesmine acid (**7**) in d_6-DMSO (^{1}H 600 MHz; ^{13}C 150 MHz). ns = 96

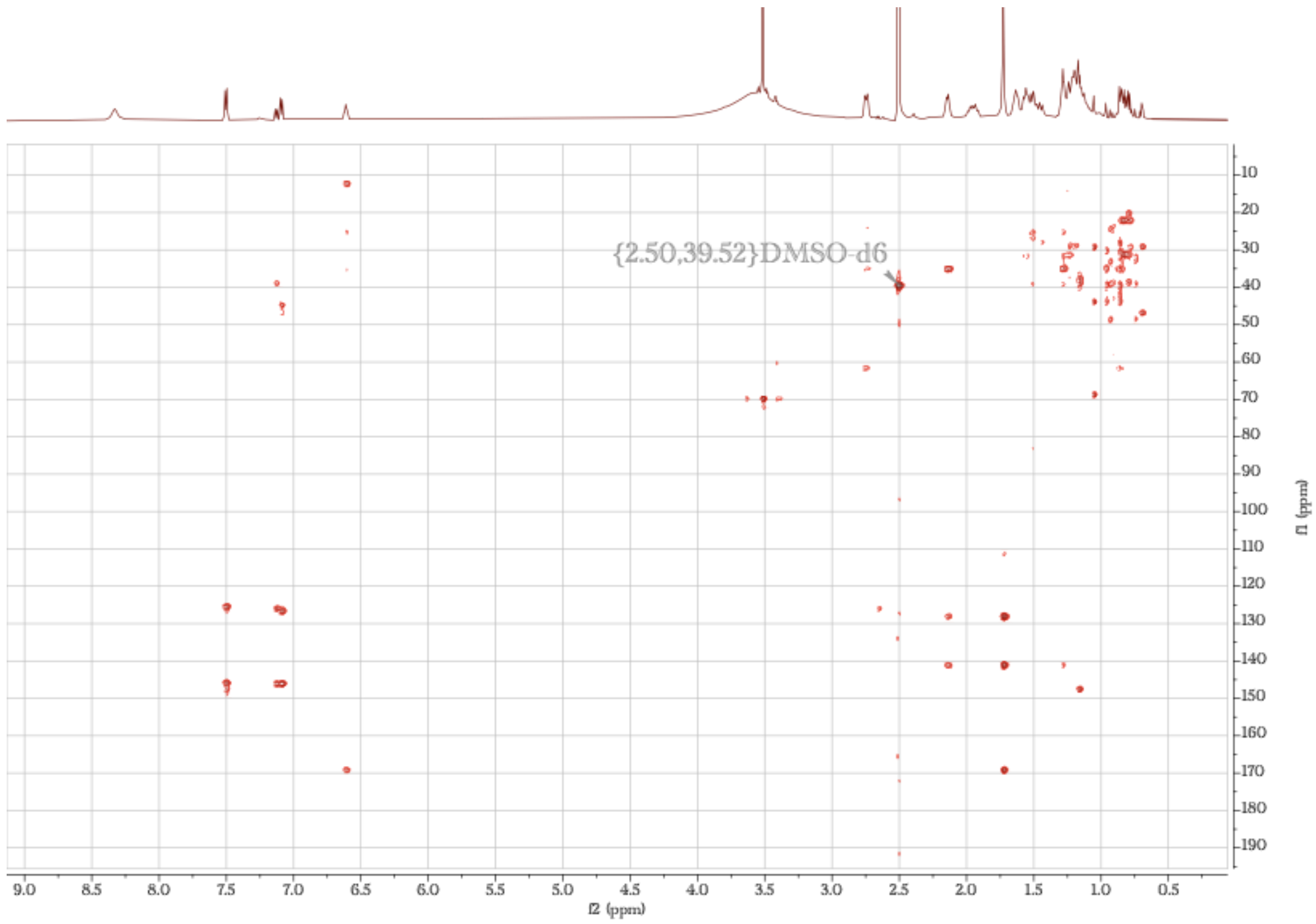

Figure S30. HMBC NMR spectrum of homo-hydrogosodesmine acid (**7**) in d_6-DMSO (^{1}H 600 MHz; ^{13}C 150 MHz). ns = 96

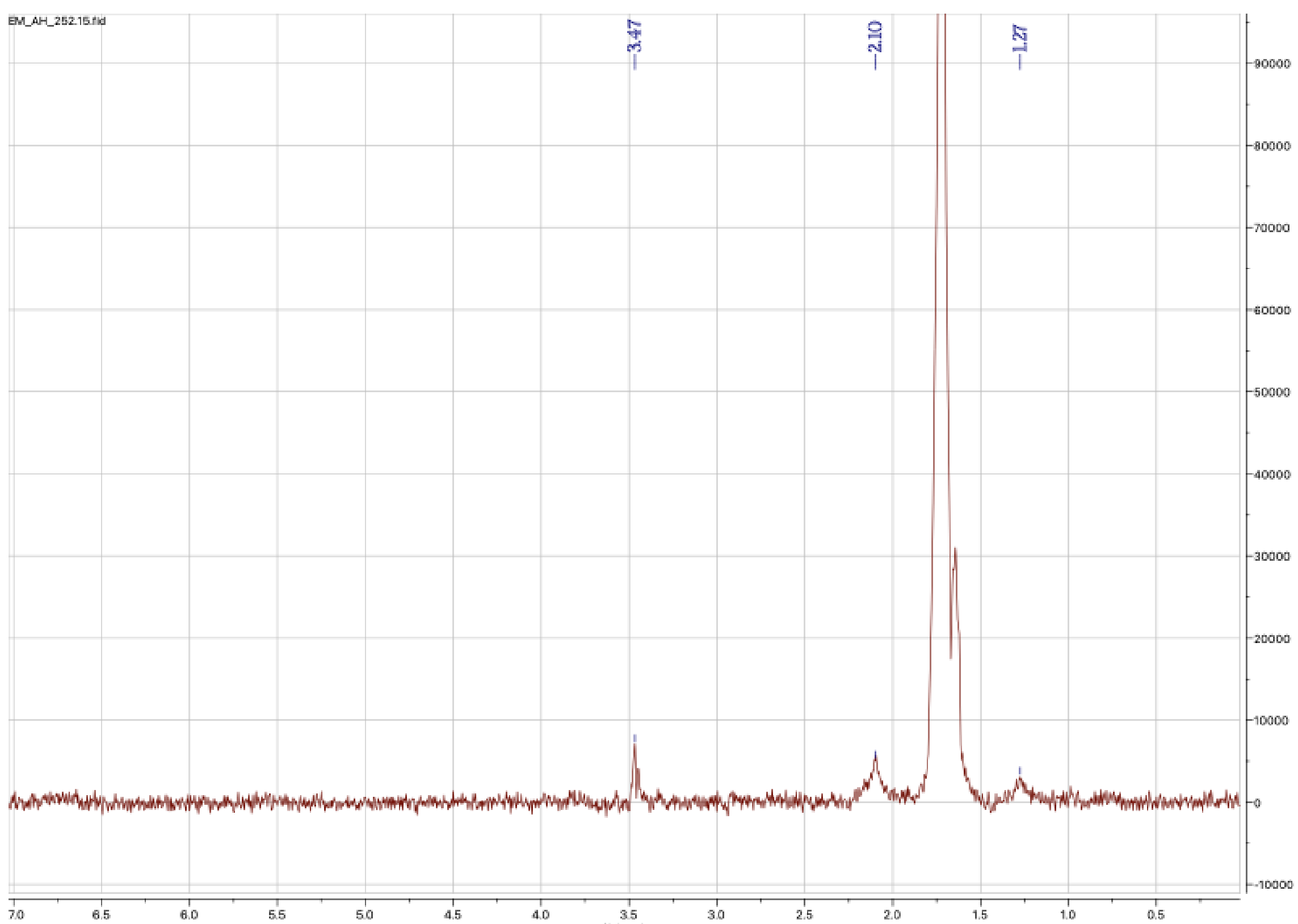

Figure S31. 1D NOE NMR (irradiating H_3-15, 1.72 ppm) spectrum of homo-hydrogosodesmine acid (**7**) in d_6-DMSO (^{1}H 600 MHz) ns = 2048, mixing time 0.8 sec.

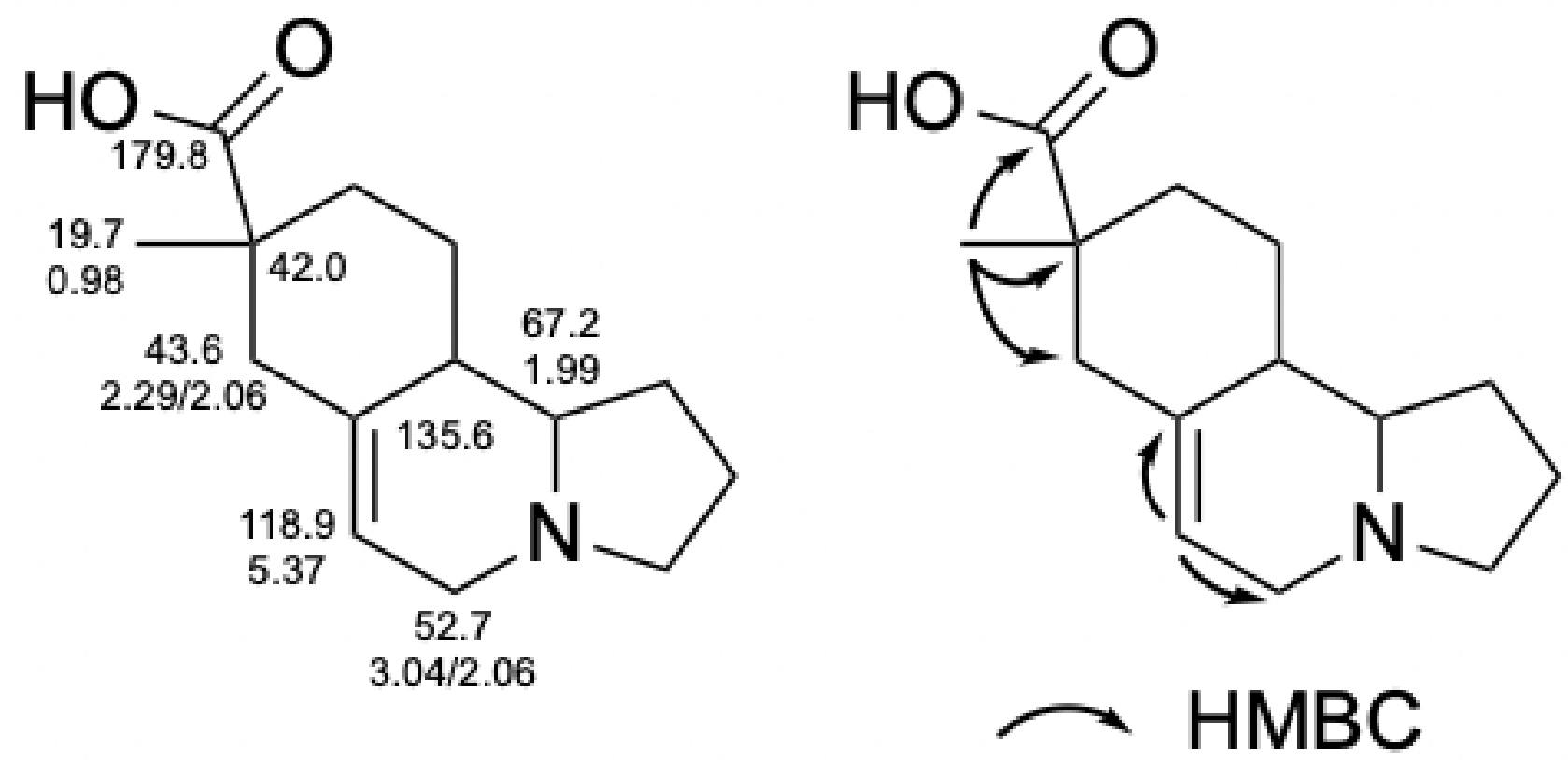

Figure S32. ^{1}H NMR and ^{13}C NMR chemical shifts for **8**, with associated 2D NMR correlations from **Figs. S19–S24**.

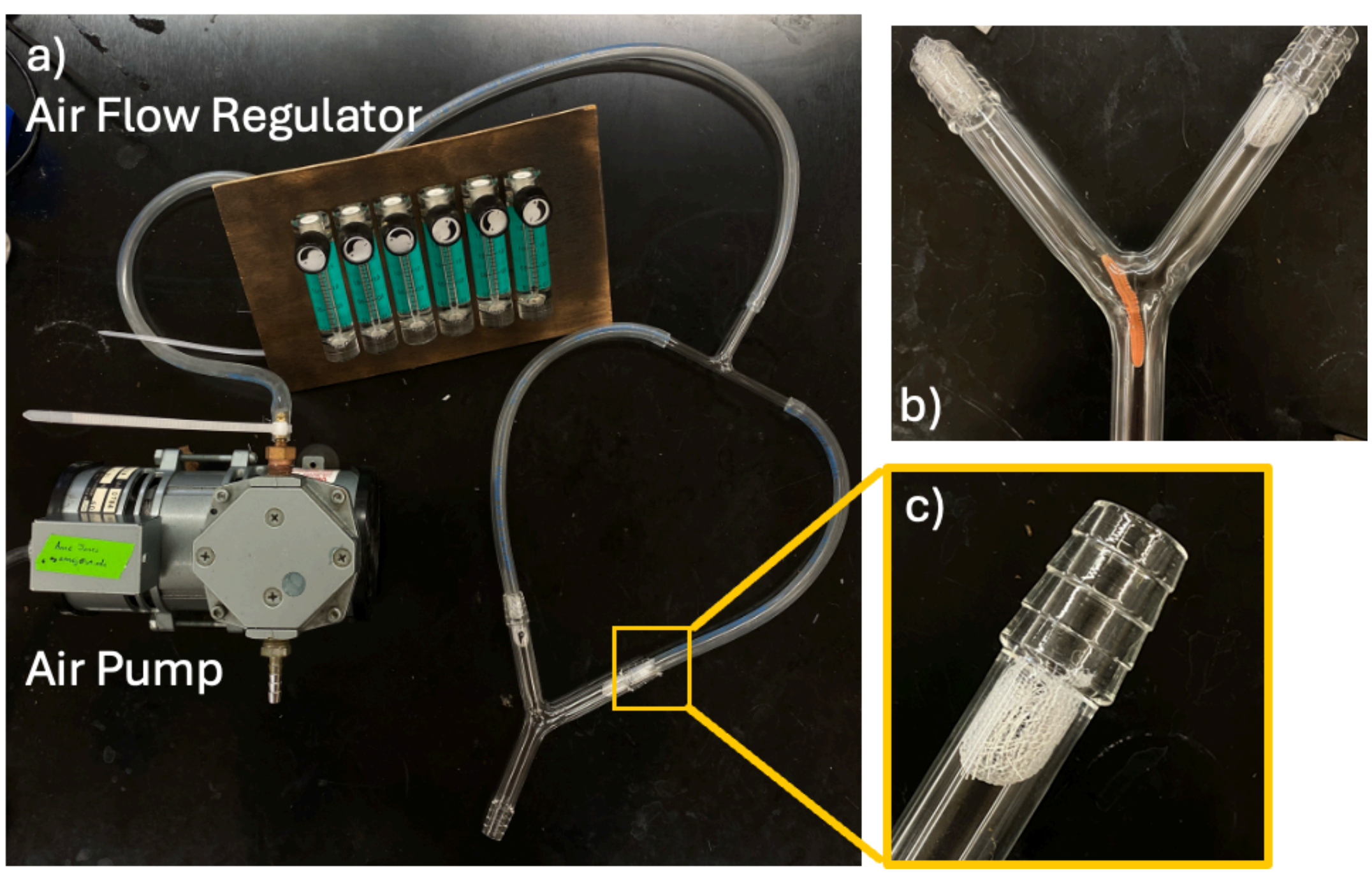

Figure S33. Y-tube choice assay setup. (**A**) Equipment setup for Y-tube choice assay; An air pump is connected to an airflow regulator. The regulated air is split at a glass T-junction that connects to both arms of the Y-tube: (**B**) *Brachycybe producta* choosing the Y-tube, and (**C**) a close-up of the wire and mesh cup used to contain a live millipede/compound disk in one arm of the Y-tube.

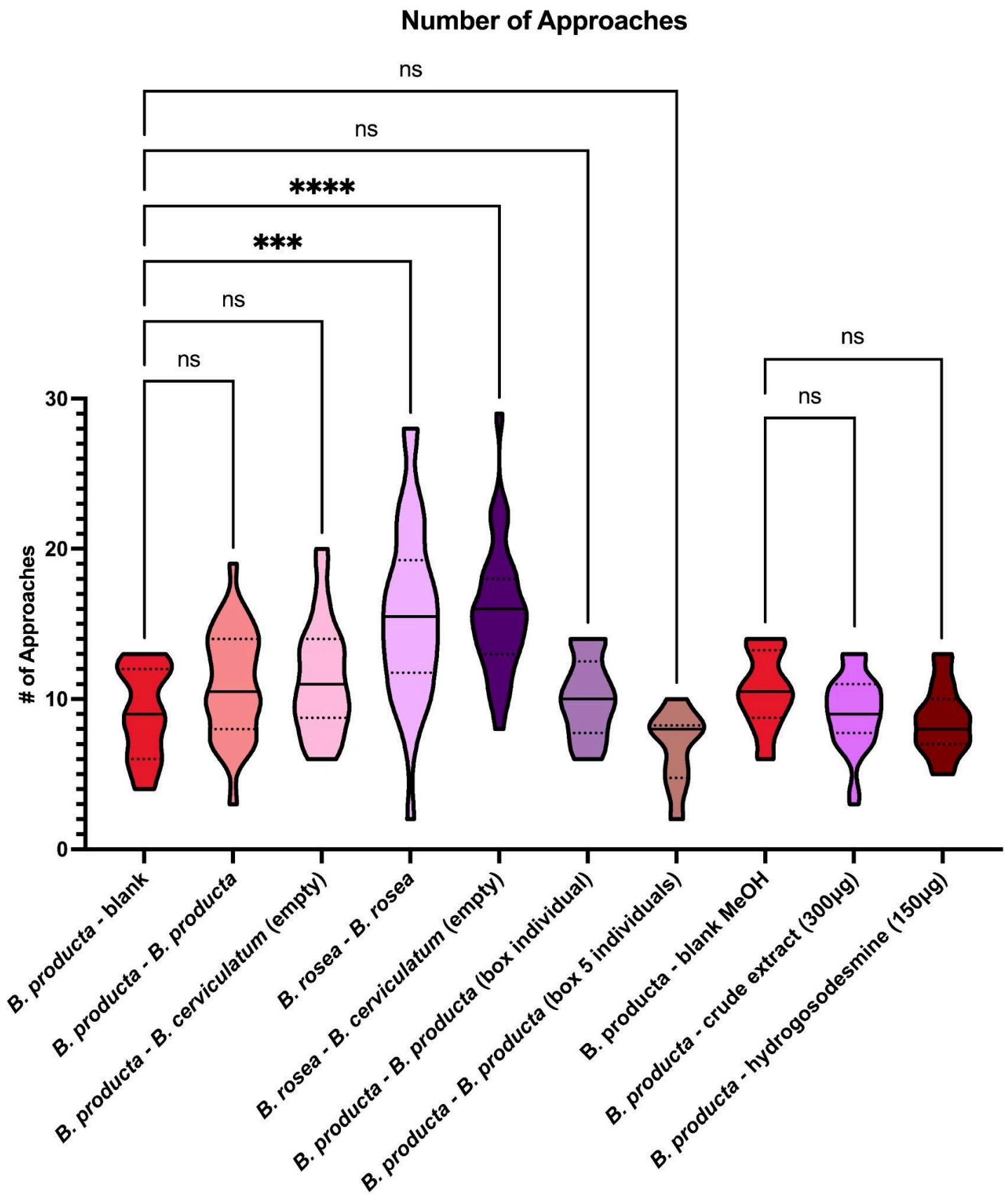

Figure S34. Violin plot of the number of approaches per arm compared with the blank.

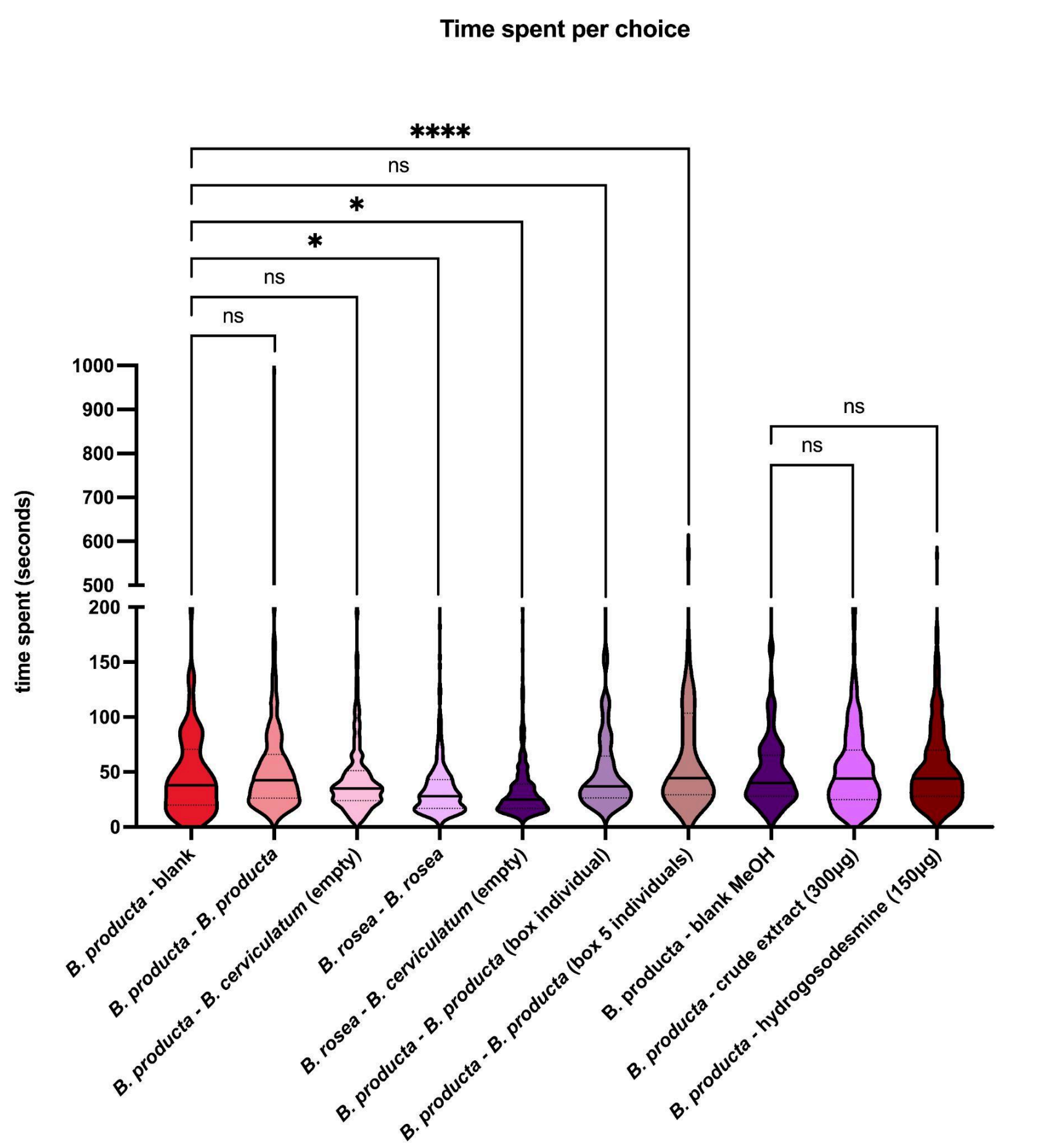

Figure S35. Violin plot for total time spent between effect and empty arms within experiments.

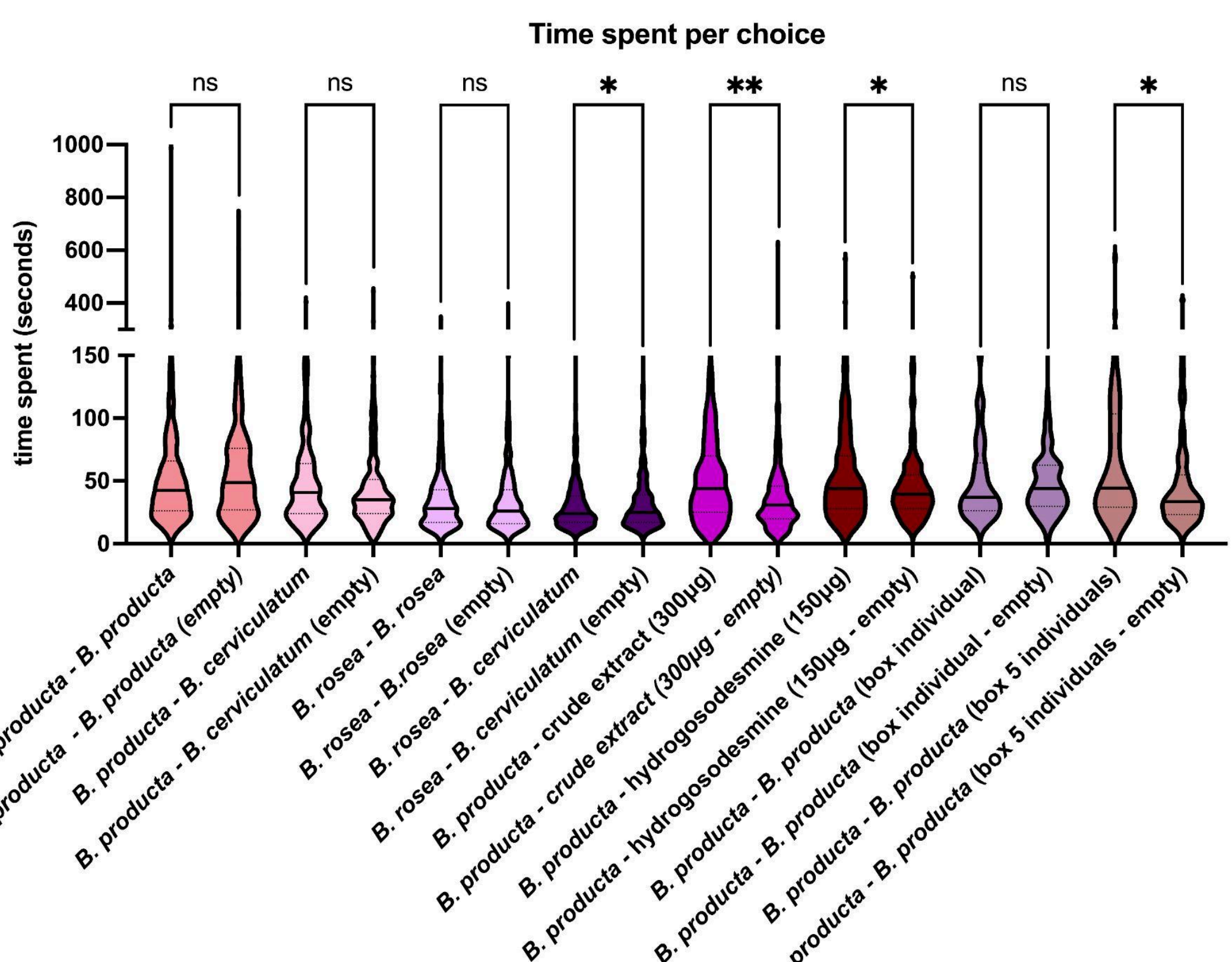

Figure S36. Violin plot for differences between effect and empty arms within experiments.

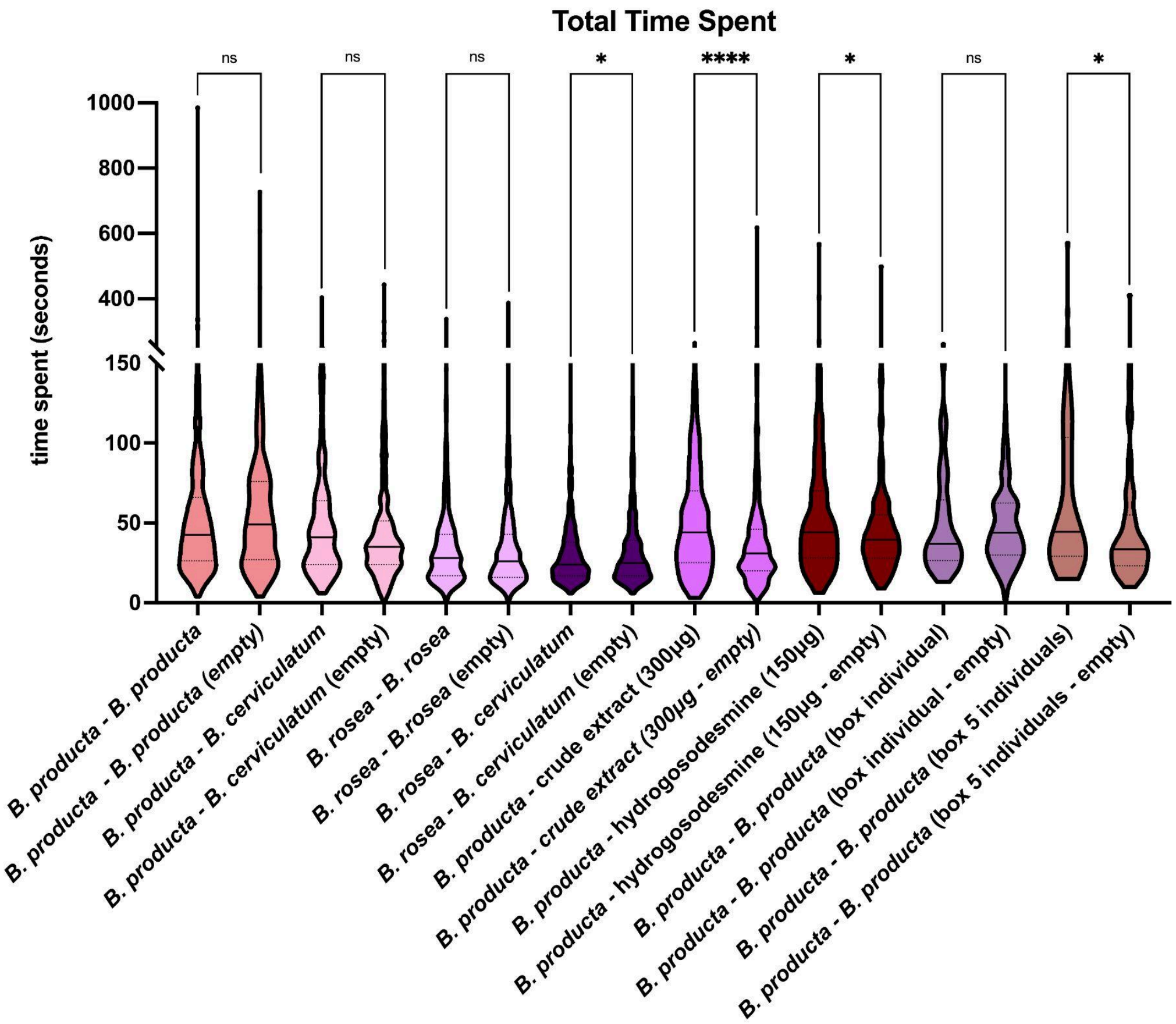

Figure S37. Violin plot for total time spent between the effect and the empty arm within experiments.

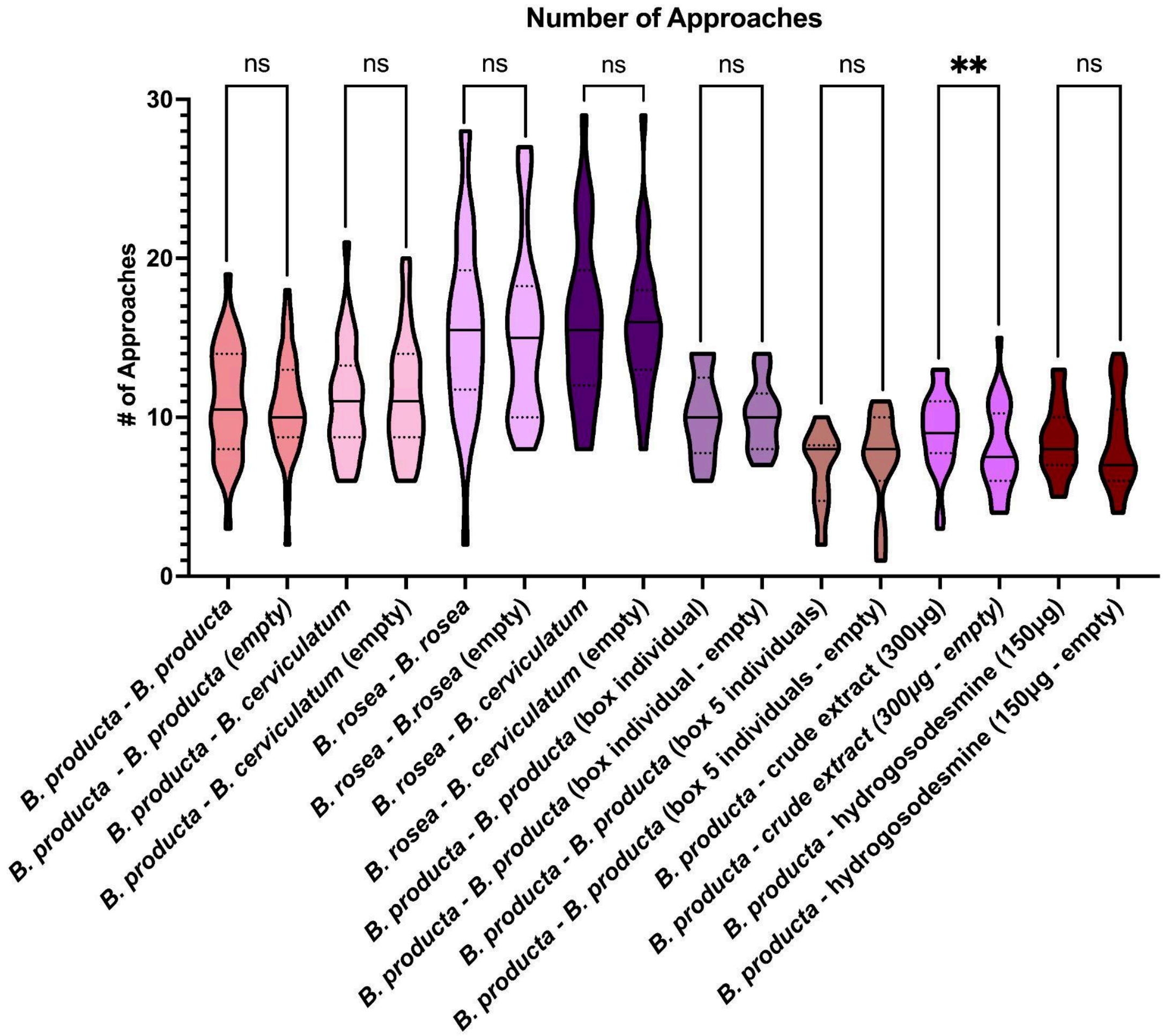

Figure S38. Violin plot for the number of approaches between the effect and the empty arm within experiments.

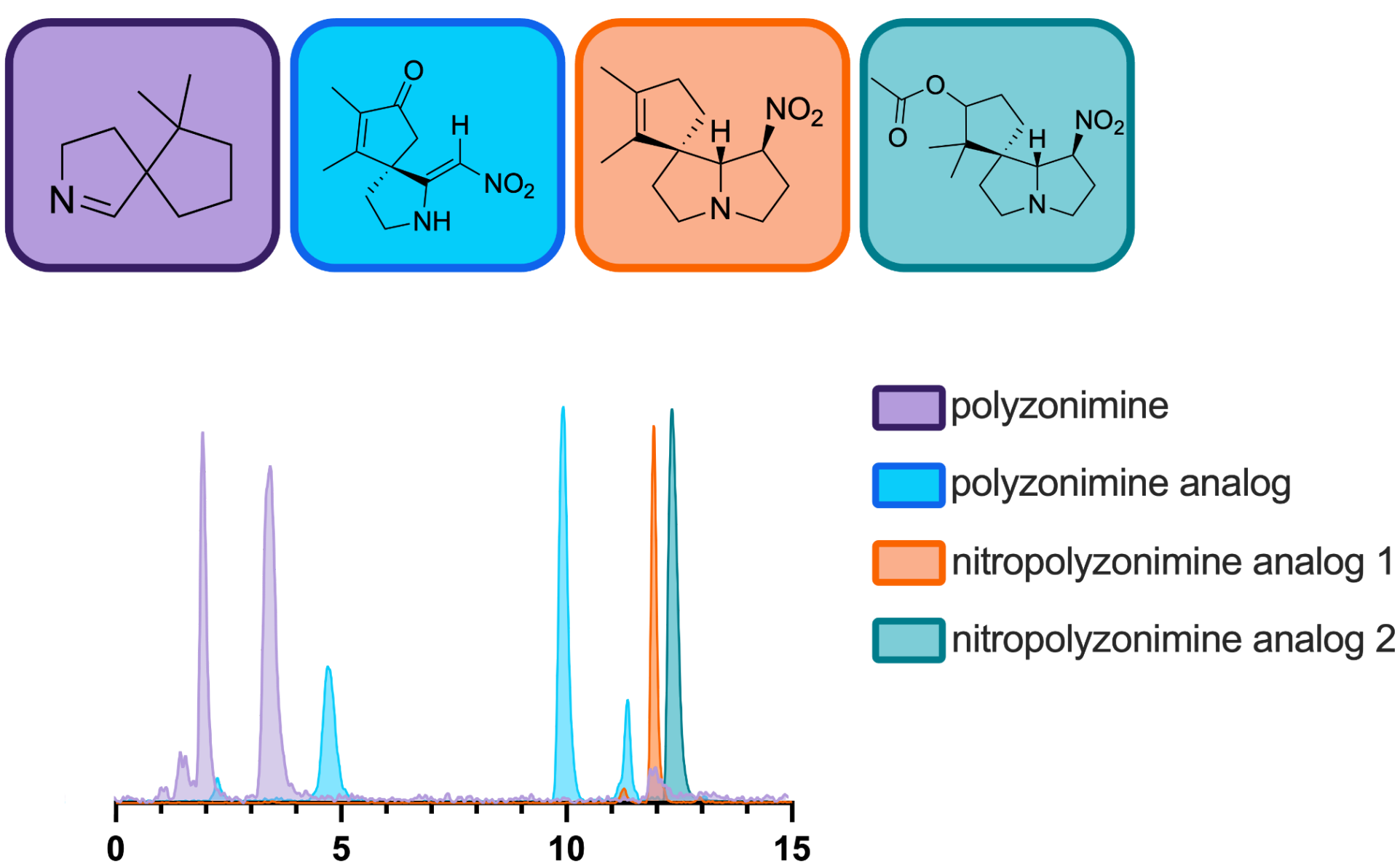

Figure S39. LR-LCMS chromatogram of *B. cerviculatum* crude extract (x-axis is in minutes).

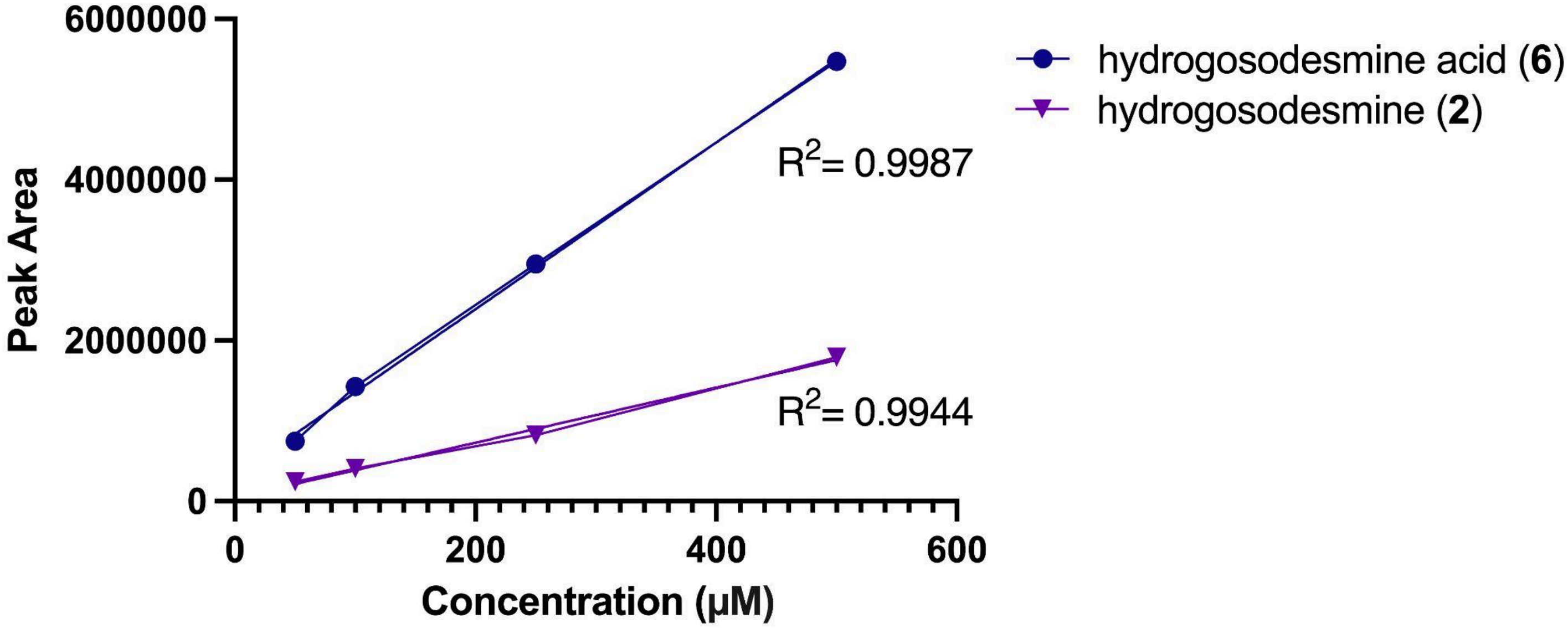

Figure S40. LR-LCMS concentration curve of hydrogosodesmine (**2**) and hydrogosodesmine acid (**6**).
